# Neutrophil extracellular traps induced by chemotherapy inhibit tumor growth in murine models of colorectal cancer

**DOI:** 10.1172/JCI175031

**Published:** 2024-01-09

**Authors:** Yamu Li, Sulin Wu, Yiqing Zhao, Trang Dinh, Dongxu Jiang, J. Eva Selfridge, George Myers, Yuxiang Wang, Xuan Zhao, Suzanne Tomchuck, George Dubyak, Richard T. Lee, Bassam Estfan, Marc Shapiro, Suneel Kamath, Amr Mohamed, Stanley Ching-Cheng Huang, Alex Y. Huang, Ronald Conlon, Smitha Krishnamurthi, Jennifer Eads, Joseph E. Willis, Alok A. Khorana, David Bajor, Zhenghe Wang

**Affiliations:** 1 Department of Genetics and Genome Sciences,; 2 Case Comprehensive Cancer Center,; 3 Department of Internal Medicine,; 4 Department of Medical Genetics, Case Western Reserve University, Cleveland, Ohio. USA.; 5 Seidman Cancer Center, University Hospitals Cleveland Medical Center, Cleveland, Ohio, USA.; 6 Department of Pathology,; 7 Department of Pediatrics, and; 8 Department of Physiology and Biophysics, Case Western Reserve University, Cleveland, Ohio. USA.; 9 Taussig Cancer Institute, Cleveland Clinic, Cleveland, Ohio, USA.; 10 Department of Medicine, University of Pennsylvania, Philadelphia, Pennsylvania, USA.

**Keywords:** Oncology, Colorectal cancer, Neutrophils, Oncogenes

## Abstract

Neutrophil extracellular traps (NETs), a web-like structure of cytosolic and granule proteins assembled on decondensed chromatin, kill pathogens and cause tissue damage in diseases. Whether NETs can kill cancer cells is unexplored. Here, we report that a combination of glutaminase inhibitor CB-839 and 5-FU inhibited the growth of *PIK3CA*-mutant colorectal cancers (CRCs) in xenograft, syngeneic, and genetically engineered mouse models in part through NETs. Disruption of NETs by either DNase I treatment or depletion of neutrophils in CRCs attenuated the efficacy of the drug combination. Moreover, NETs were present in tumor biopsies from patients treated with the drug combination in a phase II clinical trial. Increased NET levels in tumors were associated with longer progression-free survival. Mechanistically, the drug combination induced the expression of IL-8 preferentially in *PIK3CA*-mutant CRCs to attract neutrophils into the tumors. Further, the drug combination increased the levels of ROS in neutrophils, thereby inducing NETs. Cathepsin G (CTSG), a serine protease localized in NETs, entered CRC cells through the RAGE cell surface protein. The internalized CTSG cleaved 14-3-3 proteins, released BAX, and triggered apoptosis in CRC cells. Thus, our studies illuminate a previously unrecognized mechanism by which chemotherapy-induced NETs kill cancer cells.

## Abstract 2

Chemotherapy induces neutrophil extracellular traps, which release cathepsin G that enters cancer cells, cleaves 14-3-3&epsilon;, and triggers apoptosis.

## Graphical abstract

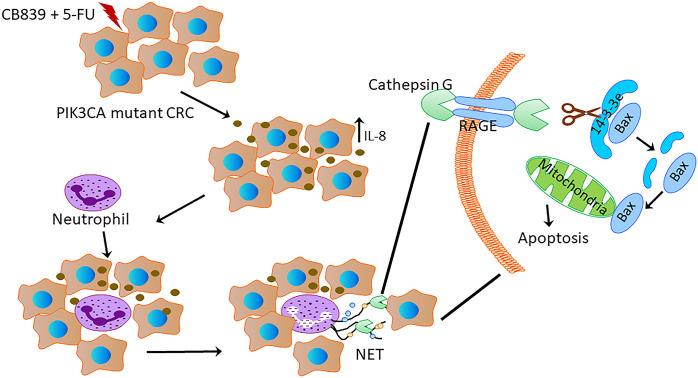

## Introduction

Neutrophils are the most abundant leukocytes in peripheral blood and play a vital role in host defenses against pathogens (1). Neutrophils kill pathogens, including bacteria and fungi, by phagocytosis and by releasing granular enzymes and ROS (1). It was first discovered in 2004 that neutrophils could trap and kill bacteria by releasing Neutrophil extracellular traps (NETs), which consist of decondensed chromatin, granular enzymes, and cytosolic proteins to form web-like structures (2). NET release occurs primarily through a cell death pathway called netosis (1). In the netosis process, nuclear envelopes are dissembled, histones are citrullinated by protein-arginine deiminase 4 (PAD4), which leads to chromatin decondensation, plasma membranes are ruptured, and NETs are released (3). Many proteins, including histone, myeloperoxidase (MPO), neutrophil elastase (NE), proteinase 3 (PR3), and Cathepsin G (CTSG), have been identified in NETs (4). It has been shown that NETs trap and kill bacteria, fungi, viruses, and parasites (3). NETs also cause tissue damage and are involved in autoimmune diseases, liver injury, and thrombosis (3). It remains unknown whether NETs can kill tumor cells, although some studies suggest that NETs promote tumor progression and metastasis (5, 6).

*PIK3CA*, which encodes the p110α catalytic subunit of PI3 kinase (7), is frequently mutated in human cancers, including 30% of colorectal cancers (CRCs) (8). We found that *PIK3CA* oncogenic mutations render CRCs more dependent on glutamine (9). We further demonstrated that a combination of CB-839, a glutaminase inhibitor, and 5-FU, induced *PIK3CA*-mutant CRC regression in multiple xenograft models in nude mice (10). These results prompted clinical trials testing the drug combination in patients with CRC (10). Here, we report a surprising finding that the combination of CB-839 and 5-FU induces NETs, which release serine protease CTSG, in *PIK3CA*-mutant tumors. CTSG enters CRC cells through cell surface protein RAGE, cleaves 14-3-3ε, causes BAX mitochondrial translocation, and induces apoptosis. Moreover, we conducted a phase II clinical trial of a combination of CB-839 and capecitabine, an oral prodrug of 5-FU, in patients with metastatic *PIK3CA*-mutant CRC who were refractory to prior fluoropyrimidine-based chemotherapy. The drug combination increased NETs in a majority of patients’ tumors, which was associated with longer progression-free survival (PFS).

## Results

### Neutrophils modulate the antitumor effect of the combination of CB-839, a glutaminase inhibitor, and 5-FU.

**Figure 1:**
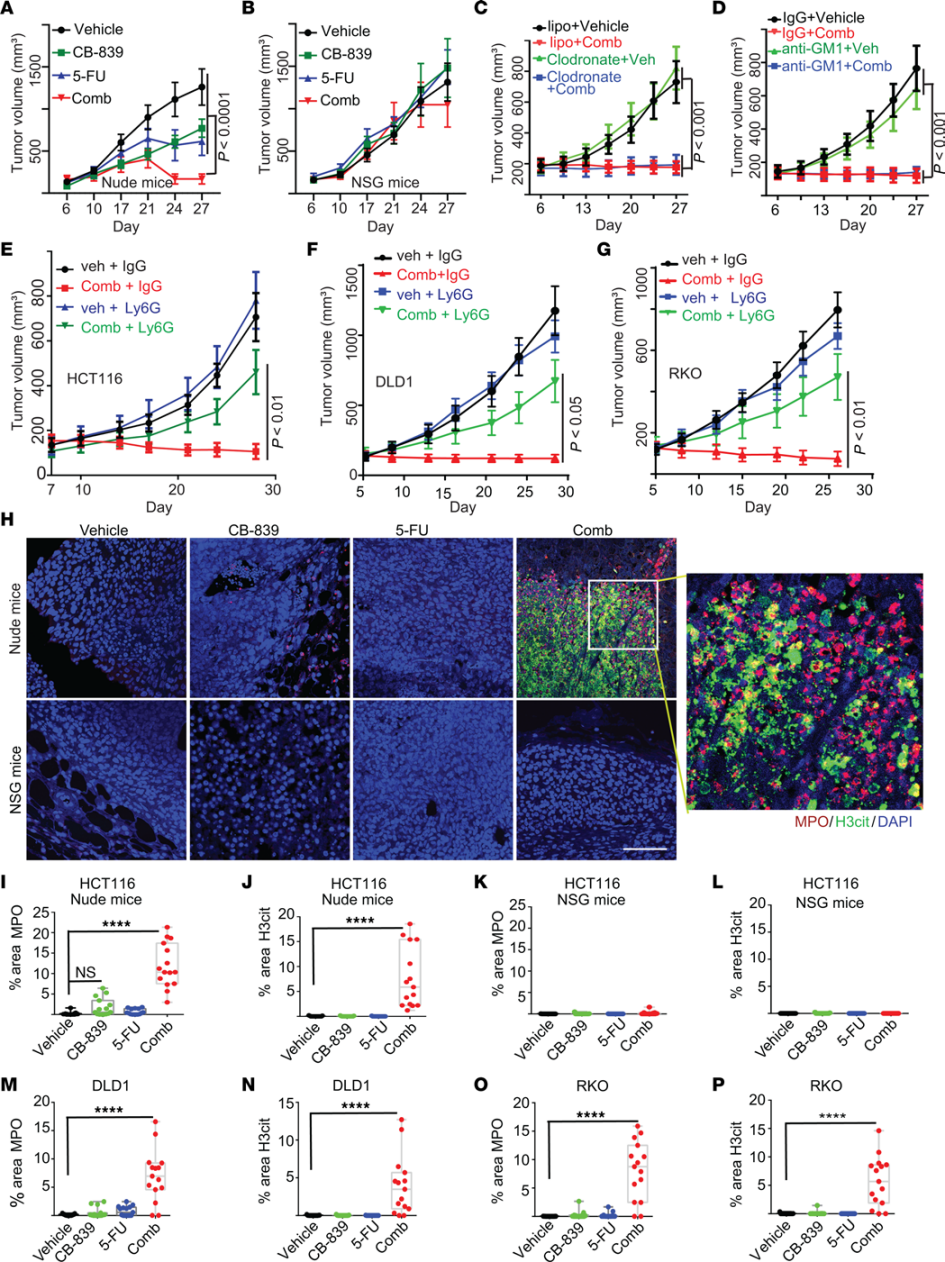
The combination of CB-839 and 5-FU induces NETs in xenograft tumors in nude mice. (**A** and **B**) Xenograft tumors of HCT116 were treated with the indicated drugs 5 days on and 2 days off in nude and NSG mice simultaneously, with the growth curves shown in (**A**) nude mice and (**B**) NSG mice (5 mice/group). (**C** and **D**) Mice were implanted with HCT116 cells, and after 6 days, mice were injected with either 100 μL liposome control or clodronate twice a week (**C**), IgG control or anti-GM1 twice a week (**D**), growth curves shown in **C** for macrophage depletion and **D** for NK cell depletion (5 mice/group). Mice were treated CB-839, 5-FU, and the drug combination daily continuously. (**E**–**G**) CRC xenograft tumors were treated with vehicle (veh) or drug combination (comb) with or without Ly6g antibody injection, tumor sizes were measured, and growth curves are shown in **E** for HCT116, **F** for DLD1, and **G** for RKO (5 mice/group). (**H**–**L**) Tumors shown in **A** and **B** were stained with antibodies against MPO, which marks neutrophils and NETs, and H3cit, which marks NETs. Representative images are shown in **H**. Scale bar: 50 μm, and quantifications are shown in **I**–**L** (*n* = 15/group). (**M**–**P**) Tumors treated with the indicated drugs were stained with antibodies against MPO or H3cit and quantified (*n* = 15/group). The growth curves of the drug treatment were published in Zhao et al. (10). Data in (**A**–**G**) are plotted as mean + SEM. 2-way ANOVA (**A**–**G**) or 1-way ANOVA (**I**–**P**) was used for statistical analysis. *P < 0.05; **P < 0.01; ***P < 0.001; ****P < 0.0001.

Our previous study showed that the combination of CB839 and 5-FU treatment shrunk *PIK3CA*-mutant CRC tumors in xenograft models in nude mice (10). To test if innate immunity could modulate the therapeutic effect of the drug combination, we simultaneously treated nude mice and NOD-SCID-γ (NSG) bearing HCT116 CRC tumors, which harbor a *PIK3CA* mutation, with vehicle control, CB839, 5-FU, or the drug combination (Comb). Consistent with our previous results, the drug combination induced tumor regression in nude mice (Figure 1A). Surprisingly, the drug combination did not cause tumor regression in NSG mice (Figure 1B). These data suggest that innate immune cells modulate the tumor inhibitory effect of the drug combination.

To understand how innate immunity modulates the efficacy of the combination of CB-839 and 5-FU, we first tested if macrophages or NK cells played a role. However, neither the depletion of macrophages nor NK cells altered the tumor inhibitory effect of the drug combination in nude mice (Figure 1, C and D and Supplemental Figure 1, A and B; supplemental material available online with this article; https://doi.org/10.1172/JCI175031DS1). Given that neutrophils were recently reported to have an antitumor effect (11), we next tested if a depletion of neutrophils had any impact on the antitumor effect of the combination of CB-839 and 5-FU. Indeed, the depletion of neutrophils using an anti-Ly6G antibody markedly blunted the tumor inhibitory effect of CB-839 plus 5-FU on HCT116 xenograft tumors (Figure 1E and Supplemental Figure 1, C and D). Similarly, the depletion of neutrophils also attenuated the tumor inhibitory effect of the drug combination in DLD1 and RKO xenograft tumors (Figure 1, F and G). Together, our data suggest that neutrophils modulate the antitumor effect of the drug combination.

However, both nude mice and NSG mice have functional neutrophils, which does not explain the observation that the antitumor effect of the combination of CB-839 and 5-FU was diminished in the NSG mice. Unlike in nude mice, we noted that NSG mice did not tolerate the drug combination well, and we had to give the mice 2-day drug holidays after 5-day drug treatment in NSG and nude mice, which resulted in the initial tumor’s growth at the beginning of the drug combination treatment in nude mice (Figure 1A). These results were different from nude mice treated with the daily drug combination, which caused the continuous shrinkage of the tumors, as we reported previously (10). Thus, we postulated that neutrophils in NSG mice might be more susceptible to the drug combination. We treated nude mice and NSG mice with either vehicle or the combination of CB-839 and 5-FU for 7 days and counted circulating blood cells in these mice without tumors implanted. As shown in Supplemental Figure 1E, compared with vehicle treatment, the relative number of neutrophils substantially decreased in NSG mice treated with the drug combination, whereas the drug combination did not impact neutrophils in nude mice.

### NETs modulate the antitumor effect of the combination of CB-839 and 5-FU.

Given that 2 previous studies have suggested that NETs could suppress tumor growth in vitro (11, 12), we stained the tumors harvested from the experiments shown in Figure 1, A and B with antibodies against citrullinated histone H3 (H3cit), which marks NETs, and MPO, which marks both NETs and neutrophils (5). Interestingly, the drug combination induced NETs in tumors in nude mice but not in NSG mice, quantified using 2 different formulas (Figure 1, H–L and Supplemental Figure 1, F–I). Moreover, tumors in nude mice treated with the drug combination had substantially more neutrophils than tumors treated with vehicle (Figure 1I and Supplemental Figure 1F). In contrast, tumors in NSG mice did not have many infiltrating neutrophils (Figure 1K and Supplemental Figure 1H). Similar results were observed in DLD1 and RKO xenograft tumors in nude mice (Figure 1, M–P and Supplemental Figure 1, J–M). However, the drug treatment had no impact on tumor-infiltrating macrophages and NK cells (Supplemental Figure 1, N–P).

**Figure 2:**
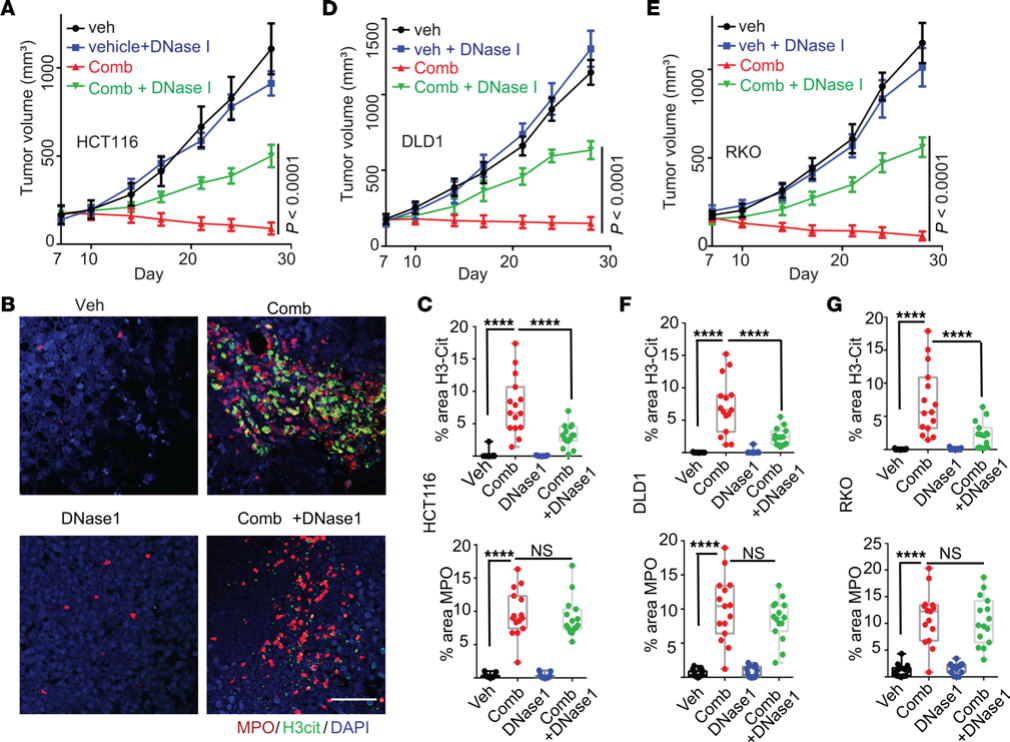
Disruption of NETs by DNase I treatment or depletion of neutrophils attenuates the tumor-inhibitory effect of the combination of CB-839 and 5-FU. (**A**–**G**) The indicated xenograft tumors in nude mice were treated with vehicle (veh) or the drug combination (comb) with or without DNase I (5 mice/group). Tumor growth curves are shown in **A**, **D**, and **E**. Tumors were stained with antibodies against MPO and H3cit. Representative images of HCT116 tumors are shown in **B**. Quantifications are shown in **C**, **F**, and **G**. (*n* = 15/group). Scale bar: 50 μm. 2-way ANOVA (**A**, **D**, and **E**) or 1-way ANOVA (**C**, **F** and **G**) was used for statistical analysis. *P < 0.05; **P < 0.01; ***P < 0.001; ****P < 0.0001.

To determine if NETs induced by the CB-839-plus-5-FU treatment inhibit tumor growth, we set out to deplete NETs using DNase I treatment. We injected HCT116 CRC cells subcutaneously into nude mice. Once tumors reached an average size of 200 mm^3^, mice were randomly assigned to 4 groups and treated by vehicle, vehicle + DNase I, CB-839 + 5-FU, and CB-839 + 5-FU + DNase I. As shown in Figure 2A, DNase I treatment substantially attenuated the tumor inhibitory effect of the combination of CB-839 and 5-FU, whereas DNase I treatment did not impact the growth of tumors treated with vehicle. Immunofluorescence staining of the tumors showed that DNase I drastically reduced levels of H3cit — the NET marker — but did not impact MPO levels — the neutrophil marker — in tumors treated with the combination of CB-839 and 5-FU (Figure 2, B and C, and Supplemental Figure 2A). Similarly, DNase I treatment disrupted NETs and attenuated the tumor inhibitory effect of the combination of CB-839 and 5-FU in DLD1 and RKO xenografts tumors (Figure 2, D–G and Supplemental Figure 2, B and C). Moreover, a NE inhibitor, sivelestat, which was shown to block NET formation (13), also attenuated the antitumor effect of the drug combination (Supplemental Figure 2D) and reduced the levels of H3cit in the tumors (Supplemental Figure 2E). Consistently, the depletion of neutrophils drastically reduced levels of both H3cit and MPO in tumors treated with CB-839 plus 5-FU (Supplemental Figure 2, F–L). Together, these data suggest that NETs modulate the antitumor effect of the drug combination.

### The combination of CB-839 and 5-FU upregulates IL-8 in PI3KCA-mutant tumors to recruit neutrophils.

**Figure 3:**
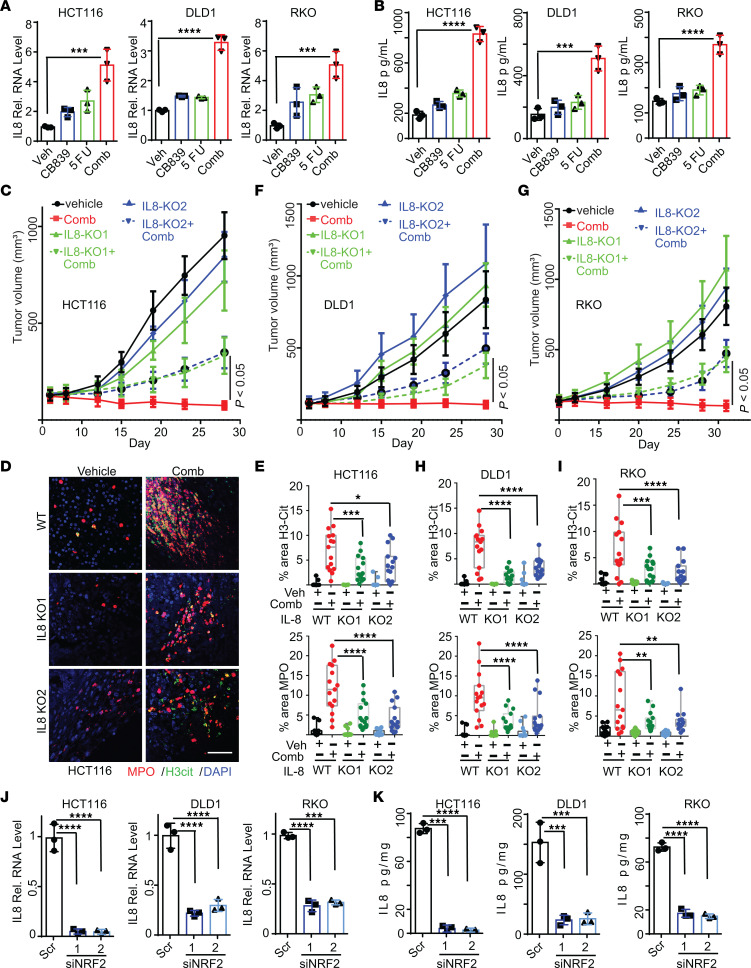
The combination of CB-839 and 5-FU induces IL-8 expression in CRCs to attract tumor-infiltrating neutrophils. (**A** and **B**) The indicated cells were treated with the indicated drugs. IL-8 mRNA and protein levels were measured by qRT-PCR (A) and ELISA (**B**), respectively, (*n* = 3/group). (**C–I**) Parental HCT116, DLD1, RKO, and their *IL-8* KO clones were grown as xenograft tumors in nude mice and treated with vehicle or the combination of CB-839 and 5-FU (5 mice/group). Tumor growth curves are shown in (**C**, **F**, and **G**). Tumors were stained with antibodies against MPO and H3cit. Representative images of HCT116 tumors are shown in (**D**). Quantifications are shown in (**E**, **H** and **I**), (*n* = 15/group). (**J** and **K**) The indicated CRC cells were transfected with scramble siRNA or 2 independent siRNA against IL-8. IL-8 mRNA levels are shown in (**J**), and secreted IL-8 levels are shown in (**K**), (*n* = 3/group). Scale bar: 50 μm. 2-way ANOVA (**C**, **F**, **G**, **E**, **H**, and **I**) or 1-way ANOVA (**A**, **B**, **J**, and **K**) was used for statistical analysis. *P < 0.05; **P < 0.01; ***P < 0.001; ****P < 0.0001.

We next set out to determine how the combination of CB-839 and 5-FU induces NETs. We first performed RNA-Seq on isogenic DLD1 *PIK3CA* WT-only and Mut-only cell lines (14) treated with either vehicle or the combination of CB-839 and 5-FU, because the drug combination induced more NETs and tumor-infiltrating neutrophils in *PIK3CA*-Mut–only tumors than the isogenic WT-only tumors (Supplemental Figure 3, A and B). Over 125 genes were differentially expressed in *PIK3CA*-Mut–only cells upon the combinational drug treatment (Supplemental Figure 3, C and D and Supplemental Table 1). Pathway analyses revealed that the inflammatory response pathway, which includes the neutrophil chemokine IL-8, was one of the most significantly enriched pathways (Supplemental Figure 3E). Second, we profiled cytokines in the isogenic DLD1 *PIK3CA*-WT–only and Mut-only cell lines. Again, the drug combination induced more IL-8 in *PIK3CA*-Mut–only cells than in the isogenic *PIK3CA*-WT–only cells (Supplemental Figure 4F). Other neutrophil chemokines, including CXCL1, CXCL2, and CXCL5, were not upregulated by the drug treatment (Supplemental Figure 3G). Moreover, the drug combination induced IL-8 in parental HCT116, DLD1, and RKO cultured cells, as well as in xenograft tumors (Figure 3, A and B and Supplemental Figure 3H). It is worth noting that it has been demonstrated by many studies that human IL-8 can act as chemokine to attract mouse neutrophils (15–20).

To test if the drug combination–induced IL-8 attracts neutrophils and modulates the tumor-inhibitory effect, we knocked out IL-8 in 3 different CRC cell lines, including HCT116, DLD1, and RKO (Supplemental Figure 3, I and J). Two independently derived *CXCL8*/*IL-8*–KO clones from each cell line were used for in-depth analyses. As shown in Figure 3, C–E, *IL-8* KO attenuated the tumor-inhibitory effect of the combination of CB-839 and 5-FU and reduced tumor-infiltrating neutrophils and NETs in HCT116 xenograft tumors. Similar results were observed with *IL-8*–KO DLD1 and RKO cells (Figure 3, F–I). As a control, we knocked out *CXCL1* in HCT116 cells (Supplemental Figure 3, K and L), which did not impact the xenograft tumor growth (Supplemental Figure 3M). The *CXCL1* KO did not attenuate the antitumor effect of the drug combination (Supplemental Figure 3M). Moreover, to test if a gene knockout that leads to reduced tumor growth would impact the antitumor effect of the drug combination, we chose to treat *ERBB3*-KO tumors, which resulted in reduced xenograft tumor growth (21). As shown in Supplemental Figure 3, N and O, KO of ERBB3 did not attenuate the antitumor effect of the drug combination. Together, these results suggest that the combination of CB-839 and 5-FU induced IL-8 expression in cancer cells, which attracts neutrophils to form NETs and augments the antitumor effect of the drug combination.

### The combination of CB-839 and 5-FU activates NRF2 to induce IL-8 gene transcription.

We next interrogated how IL-8 was induced by the combination of CB-839 and 5-FU in CRC cells. qRT-PCR analyses showed that IL-8 mRNA expression was substantially upregulated in HCT116, DLD1, and RKO CRC cells by the drug combination (Figure 3A). It was reported that NRF2 is a transcription factor for IL-8 (22). We have demonstrated that the combination of CB-839 and 5-FU induces ROS and activates NRF2 transcriptional activity preferentially in *PIK3CA*-mutant CRCs compared with isogenic *PIK3CA*-WT CRCs (10). Thus, we knocked down NRF2 in HCT116, DLD1, and RKO cells with 2 independent siRNA (Supplemental Figure 3P). Figure 3, J and K show that the knockdown of NRF2 substantially reduced NRF2 mRNA and protein expression induced by the combination of CB-839 and 5-FU in HCT116, DLD1, and RKO cells. To further validate these results, we knocked out *NRF2* in HCT116 and DLD1 cells (Supplemental Figure 3, Q and R). Consistent with the siRNA knockdown results, the KO of *NRF2* substantially reduced the drug-induced IL-8 expression (Supplemental Figure 3S). We failed to obtain any *NRF2* KO clones in RKO cells, suggesting that NRF2 may be an essential gene for RKO. There are 6 putative NRF2 binding sites in the promoter region of the *IL-8* gene. ChIP-qPCR analyses indicated that the drug combination induced NRF2 binding to 3 of them (Supplemental Figure 3T). Together, the data suggest that the combination of CB-839 and 5-FU activates NRF2 transcriptional activity in CRC cells and induces IL-8 expression, thereby attracting neutrophils into tumors and producing NETs to inhibit tumor xenograft tumor growth.

### The combination of CB-839 and 5-FU increases ROS levels in neutrophils to induce NETs.

**Figure 4:**
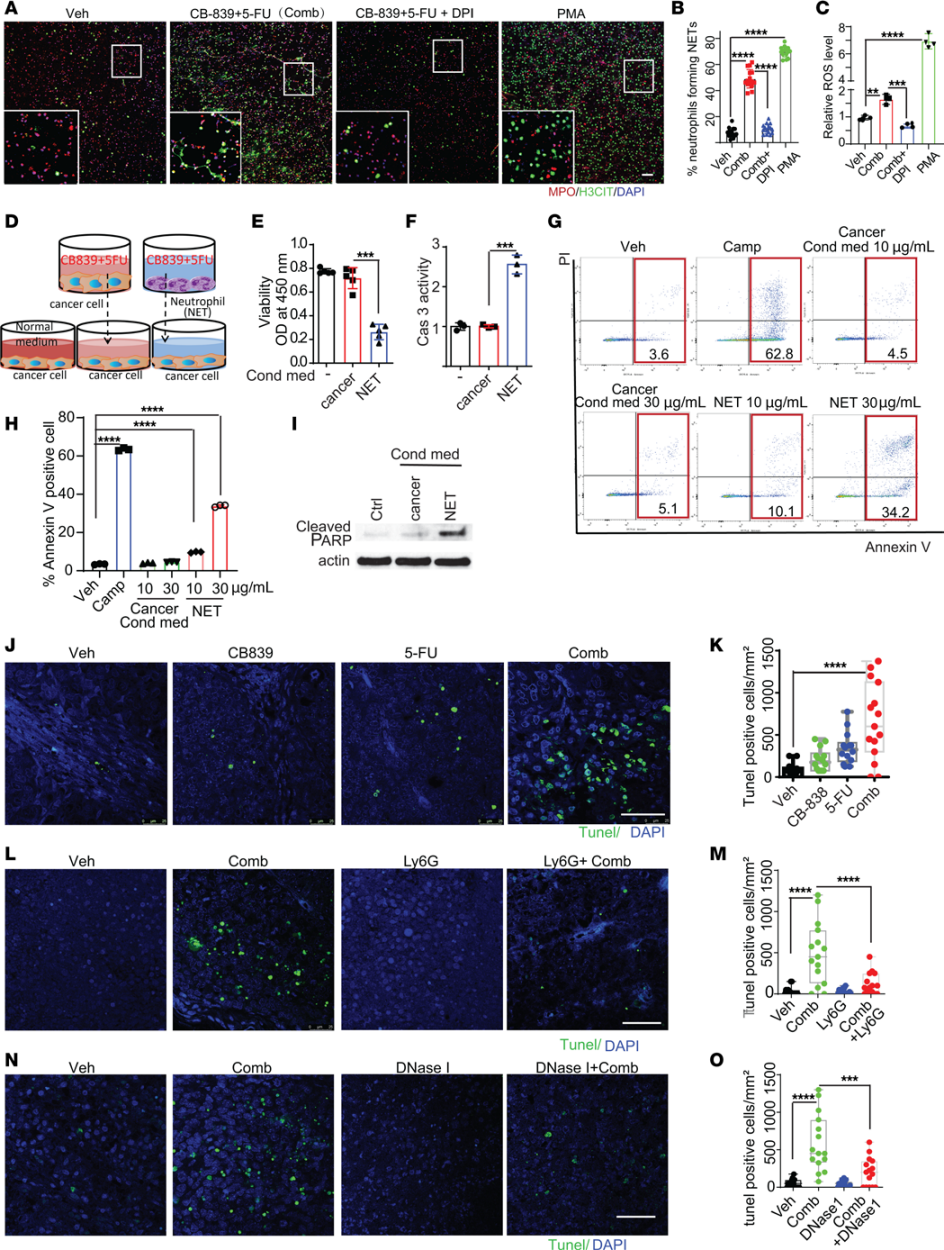
The combination of CB-839 and 5-FU acts on neutrophils to induce NETs and cause apoptosis in cancer cells. (**A**–**C**) Purified neutrophils were treated with the indicated compounds and stained with antibodies against MPO and H3cit. Representative images are shown in **A**, and quantifications are shown in **B**, (*n* = 15/group). The levels of ROS are shown in **C** (*n* = 4/group). Diphenyleneiodonium (DPI, 2 μM) is a ROS scavenger. Neutrophils were treated with phorbol myristate acetate (PMA, 1 μM) as a positive control. (**D**–**I**) (**D**) Schematics of the experiment setup. Cancer cells or neutrophils (NET) were treated with the combination of CB-839 and 5-FU, the conditioned media were collected, and protein concentrations of the conditioned media were measured. The indicated conditioned media were diluted to a final concentration of 10 μg/mL, or 30 μg/mL in fresh McCoy’s 5A medium to treat HCT116 cancer cells overnight. Cell viabilities are shown in **E** (*n* = 5/group), caspase 3 activities are shown in **F** (*n* = 3/group), annexin V positive cells are shown in **G** and **H**, (*n* = 3/group) and cleaved PARPs are shown in **I**. HCT116 cells were treated with camptothecin (3 μM) as a positive control. (**J**–**O**) Tunel staining was performed on the HCT116 xenograft tumors with indicated treatment. Representative images of HCT116 tumors are shown in **J**, **L**, and **N**, and quantifications are shown in **K**, **M**, and **O** (*n* = 15/group). Scale bar: 50 μm. 1-way ANOVA (**B**, **C**, **E**, **F**, **H**, **K**, **M** and **O**) was used for statistical analysis. *P < 0.05; **P < 0.01; ***P < 0.001; ****P < 0.0001.

To determine how the combination of CB-839 and 5-FU induces NETs, we isolated and purified neutrophils from the bone marrow of C57/BL6J mice (Supplemental Figure 4A) and treated them with DMSO, CB-839, 5-FU, or the drug combination. Neutrophils were treated with PMA in parallel as a positive control. As shown in Figure 4, A and B and Supplemental Figure 4, B–D, CB-839 induced a small number of NETs (fiber-like DNA coated with H3cit and MPO), whereas 5-FU induced a nominal number of NETs. However, the drug combination induced many web-like NETs with long DNA fibers (Figure 4, A and B and Supplemental Figure 4, B–D). Interestingly, the PMA treatment induced a large amount of H3cit (Figure 4A). However, NETs induced by PMA lacked long DNA fibers (Supplemental Figure 4D). The drug-induced NET formation was dependent on PAD4, because the PAD4 inhibitor GSK484 blocked the NET formation induced by the drug combination (Supplemental Figure 4E).

It was reported that IL-8 could induce NETs (23). Given that we have shown that the drug combination induces IL-8 expression in CRC cells, we then treated the purified neutrophils with various amounts of IL-8. However, IL-8 failed to induce NETs in this setting (Supplemental Figure 4, F and G), which is consistent with previous reports (24, 25).

It is well documented that ROS induces NETs in neutrophils. Given that we reported previously that the combination of CB-839 and 5-FU induces ROS (10), we set out to determine if the drug combination induces NETs through ROS production. Indeed, the combination of CB-839 and 5-FU substantially increased ROS levels in neutrophils (Figure 4C), although it was a weaker ROS inducer than PMA. Nonetheless, ROS scavenger diphenyleneiodonium (DPI) reduced ROS levels in neutrophils and the amount of NETs induced by the drug combination (Figure 4C). Together, the in vitro data suggest that the combination of CB-839 and 5-FU induces NETs through ROS production in neutrophils. However, we cannot rule out the possibility that ROS acts in concert with other factors generated by the drug combination to produce NETs.

To interrogate how NETs inhibit CRCs, we treated neutrophils with the combination of CB-839 and 5-FU to induce NETs, and conditioned medium (NET medium) from this culture was transferred to a new well containing HCT116 CRC cells (Figure 4D). Compared with cells grown in a normal medium or conditioned medium from HCT116 cells treated with the drug combination, the NET medium inhibited HCT116 cell growth and induced apoptosis in a dose-dependent manner (Figure 4, E–I). Although we reported previously that high doses of CB-839 (20 μM) and 5-FU (10 μM) combination induced apoptosis of HCT116 cells, the low doses of CB-839 (1 μM) and 5-FU (1 μM) we used here for NET induction did not increase apoptosis compared with controls (Supplemental Figure 4H). Moreover, conditioned media from neutrophils without drug treatment or treated with the neutrophil activator N-Formylmethionyl-leucyl-phenylalanine (fMLP) did not induce apoptosis in HCT116 cells (Supplemental Figure 4I). Consistently, the combination of CB-839 and 5-FU treatment substantially increased apoptosis in HCT116, DLD1, and RKO xenograft tumors in nude mice (Figure 4, J and K and Supplemental Figure 4, J and K). We have shown that either DNase I treatment or neutrophil depletion attenuated the antitumor effect of the drug combination. Consistent with these results, DNase I treatment or neutrophil depletion substantially reduced apoptosis induced by the drug combination in xenograft tumors (Figure 4, L–O and Supplemental Figure 4, L and M). The data suggest that components in NETs may kill CRC cells by inducing apoptosis.

We next treated neutrophils with various chemotherapy drugs to test if other cancer drugs could induce NETs. Supplemental Figure 4, N and O show that camptothecin, gemcitabine, daunorubicin, epirubicin, and regorafenib also induced NETs, suggesting that NET induction may be a mechanism by which some chemotherapies inhibit tumor growth.

### CTSG in NETs kills cancer cells.

**Figure 5:**
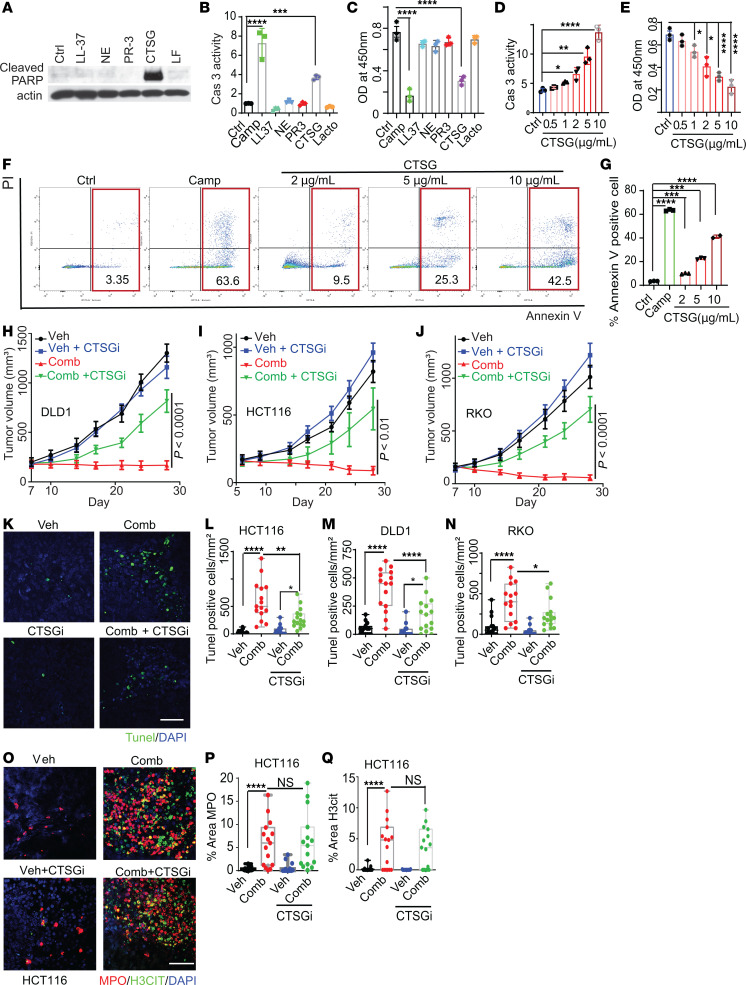
CTSG in NETs inhibits tumor growth. (**A**–**C**) The HCT116 CRC cells were treated with the indicated recombinant proteins (10 μg/mL) overnight. Cell lysates were blotted with the indicated antibodies (**A**). The HCT116 cells were treated with the indicated recombinant proteins. Cells were treated with camptothecin (3 μM) in parallel as a positive control. Caspase 3 activities were measured (**B**) (*n* = 3/group). Cell numbers are shown in **C** (*n* = 3/group). (**D**–**G**) HCT116 cells were treated with indicated concentrations of recombinant CTSG. Caspase 3 activities are shown in **D** (*n* = 3/group), cell numbers were counted in **E** (*n* = 3/group). Annexin V staining and quantifications are shown in **F** and **G** (*n* = 3/group). (**H**–**J**) The indicated xenograft tumors were established in nude mice and treated with vehicle or the drug combination with or without CTSGi (intratumor injection, 5 mice/group). (**K**–**N**) Tunel staining of the tumors is shown in **H**–**J**. Representative images of HCT116 tumors are shown in **K**. Quantifications are shown in **L**–**N** (*n* = 15/group). (**O**–**Q**) Tumors from **H** stained with anti-MPO and anti-H3cit antibodies. Representative images of HCT116 tumors are shown in **O**, and quantifications are shown in **P** and **Q** (*n* = 15/group). Scale bar: 50 μm. 1-way ANOVA (**B**–**E**, **G**, **L**–**N**, **P**, and **Q**) or 2-way ANOVA (**H**–**J**) was used for statistical analysis. *P < 0.05; **P < 0.01; ***P < 0.001; ****P < 0.0001.

NETs consist of web-like DNA and associated granular proteins (1). To test if any of the proteins in NETs could induce apoptosis of CRC cells, we treated HCT116 cells with recombinant LL-37, NE, protein 3 (PR-3), CTSG, or lactoferrin (LF). As shown in Figure 5, A–G and Supplemental Figure 5A, only CTSG, a serine protease, inhibited HCT116 cell growth and induced apoptosis in a dose-dependent manner. Interestingly, the NET medium contains CTSG (Supplemental Figure 5B). In contrast, conditioned media of neutrophils treated with either fMLP or PMA had a nominal amount of CTSG, which explains that conditioned media from both treatments did not induce much apoptosis (Supplemental Figure 4I and Supplemental Figure 5, B–D). These data suggest that NETs induced by the combination of CB-839 and 5-FU may be distinct from NETs induced by PMA. Consistently, NETs induced by the drug combination had more DNA fibers than NETs induced by PMA (Supplemental Figure 4D). Moreover, a noncell-permeable CTSG inhibitor I (CTSGi) (26), which potently inhibits CTSG protease activity, attenuated apoptosis induced by either recombinant CTSG or conditioned medium from neutrophils treated with CB-839 and 5-FU (NET medium) in a dose-dependent manner (Supplemental Figure 5, E–K) in HCT116, DLD1, and RKO CRC cells. In contrast, an NE inhibitor sivelestat only marginally inhibited apoptosis of colon cancer cells treated with NET conditioned medium (Supplemental Figure 5, L and M). The marginal effect of sivelestat may be due to its low activity in inhibiting CTSG (27). Together, those data suggest that CTSG released from NETs kills CRC cells.

To test if CTSG plays a critical role in the tumor inhibitory effect of the combination of CB-839 and 5-FU mediated by NETs in vivo, we treated HCT116 xenograft tumors established subcutaneously in nude mice with vehicle control or the drug combination with or without CTSGi. As shown in Figure 5H, the CTSGi substantially attenuated the tumor inhibitory effect of CB-839 and 5-FU, whereas the inhibitor had no impact on tumors treated with vehicle. Consistently, CTSGi treatment reduced apoptosis in tumors treated with the drug combination (Figure 5, K and L). Similar results were observed with RKO and DLD1 xenograft tumors (Figure 5, I, J, M, and N). Moreover, the CTSGi did not reduce levels of NETs induced by the combinational drug treatment (Figure 5, O–Q and Supplemental Figure 5, N–T). Given that we have shown that DNase I treatment attenuated the antitumor effect of the drug combination (Figure 2A), we postulated that the decondensed DNAs in NETs anchor CTSG within tumors, without which CTSG proteins could be washed out. In support of this, the Western blot analyses showed that the drug combination–induced CTSG protein levels were reduced in tumors treated with DNase I (Supplemental Figure 5, U and V).

### CTSG enters cancer cells through RAGE.

**Figure 6:**
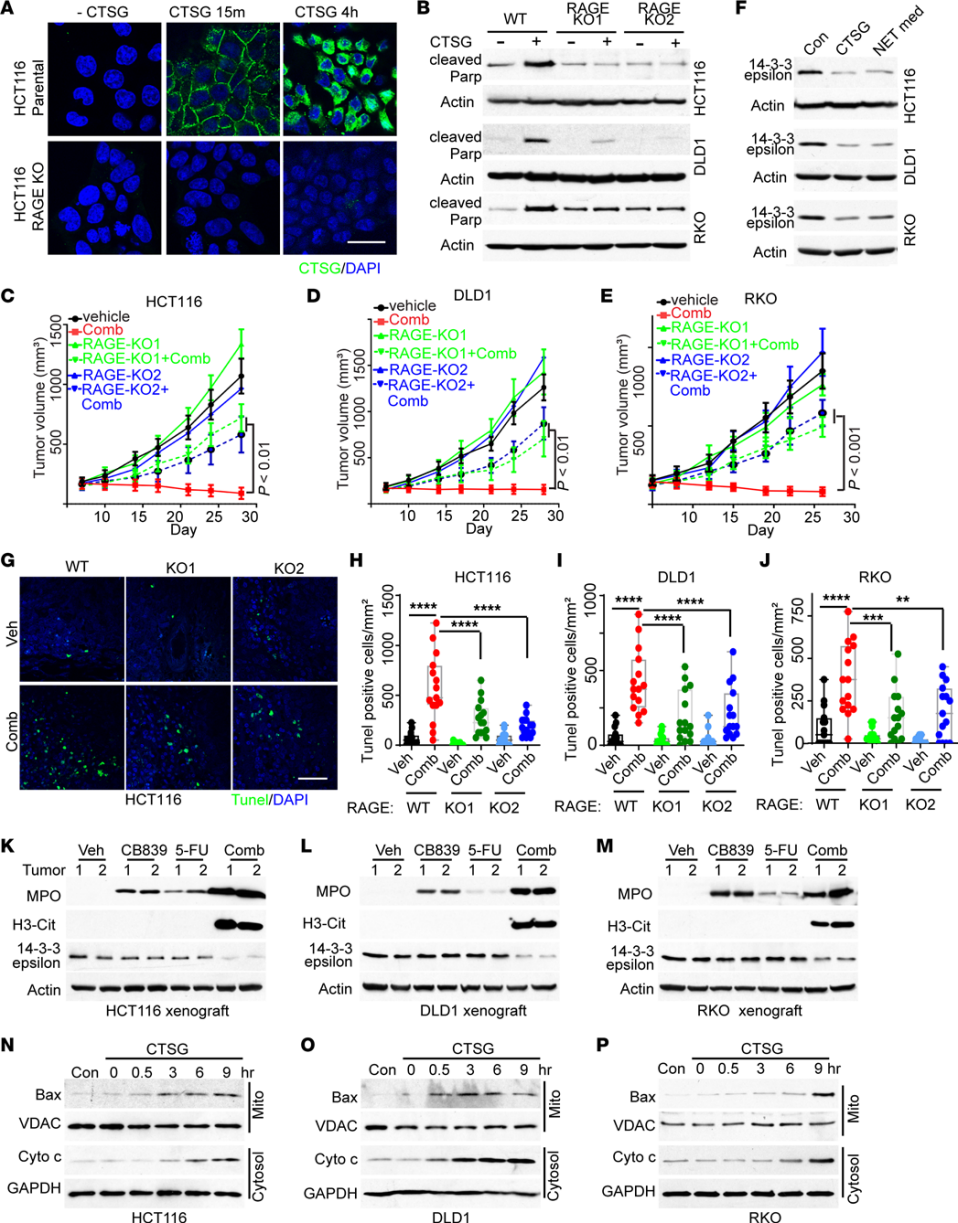
CTSG enters cancer cells through RAGE, cleaves 14-3-3ε, results in BAX mitochondrial translocation, and triggers apoptosis. (**A** and **B**) Parental HCT116, DLD1, RKO, and their RAGE KO cells were incubated with 5μg/mL recombinant CTSG for the indicated time. Representative images of immunofluorescent staining of an anti-CTSG antibody are shown in **A**, and Western blots of cleaved PARP are shown in **B**. (**C**–**E**) CRC RAGE WT and KO tumors treated with vehicle or drug combination, tumor growth curves are shown in **C** for HCT116, **D** for DLD1, and **E** for RKO (5 mice/group). (**F**) CRC cells were treated with recombinant CTSG or NET-conditioned medium for 16 hours, and cell lysates were blotted with indicated antibodies. Corrected loading control provided. **(G**–**J)** Tunel staining was performed on the CRC xenograft tumors from **C**–**E**. Representative images of HCT116 tumors are shown in **G**, and quantifications are shown in **H** for HCT116, **I** for DLD1, and **J** for RKO (*n* = 15/group). (**K**–**M**) Western blot of 14-3-3ε protein levels in the tumors with indicated treatment. (**N**–**P**) CRC cells were treated with 5μg/mL CTSG for the indicated time, and mitochondrial and cytosolic fractions were extracted by a cell fractionation kit and blotted with the indicated antibodies. 2-way ANOVA (**C**–**E** and **H**–**J**) was used for statistical analysis. *P < 0.05; **P < 0.01; ***P < 0.001; ****P < 0.0001.

To test if CTSG can enter CRC cells to trigger apoptosis, we incubated recombinant CTSG with HCT116 cells. Immunofluorescent staining showed that CTSG was on the cell surface 15 minutes after CTSG was added (Figure 6A) and predominantly inside cells after 4 hours (Figure 6A). Given that CCDC25 is reported to be a receptor for NET DNA (28), we tested whether CTSG enters cancer cells through CCDC25-mediated NET DNA-CTSG complex internalization. We knocked out *CCDC25* in HCT116 and DLD1 cells using CRISPR/Cas 9–mediated genome editing (Supplemental Figure 6A). However, the knockout of *CCDC25* did not impair the entrance of CTSG into cancer cells (Supplemental Figure 6B). Moreover, the knockout of *CCDC25* did not affect CTSG-induced apoptosis in both HCT116 and DLD1 cells (Supplemental Figure 6C), nor did it impact the antitumor effect of the drug combination (Supplemental Figure 6D). These results suggest that the NET DNA-CCDC25 pathway is not involved in the CTSG-induced apoptosis of CRC cells. We next turned our attention to the cell surface protein, RAGE, because it was shown that RAGE mediated neutrophil-derived CTSG cytotoxicity (29). We knocked out *RAGE* in HCT116, DLD1, and RKO CRC cells using CRISPR/Cas 9–mediated genome editing (Supplemental Figure 6F). Indeed, the KO of *RAGE* blocked the entrance of CTSG into HCT116, DLD1, and RKO cells (Figure 6A and Supplemental Figure 6G). Moreover, KO of *RAGE* abrogated apoptosis induced by CTSG or NET medium in HCT116, DLD1, and RKO cells (Figure 6B and Supplemental Figure 6H). In contrast, PR3 did not enter any of the CRC cells (Supplemental Figure 6I). NE failed to enter HCT116 and RKO CRC cells, although it entered DLD1 cells (Supplemental Figure 6I). However, *RAGE* KO did not prevent NE from entering DLD1 cells.

Next, we tested if the KO of *RAGE* attenuated the tumor-inhibitory effect of the combination of CB-839 and 5-FU. As shown in Figure 6, C–E, the drug combination did not induce tumor regression of *RAGE*-KO HCT116, DLD1, and RKO CRCs. Consistently, *RAGE* KO attenuated the drug combination–induced apoptosis in tumors (Figure 6, G–J). Together, the data suggest that RAGE mediates the entrance of CTSG into CRC cells.

### CTSG cleaves 14-3-3ε, induces BAX mitochondrial translocation, and triggers apoptosis.

Given that CTSG is a serine protease, we postulated that CTSG might cleave an antiapoptotic protein to trigger apoptosis. However, neither BCL2 nor VDAC was cleaved when HCT116 cells were incubated with recombinant CTSG (Supplemental Figure 7A). We then turned our attention to 14-3-3 proteins because they were predicted to be a substrate of CTSG. Moreover, 14-3-3 proteins were shown to bind and sequester BAX from mitochondria, thereby preventing apoptosis (30). Indeed, 14-3-3ε protein levels were decreased when HCT116 cells were incubated with recombinant CTSG (Figure 6F). In contrast, CTSG had either no or marginal effect on other 14-3-3 isoforms (Supplemental Figure 7A). Moreover, 14-3-3ε protein levels were decreased when CRC cells were incubated with NET medium (Figure 6F and Supplemental Figure 7A). We then tested if CTSG could cleave 14-3-3ε in vitro. As shown in Supplemental Figure 7B, CTSG cleaves 14-3-3ε in a time-dependent manner. Similarly, 14-3-3ε can be cleaved by incubating with NET medium, and this cleavage could be suppressed by CTSGi (Supplemental Figure 7C), but not DNase I (Supplemental Figure 7D). Furthermore, compared with vehicle controls, 14-3-3ε protein levels were also decreased in HCT116 xenograft tumors treated with the combination of CB-839 and 5-FU (Figure 6K and Supplemental Figure 7E). Consistent with the notion that the drug combination induces NETs, the combination of CB-839 and 5-FU treatment induced higher levels of H3-cit and MPO in the HCT116 xenograft tumors (Figure 6K and Supplemental Figure 7E). Similar results were observed with DLD1 and RKO cells and their xenografts (Figure 6, L and M and Supplemental Figure 7, F and G). Together, these data suggest that CTSG enters CRC cells, cleaves 14-3-3ε, and triggers apoptosis.

Given that 14-3-3 bind to BAX and sequester it from mitochondria to prevent apoptosis (30), we examined if CTSG treatment resulted in BAX mitochondrial translocation. As shown in Figure 6, N–P, CTSG treatment led to BAX mitochondrial translocation in HCT116, DLD1, and RKO cells. Consistently, cytochrome c was released from the mitochondria to the cytosol (Figure 6, N–P and Supplemental Figure 7, H–J). Similarly, treating HCT116 cells with NET medium also induced BAX mitochondrial translocation (Supplemental Figure 7K), and this process could be suppressed by CTSGi (Supplemental Figure 7L). Together, our data suggest that CTSG enters CRC cells, cleaves 14-3-3ε, causes BAX mitochondrial translocation, and triggers apoptosis.

### NETs modulate the antitumor effort of the combination of CB-839 and 5-FU in PIK3CA-mutant mouse colon tumors in immune-competent mice.

**Figure 7:**
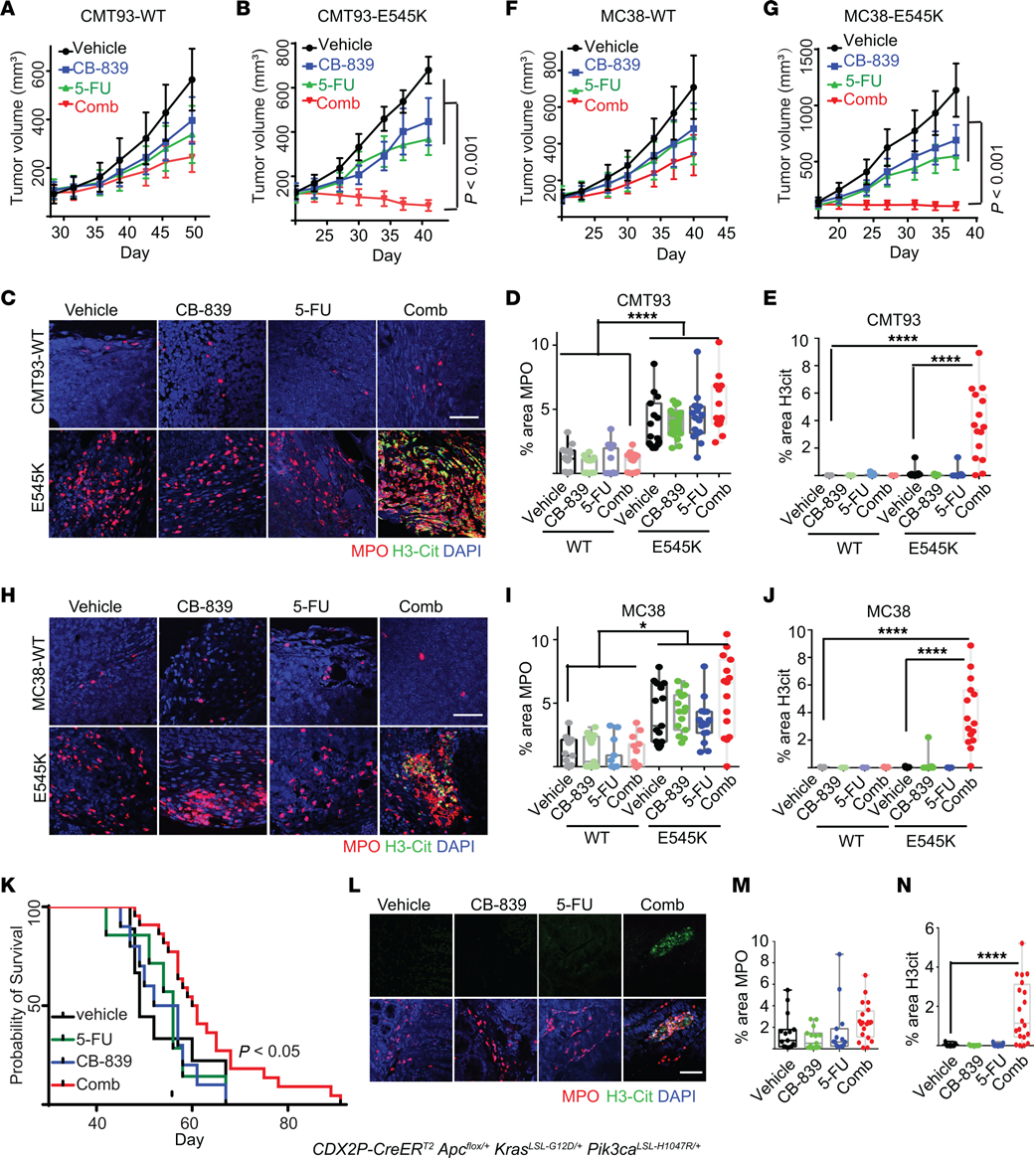
The combination of CB-839 and 5-FU induces NETs in syngeneic and GEM *PIK3CA*-mutant tumor models. (**A** and **B**) CMT93 *Pik3ca* WT or E545 K mutant tumors were treated with indicated drugs, with growth curves shown in **A** for CMT93 *Pik3ca* WT and (**B**) for CMT93 *Pik3ca* E545K mutant. (5 mice/group). (**C**–**E**) The indicated tumors were stained with antibodies against MPO and H3cit. Representative images are shown in **C**. Quantifications shown in **D** and **E** (*n* = 15/group). (**F** and **G**) MC38 *Pik3ca* WT or mutant tumors with indicated treatment, growth curve shown in **F** for MC38 *Pik3ca* WT, **G** for MC38 *PIK3CA* mutant (5 mice/group). (**H**–**J**) The indicated tumors were stained with antibodies against MPO and H3cit. Representative images are shown in **H**. Quantifications shown in **I** and **J** (*n* = 15/group). (**K**) *CDX2P-CreER^T2^ Apc*^fl/+^ *Kras*^LSL–G12D/+^ *Pik3ca*^LSL–H1047R/+^ mice were treated with tamoxifen and then treated with the indicated drug a week after tamoxifen treatment for 4 weeks. Kalplan-Meier curves of the mice are shown. A log-rank test was used to assess the statistical significance between the vehicle and the combination of CB-839 and 5-FU treatment groups. (**L–N**) *CDX2P-CreER^T2^ Apc*^fl/+^ *Kras*^LSL–G12D/+^ *Pik3ca*^LSL–H1047R/+^ mice were treated with tamoxifen, and 4 weeks later, the mice were treated with the indicated drugs, and colon tumors were harvested and stained with antibodies against MPO and H3cit (3 mice/group). Representative images are shown in **L**. Quantifications of MPO are shown in **M**. Quantifications of H3cit are shown in **N** (*n* = 15/group). 2-way ANOVA (**A**, **B,** **D**–**G**, **I** and **J**) or 1-way ANOVA (**N**) was used for statistical analysis. *P < 0.05; **P < 0.01; ***P < 0.001; ****P < 0.0001. Scale bar: 50 μm.

To test if the drug combination induced NETs in immune-competent mouse tumor models, we generated *Pik3ca* E545K oncogenic mutant knockin CMT93 and MC38 mouse colon cancer cell lines using CRISPR-mediated genome editing (Supplemental Figure 8, A and B). We then injected parental or *PIK3CA*-mutant cells subcutaneously into immune-competent C57/BL6J mice and treated them with the vehicle, CB-839, 5-FU, or the drug combination. As with human CRCs, the drug combination induced tumor regression and apoptosis of *PIK3CA* mutant, but not parental, CMT93 tumors (Figure 7, A and B and Supplemental Figure 8, C and D). Moreover, the combination of CB-839 and 5-FU induced NETs in *PIK3CA* mutant, but not parental, CMT93 tumors (Figure 7, C–E and Supplemental Figure 8, F and G). A single drug alone did not induce NETs in any of the tumors (Figure 7, C–E). Similar results were observed with *PIK3CA*-mutant MC38 mouse colon tumors (Figure 7, F– J and Supplemental Figure 8, E, H, and I). In contrast to the xenograft models with human CRC cell lines, the drug combination did not induce more tumor-infiltrating neutrophils compared with vehicle or single drugs (Figure 7, D and I). However, the *PIK3CA*-mutant tumors had more tumor-infiltrating neutrophils than tumors of the parental counterparts (Figure 7, C, D, H, and I).

*PIK3CA* mutations in human cancers occur in two hotspots: one in the helical domain (e.g., E545K) and the other in the kinase domain (H1047R). We have successfully constructed a *CDX2P-CreER^T2^ Apc*^fl/+^ *Kras*^LSL–G12D/+^ *Pik3ca*^LSL–H1047R/+^ mouse (GEM) model (31), in which an allele of Apc is inactivated, and Kras G12D and *Pik3ca* H1047R oncogenic mutations are activated by colon-specific and inducible CDX2P-CreER^T2^ transgene upon tamoxifen treatment. Mice were treated with vehicle, CB-839, 5-FU, or the drug combination for 3 weeks. As shown in Figure 7K, the drug combination, but not a single drug alone, substantially extended the survival of the mice. Consistently, the drug combination, but not a single drug, induced NETs (Figure 7, L–N and Supplemental Figure 8, J and K). As with the syngeneic models, the drug treatments did not induce more tumor-infiltrating neutrophils compared with vehicle treatment (Figure 7, L and M and Supplemental Figure 8J). Together, the data demonstrate that the combination of CB-839 and 5-FU induces NETs in *PIK3CA*-mutant tumors in syngeneic and GEM immune-competent mouse models.

**Figure 8:**
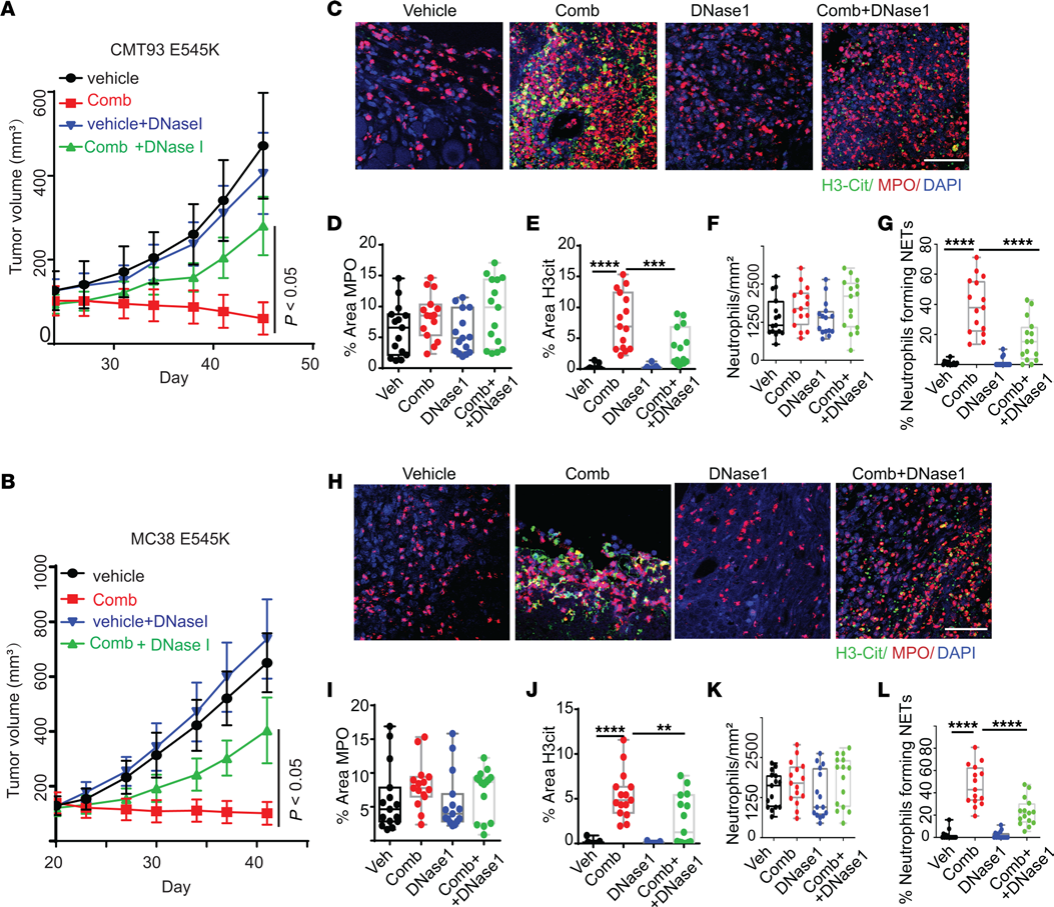
Disruption of NETs in syngeneic mouse tumors attenuates the tumor-inhibitory effect of the combination of CB-839 and 5-FU. The indicated syngeneic tumors in C57/BL6 mice were treated with vehicle (veh), or the drug combination (comb) with or without DNase I. Tumor growth curves are shown in **A** for CMT93 *Pik3ca* E545K mutant tumors and **B** for MC38 *Pik3ca* E545K mutant tumors (5 mice/group), respectively. Tumors were stained with antibodies against MPO and H3cit. Representative images are shown in **C** for CMT93 *Pik3ca* E545K tumors and **H** for MC38 *Pik3ca* E545K tumors, respectively. Quantifications are shown in **D**–**G** for CMT93 *Pik3ca* E545K tumors and **I**–**L** for MC38 *Pik3ca* E545K tumors, respectively (*n* = 15/group). Scale bar: 50 μm. 2-way ANOVA (**A** and **B)** or 1-way ANOVA (**D–G** and **I**–**L**) was used for statistical analysis. *P < 0.05; **P < 0.01; ***P < 0.001; ****P < 0.0001.

As with the xenograft models, DNase I treatment, which disrupted NET formation, attenuated the antitumor effect of the combination of CB-839 and 5-FU in CMT93 and MC38 *Pik3ca* E545K mutant syngeneic mouse models (Figure 8, A and B). Notably, DNase I treatment reduced the levels of NETs (Figure 8, E, G, J, and L), but did not impact the numbers of tumor-infiltrating neutrophil (Figure 8 C–L). Together, our data provide further evidence that NETs modulate the antitumor effect of the combination of CB-839 and 5-FU.

### CXCL5, a neutrophil chemokine, is upregulated in PIK3CA-mutant mouse colon cancer cells through the NF-κB pathway.

**Figure 9:**
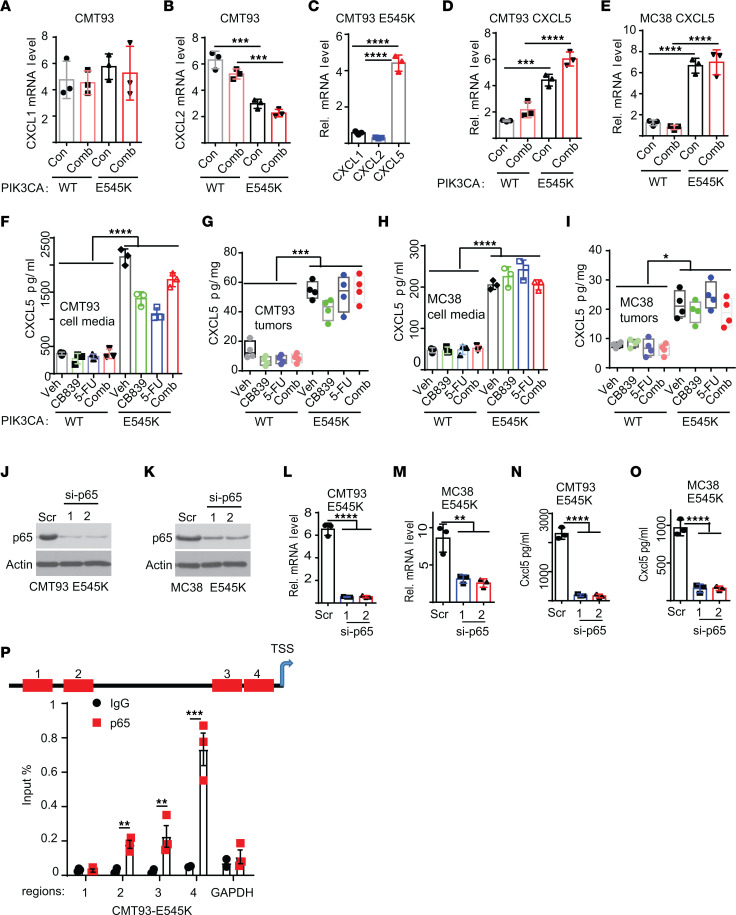
*Pik3ca* E545K mutation upregulates CXCL5 in mouse CRC cells. (**A** and **B**) RT-PCR of CXCL1 and CXCL2 levels in CMT93 *Pik3ca* WT and mutant cells with the indicated treatment overnight (*n* = 3/group). (**C**) RT-PCR of CXCL1, CXCL2, and CXCL5 in CMT93 E545K cells (*n* = 3/group). (**D** and **E**) RT-PCR of CXCL5 in *Pik3ca* WT and E545K cells. **D** for CMT93, **E** for MC38 (*n* = 3/group). (**F**–**I**) CXCL5 protein level in culture medium (**F** and **G**) and tumors (**H** and **I**) was measured by ELISA (*n* = 3/group for **F** and **H**, *n* = 4/group for **G** and **I**). (**J**–**O**) p65 was knocked down by 2 independent siRNA. Cell lysates were blotted with the indicated antibodies (**J** and **K**). CXCL5 mRNA levels were measured by qRT-PCR (**L** and **M**). Secreted CXCL5 was measured by ELISA (**N** and **O**) (*n* = 3/group). (**P**) CMT93 *PIK3CA* mutant cells were treated with the combination of CB-839 and 5-FU. ChIP-PCRs were performed (*n* = 3/group). 2 tailed *t* test for **P**, 2-way ANOVA (**B** and **D**–**I**) or 1-way ANOVA (**C** and **L**–**O**) was used for statistical analysis. *P < 0.05; **P < 0.01; ***P < 0.001; ****P < 0.0001.

Because the mouse does not have an IL-8 homolog (32), we next investigated what chemokine(s) attracted neutrophils to tumors in the mouse syngeneic models. Given that CXCL1, CXCL2, and CXCL5 are the known neutrophil chemokines in mice (33), we measured mRNA expression of these chemokines in CMT93 parent and *Pik3ca* E545K knockin (KI) cells treated with vehicle or the combination of CB-839 and 5-FU. As shown in Figure 9, A–C, CXCL5 expression levels were upregulated in *PIK3CA*-mutant CMT93 cells compared with the parental counterparts, regardless of the drug treatment. In contrast, there was no difference in CXCL1 levels among the different conditions (Figure 9A), whereas CXCL2 mRNA levels decreased in *Pik3ca* E545K-KI CMT93 cells compared with the parental cells (Figure 9B). It is worth noting that CXCL1 and CXCL2 were expressed at much lower levels than CXCL5 (Figure 9C). Similarly, CXCL5 upregulated in MC38 *Pik3ca* E545K-KI cells compared with parental cells (Figure 9, D and E). Thus, we focused on CXCL5 for in-depth studies. ELISA analyses showed that secreted CXCL5 proteins were upregulated in *PIK3CA-*mutant CMT93 and MC38 tissue cultured cells and syngeneic tumors (Figure 9, F–I). Notably, CXCL5 is not regulated by mutant *PIK3CA*/p110α in human CRC cells (Supplemental Figure 3G). We postulate that the promoters of CXCL5 in mice and humans are different and therefore have different responses to *PIK3CA*/p110α mutation.

We next set out to identify the mechanisms by which the *Pik3ca* mutation upregulates CXCL5. It was reported that NF-κB regulates CXCL5 transcriptionally (34) and that *PIK3CA* mutations activate the NF-κB pathway (35). We thus knocked down p65 with 2 independent siRNA in *Pik3ca*-E545K–mutant CMT93 and MC38 cells. Figure 9, J–O show that the knockdown of p65 resulted in decreased levels of CXCL5 mRNA and secreted proteins. ChIP-qPCR analyses showed that p65 bound to the promoter regions of CXCL5 in *Pik3ca*-E545K mutant CMT93 cells (Figure 9P). Together, the data suggest that *Pik3ca* mutation activates the NF-κB pathway to upregulate CXCL5 in mouse CRCs, thereby attracting more tumor-infiltrating neutrophils.

### CTSG plays a key role in antitumor effect of the combination of CB-839 and 5-FU in syngeneic models.

**Figure 10:**
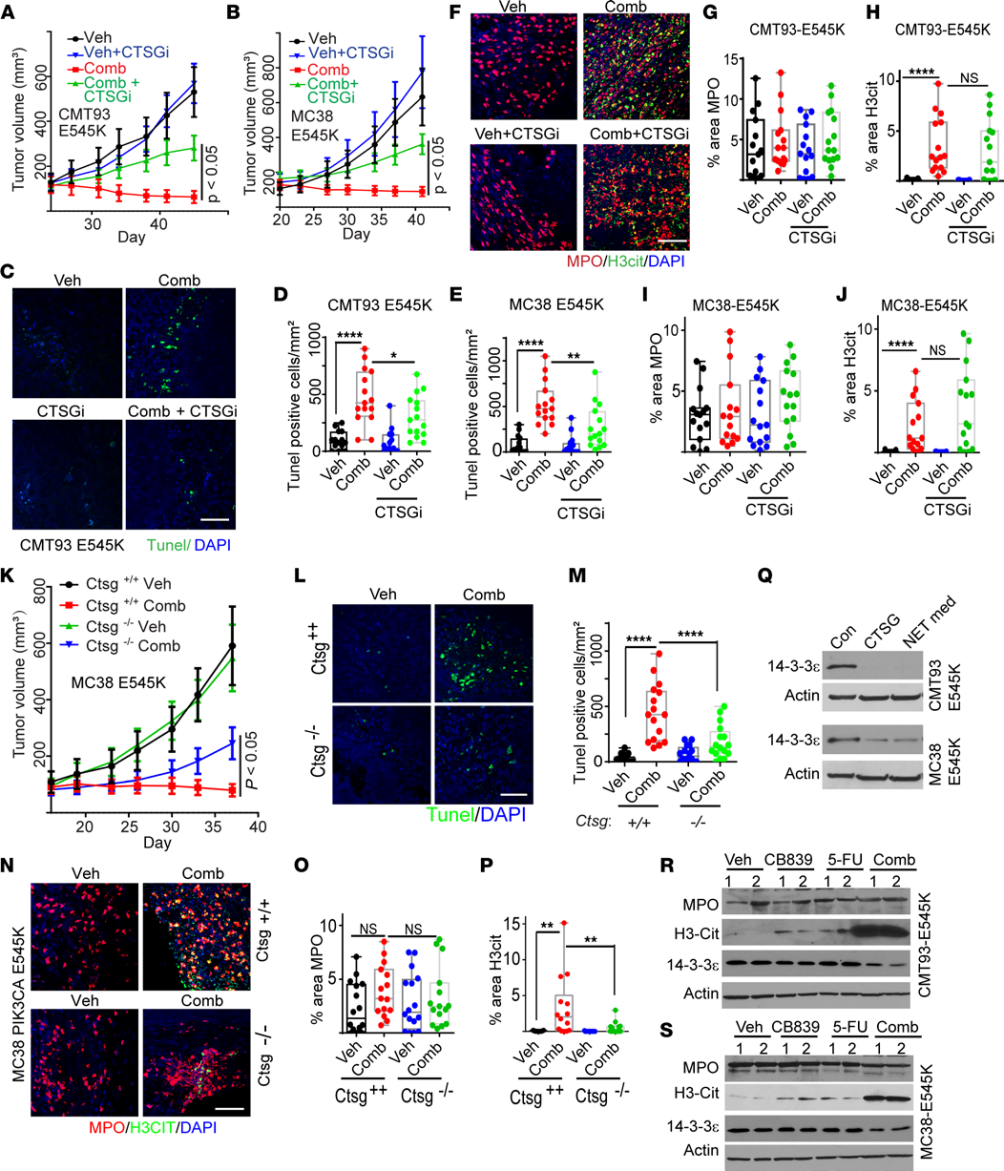
CTSG in NETs induces apoptosis in the syngeneic model. (**A** and **B**) The indicated syngeneic tumors were treated with vehicle or the drug combination with or without CTSGi (5 mice/group). Growth curves are shown in **A** and **B**. (**C**–**E**) Tunel staining was performed on the syngeneic tumors from **A** and **B**. Representative images of CMT93 tumors are shown in **C**, and quantifications are shown in **D** and **E** (*n* = 15/group), (**F**–**J**) Tumors from **A** and **B** were stained with antibodies against MPO and H3cit (**F**) and quantified (**G**–**J**). (**K**–**M**) MC38 *Pik3ca* E545K mutant cells were injected into C57/BL6 mice of the indicated genotypes and treated with vehicle or the combination of CB-839 and 5-FU (5 mice/group). The growth curves are shown in **K**. Tunel staining was performed on the syngeneic tumors; representative images are shown in **L**, and quantifications are shown in **M**. (**N**–**P**) The tumors shown in **K** were stained with antibodies against MPO and H3cit. Representative images are shown in **N**. Quantifications are shown in **O** and **P** (*n* = 15/group). (**Q**) mouse CRC cells treated with CTSG and NET medium for 16 hours, cell lysates were harvested and blotted with indicated antibodies. (**R** and **S**) tumors shown in **A** and **B** were blotted with indicated antibodies. 2-way ANOVA (**A**, **B**, **K**, **M**, and **P**) or 1-way ANOVA (**D**, **E**, **H**, and **J**) was used for statistical analysis. *P < 0.05; **P < 0.01; ***P < 0.001; ****P < 0.0001.

Given that we have demonstrated in xenograft models that CTSG in the NETs plays a key role in killing cancer cells, we set to determine whether the same is true for the syngeneic models. We first tested if CTSGi could attenuate the antitumor effect of the combination treatment. As shown in Figure 10, A–E and Supplemental Figure 9, A and B, CTSGi treatment indeed attenuated the therapeutic effect and apoptosis induced by the drug combination in both CMT93 and MC38 *Pik3ca*-E545K–mutant tumor models. As with the xenograft models, CTSGi treatment did not perturb NET formation (Figure 10, F–J and Supplemental Figure 9, C and D).

To test rigorously if CTSG is required for the tumor inhibitory effect of the combination of CB-839 and 5-FU, we obtained a *Ctsg* KO C57/BL6J mouse strain. Western blot analyses validated the KO of CTSG (Supplemental Figure 9E). We then injected MC38 *Pik3ca*-E545K–mutant cells into *Ctsg^–/–^* mice and their *Ctsg^+/+^* littermates. Once tumors reached an average size of 100 mm^3^, mice were treated with vehicle or a combination of CB-839 and 5-FU. As shown in Figure 10, K–M, the tumor inhibitory effect and apoptosis induced by the drug combination was attenuated in *Ctsg*^/–^ mice compared with their WT littermates. Surprisingly, although the number of tumor-infiltrating neutrophils was not decreased in *Ctsg*^–/–^ mice (Figure 10, N and O and Supplemental Figure 9F), the levels of NETs induced by the drug combination were substantially reduced in syngeneic tumors in *Ctsg*^–/–^ mice compared with the *Ctsg*^+/+^ littermates (Figure 10P and Supplemental Figure 9G). We then isolated neutrophils from *Ctsg*^+/+^ and *Ctsg*^–/–^ mice and treated them with vehicle or the combination of CB-839 and 5-FU. Supplemental Figure 9, H and I show that the drug combination failed to induce NETs in vitro, suggesting that CTSG is involved in NET induction in neutrophils by the drug combination. Notably, a previous study suggested that CTSG was involved in NET formation as well (36). Consistently, the conditioned medium of neutrophils from *Ctsg*^–/–^ mice treated with CB-839 and 5-FU reduced apoptosis induction in CRC cells (Supplemental Figure 9J). It is worth noting that the CTSGi that we used in the experiments described in Figure 5, H–J and Figure 10, A–E is not cell permeable. Thus, it only inhibits CTSG in the NETs, not CTSG inside neutrophils, thereby not preventing NETs induction, as shown in Supplemental Figure 5, N–T and Figure 10, H and J. Together, our results suggest that CTSG plays a critical role in modulating the tumor-inhibitory effect of the drug combination.

Lastly, we have shown that CTSG enters human CRC cells to cleave 14-3-3ε to trigger apoptosis. We set out to test if the same mechanism occurred in mouse colon cancer cells. As shown in Figure 10Q, treating CMT93 and MC38-*PIK3CA*–mutant cells with either recombinant CTSG or NET-conditioned medium resulted in dramatically reduced levels of 14-3-3ε. As with the xenograft models, the combination of CB-839 and 5-FU decreased the levels of 14-3-3ε in the syngeneic CMT93 and MC38 *Pik3ca* E545K mutant tumor models (Figure 10, R and S).

### A phase II clinical trial of a combination of CB-839 with capecitabine, an oral prodrug of 5-FU, in patients with metastatic PIK3CA-mutant CRC.

**Figure 11:**
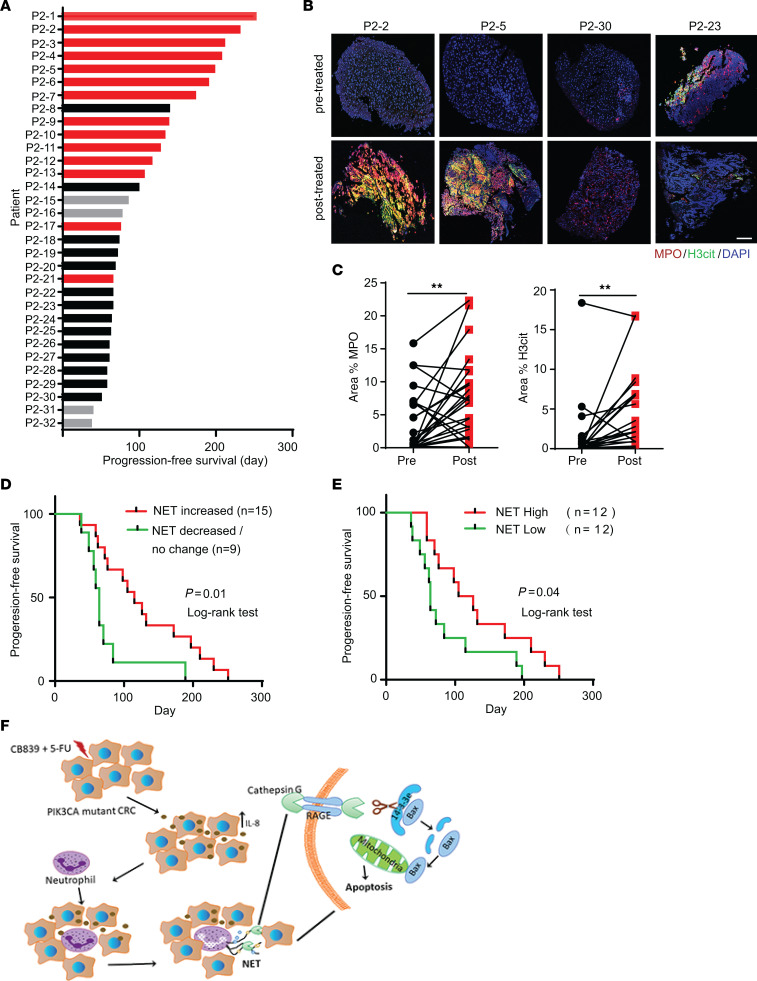
Higher tumor NET levels are associated with longer PFS. (**A**) PFS of 32 metastatic *PIK3CA* mutant CRC patients in a phase II clinical trial of the combination of CB-839 and capecitabine, an oral prodrug of 5-FU. Red bars, stable disease; black bars, progressive disease; and gray bars, not evaluable. (**B** and **C**) Tumor biopsies were stained with anti-MPO and anti-H3cit antibodies. Representative images are shown in **B**, and quantifications are shown in **C**. ** *P* < 0.01; paired Student’s *t* test, 2-tailed. Scale bar: 100 μM. (**D**) Kaplan-Meier curves of PFS of patients are plotted with increased levels of H3cit in posttreatment biopsies compared with pretreatment biopsies versus decreased levels of H3cit levels in posttreatment biopsies compared with pretreatment biopsies. (**E**) Kaplan-Meier plot of PFS of patients whose posttreatment biopsies with high levels of H3cit versus those with low levels of H3cit. (**F**) A model of CB-839 plus 5-FU-induced NETs, which release CTSG, enter cancer cells, cleave 14-3-3ε, lead to BAX mitochondrial translocation, and trigger apoptosis.

We previously conducted a phase I clinical trial of the combination of CB-839 and capecitabine in patients with advanced solid tumors and showed that the drug combination was well tolerated (10). The recommended phase II dose (RP2D) was CB-839 800 mg by mouth twice daily continuously and capecitabine 1,000 mg/m^2^ orally twice daily on days 1–14 of a 21-day treatment cycle (10). In this trial, we observed approximately 40% of patients with *PIK3CA*-mutated metastatic CRC had over 6 months of PFS. Here, we conducted a phase II clinical trial on patients with metastatic *PIK3CA*-mutant CRC who were refractory to prior fluoropyrimidine-based chemotherapy. The primary objective was assessing PFS greater than 6 months. Thirty-two eligible patients, whose ages ranged from 37 to 81 years old, with a median age of 56, were treated with the drug combination. The baseline patient characteristics are shown in Supplemental Table 2, and the PFS of these patients are shown in Figure 11A. The median PFS of the patients is 75 days (range 36 to 251 days), and 7 patients had over 6-month PFS (21.8%). Of 28 patients who were evaluable for response, 14 patients (50%) had stable disease, and 14 had progressive disease as the best response. Although no objective response was observed, 5 patients had tumor regression (range 1% to 15% reduction of overall tumor burden). The combination was again adequately tolerated (Supplemental Table 3), with a toxicity profile similar to what we observed in the patients in the phase I clinical trial (10).

### Increased levels of NETs in posttreatment tumor biopsies are associated with longer PFS.

Of the 32 patients, we obtained pre- and posttreatment biopsy pairs in 24 of them. We stained the 24 biopsy pairs with antibodies against MPO and H3cit. Compared with pretreatment counterparts, MPO levels were increased in the posttreatment biopsies of 17 patients, decreased in 5 patients, and were not changed in 2 patients (Figure 11, B and C). The levels of H3cit were increased in the posttreatment biopsies in 15 patients, decreased in 1 patient, and not changed in 8 patients (Figure 11, B and C). In 14 patients, both MPO and H3cit levels increased in posttreatment biopsies, suggesting that the combinational treatment of CB-839 and capecitabine led to increased tumor-infiltrating neutrophils and induction of NETs. In one patient, the levels of H3cit increased in posttreatment biopsy without a change in MPO levels. Interestingly, patients who had increased levels of H3cit, marking NETs, in posttreatment biopsies over pretreatment biopsies were associated with long PFS (Figure 11D). Moreover, when taking account H3cit levels only in posttreatment biopsies, higher levels of H3cit were also associated with longer PFS (Figure 11E). However, the increased levels of tumor-infiltrating neutrophils (MPO positive cells) were not associated with long PFS (Supplemental Figure 10B).

## Discussion

We reveal here a previously unrecognized mechanism by which chemotherapy inhibits tumor growth. Our studies demonstrated that the combination of CB-839 and 5-FU induces NETs to inhibit in vivo growth of tumors with a *PIK3CA* mutation in xenograft, syngeneic, and GEM models. Furthermore, disruption of NETs by DNase I treatment or depletion of neutrophils attenuates the tumor-inhibitory effect of the drug combination. In the phase II clinical trial, the drug combination induces NETs in tumors in most patients, and the increased levels of NETs are associated with longer PFS. NETs were first identified as a mechanism by which neutrophils trap and kill bacteria (2). Follow-up studies show that NETs also kill fungi and parasites. Recent studies also demonstrate that NETs cause tissue damage in a variety of pathologic conditions, including autoimmune diseases (37), thrombosis (38), liver injury (39), and toxic epidermal necrolysis (40). However, to the best of our knowledge, it has not yet been reported that induction of NETs in vivo by any anticancer drugs inhibits tumor growth. Moreover, in addition to the combination of CB-839 and 5-FU, our data showed that 6 other chemotherapy drugs could also induce NETs in vitro, suggesting that induction of NETs may be an unappreciated mechanism by which some chemotherapies inhibit tumor growth. It is worth noting that recent studies demonstrated that spontaneous NETs, which occur at low levels, promote tumor metastasis by extracellular matrix remodeling (5). In contrast, the combination of CB-839 and 5-FU induces a large number of NETs that kill cancer cells through the action of protease cathepsin G. Nonetheless, it has been shown that NETs play a role in radiotherapy resistance (41). Moreover, a recent study showed that chemotherapy induces cancer cells to release IL-1β to promote NET formation, which, in turn, activates latent TGFβ secreted by cancer cells and causes chemoresistance (42).

Mechanistically, we demonstrated here that the combination of CB-839 and 5-FU induced IL-8 expression in *PIK3CA*-mutant CRC cells, thereby attracting neutrophils into the tumor tissues. On the other hand, the drug combination acts on neutrophils to induce NETs and release CTSG. CTSG enters cancer cells through cell surface protein RAGE and cleaves 14-3-3ε, which leads to BAX mitochondrial translocation and triggers apoptosis of cancer cells (Figure 11F). Here, we provide several pieces of evidence to support such mechanisms: (a) knockout of *IL-8* in CRC cells attenuated the tumor inhibitory effect of the combination of CB-839 and 5-FU; (b) a noncell-permeable CTSG inhibitor, which does not perturb NET induction, attenuated the tumor inhibitory effect of the drug combination; (c) the drug combination was less effective on syngeneic *PIK3CA*-mutant tumors in *Ctsg*-KO mice compared with their WT littermates, although the drug combination induced fewer NETs in tumors in the *Ctsg*-KO mice, suggesting that CTSG may be involved NET induction process in neutrophils; (d) KO of *RAGE* attenuated the tumor inhibitory effort of the drug combination; (e) CTSG treatment reduced 14-3-3ε levels in cultured CRC cells and cleaved recombinant 14-3-3ε in vitro; (f) the combination of CB-839 and 5-FU reduced 14-3-3ε levels in xenograft and syngeneic tumors; and (g) CTSG treatment induced BAX mitochondrial translocation in cultured CRC cells. As mentioned above, NETs have been shown to be involved in many pathogenic conditions that cause tissue damage. However, the detailed mechanisms by which NETs damage tissues in these conditions remain elusive. We postulated that the mechanisms we uncovered here might be applicable to other pathogenic conditions, warranting further investigation.

The phase II clinical trial of CB-839 and capecitabine in patients with *PIK3CA*-mutated colon cancer reported here had 21.8% of patients surpassing 6 months PFS, which did not meet its prespecified objective of 25% of patients with over 6 months PFS. Although 5 patients had reduced overall tumor burden, no patient had a reduction greater than 30%. Nonetheless, the currently demonstrated immune mechanism could inform future clinical trial designs. Potential future studies should ensure adequate circulating neutrophil counts throughout treatment cycles as diminished neutrophils abrogate the effectiveness of the combination in animal models (Supplemental Figure 1E). We note that G-CSF is a clinically available agent that is typically administered to reduce infection risk in patients receiving myeloablative or highly myelosuppressive chemotherapy regimens (43), but not to patients receiving capecitabine. Thus, these findings may now serve as a basis for future trials in which the use of G-CSF is tested in combination with agents such as CB-839 and capecitabine, to ensure that the potential antitumor benefit of neutrophils is maximized during the treatment course. In support, patients who had longitudinally increased neutrophil numbers after drug treatment had longer PFS than those who had decreased neutrophil numbers after the drug treatment in the phase II clinical trial (Supplemental Figure 10C). Notably, these data are different from the tumor-infiltrating neutrophils on biopsies from days 10–14, which represented a short-term snapshot and had no predictive value for PFS (Supplemental Figure 10B). We postulate that the long-term effect of the drug treatment on neutrophils and NET formation in tumors may have better predictive value. More generally, these findings may serve as a basis for preclinical and clinical exploration of a potential therapeutic advantage for maximizing neutrophils in therapies with other traditional cytotoxic anticancer drugs. Moreover, we showed here that increased NET levels after drug treatment are associated with longer PFS (Figure 11, D and E). Given that a recent study showed that PD-L1 modulates NET induction (44), further studies combining NET-inducing treatments such as this with checkpoint inhibition to harness adaptive immune mechanisms may also be warranted.

## Methods

All reagents used in this study are listed in Supplemental Table 4. Additional methods are listed in Supplemental Methods.

### Mice

#### Sex as a biological variable.

Our examined both male and female animals. Similar findings are reported in both sexes.

#### Mouse experiments.

For HCT116, DLD1, and RKO human colon cancer cell lines, 2 × 10^6^ cells were injected subcutaneously and bilaterally into 6-week-old nude or NSG mice,as described previously (45). For CMT93 and MC38 cells, 5 × 10^6^ of WT or *PIK3CA* E545K mutant knock-in cells were injected subcutaneously and bilaterally into the flanks of 6-week-old C57BL/6J mice (Jackson Labs). Once tumors reached an average volume of 150 mm^3^, mice were randomly divided into different treatment groups. Mice were treated daily with vehicle control, CB839 (200 mg/kg, oral gavage, provided by Calithera Biosciences), 5-FU (30 mg/kg, i.p.), or the drug combination. For NE inhibitor treatment, mice were treated daily with vehicle control or sivelestat (10 mg/kg) by intraperitoneal injection. Tumor volume was measured with calipers, and volumes were calculated as length × width^2^/2.

For the CDX2P-CreER^T2^ *Apc*^fl/+^ *Kras*^LSL–G12D/+^ *Pik3ca*^LSL–H1047R/+^ mouse (GEM) model, 8-week-old mice were injected intraperitoneally with tamoxifen (100 mg/kg, dissolved in corn oil) for 3 consecutive days. For survival analysis, mice were randomly assigned into 4 treatment groups a week after tamoxifen injection and treated with vehicle control, CB839, 5-FU, or the drug combination for 4 weeks. For netosis staining, mice were treated with the drugs for 27 days after the tamoxifen injection for a week. Colon tumor tissues will be fixed and stained with netosis markers for further analysis.

### Phase II clinical trial design

Both male and female patients were enrolled for the studies. This clinical trial was conducted with the approval of the institutional review board and according to good clinical practice with a primary objective of determining the rate of 6-month PFS of the combination of oral CB-839 when administered with oral capecitabine. The secondary objectives are to determine the response rate and correlative studies. Pretreatment and on-treatment (10–14 days after initial treatment) liver tumor biopsies were taken by CT-guided needle biopsy. Response to therapy was assessed per RECIST 1.1 utilizing CT imaging obtained every 9 weeks. Patients were permitted to continue treatment until disease progression or development of unacceptable toxicity. All patients provided written informed consent prior to participating in the study. The trial was registered on clinicaltrials.gov (NCT02861300).

#### Eligibility.

Patients were eligible for study entry if they had a CRC, had progressed on all standard lines of therapy, and progressed on prior fluoropyrimidine-based chemotherapy. Patients otherwise must have been at least 18 years of age, had an ECOG performance status of 0 or 1, had normal bone marrow, renal, and hepatic function, had the ability to swallow pills, and be able to understand and have the willingness to sign consent. Patients were not eligible if they had ongoing treatment-related toxicities that were greater than grade 1, and they could not be receiving other investigational agents. Central nervous system involvement by their cancer or prior allergic reaction to either CB-839 or capecitabine was not permitted.

### Statistics

GraphPad Prism software was used to create the graphs. Data are plotted as mean ± SEM. For 2 group comparison, we applied the2-tailed *t* test to compare the means between 2 groups, assuming unequal variances. For comparisons of more than 2 groups with a single variable, 1-way ANOVA was used to assess for any significant differences across all groups, together with Dunnett’s multiple pairwise comparison test to compare each pair for statistically significant difference between the pair. If the data had more than 1 variable, 2-way ANOVA and Tukey’s multiple pairwise comparison tests were used. For xenograft growth, we carried out 2-way ANOVA for repeated measurements to test whether there was an overall difference in the tumor size by testing group differences, or whether there was a difference in the development of tumor size over time between the 2 groups by testing the interaction between time and group. Kaplan-Meier analysis was used to assess differences in PFS stratified by level changes of various biomarkers, generating a log-rank *P* value as well as median survival time with 95% CI. Patient response to therapy during the phase II clinical trial was defined per RECIST criteria. Patients were considered evaluable for response if they had measurable disease at the time of study entry, had received at least 1 cycle of therapy and had undergone a repeat disease evaluation with imaging. PFS was defined as the time from the beginning of treatment to RECIST evidence of progressive disease as determined by radiography or by clinical progression. *P* < 0.05 is defined as statistically significant.

### Study approval

Animal experiments were approved by the Case Western Reserve University Animal Care and Use Committee. Both male and female mice were used in this study. The clinical trial was conducted with the approval of the Institutional Review Board and according to good clinical practice.

### Data availability

The RNA-Seq were deposited into GEO (accession #: GSE245839). All other data are reported in the Supporting Data Value file.

## Author contributions

ZW conceived the project. ZW, YL, YZ, JES, SCCH, AYH, RC, and JEW designed the bench experiments. DB and JE designed the phase II clinical trial. DB and AAK led the clinical trials at Seidman Cancer Center and Taussig Cancer Center, respectively. YL, YZ, DJ, JES, GM, XZ, YW,and ST performed the bench experiments. YL, SW, TD, YW, GD,S Krishnamurthi, JEW, DB, and ZW analyzed data. JES, RTL, BE, MS, S Kamath, AM, S Krishnamurthi, AK, and DB recruited patients for the clinical trial. ZW, YL, DB, and RC wrote the manuscript.

## Supplementary Material

Supplemental data

Unedited blot and gel images

Supporting data values

## Version 1 01/09/2024

In-Press Preview

## Version 2 03/01/2024

Electronic publication

## References

[1] Burn GL (2021). The neutrophil. Immunity.

[2] Brinkmann V (2004). Neutrophil extracellular traps kill bacteria. Science.

[3] Papayannopoulos V (2018). Neutrophil extracellular traps in immunity and disease. Nat Rev Immunol.

[4] Korkmaz B (2010). Neutrophil elastase, proteinase 3, and cathepsin G as therapeutic targets in human diseases. Pharmacol Rev.

[5] Albrengues J (2018). Neutrophil extracellular traps produced during inflammation awaken dormant cancer cells in mice. Science.

[6] Adrover JM (2023). NETworking with cancer: the bidirectional interplay between cancer and neutrophil extracellular traps. Cancer Cell.

[7] Vasan N, Cantley LC (2022). At a crossroads: how to translate the roles of PI3K in oncogenic and metabolic signalling into improvements in cancer therapy. Nat Rev Clin Oncol.

[8] Samuels Y (2004). High frequency of mutations of the PIK3CA gene in human cancers. Science.

[9] Hao Y (2016). Oncogenic PIK3CA mutations reprogram glutamine metabolism in colorectal cancer. Nat Commun.

[10] Zhao Y (2020). 5-fluorouracil enhances the antitumor activity of the glutaminase inhibitor CB-839 against *PIK3CA*-mutant colorectal cancers. Cancer Res.

[11] Liu K (2019). BCG-induced formation of neutrophil extracellular traps play an important role in bladder cancer treatment. Clin Immunol.

[12] Schedel F (2020). Evidence and impact of neutrophil extracellular traps in malignant melanoma. Pigment Cell Melanoma Res.

[13] Wang CL (2023). DNase I and sivelestat ameliorate experimental hindlimb ischemia-reperfusion injury by eliminating neutrophil extracellular traps. J Inflamm Res.

[14] Hao Y (2022). Nuclear translocation of p85β promotes tumorigenesis of PIK3CA helical domain mutant cancer. Nat Commun.

[15] Rot A (1991). Chemotactic potency of recombinant human neutrophil attractant/activation protein-1 (interleukin-8) for polymorphonuclear leukocytes of different species. Cytokine.

[16] Harada A (1994). Cloning of a cDNA encoding a mouse homolog of the interleukin-8 receptor. Gene.

[17] Kucharzik T (2005). Acute induction of human IL-8 production by intestinal epithelium triggers neutrophil infiltration without mucosal injury. Gut.

[18] Fan X (2007). Murine CXCR1 is a functional receptor for GCP-2/CXCL6 and interleukin-8/CXCL8. J Biol Chem.

[19] Cacalano G (1994). Neutrophil and B cell expansion in mice that lack the murine IL-8 receptor homolog. Science.

[20] Mihara K (2005). Human CXCR2 (hCXCR2) takes over functionalities of its murine homolog in hCXCR2 knockin mice. Eur J Immunol.

[21] Li Y (2023). PD-L1 expression is regulated by ATP-binding of the ERBB3 pseudokinase domain. Genes Dis.

[22] Zhang X (2005). Activation of the Nrf2/antioxidant response pathway increases IL-8 expression. Eur J Immunol.

[23] An Z (2019). Neutrophil extracellular traps induced by IL-8 aggravate atherosclerosis via activation NF-κB signaling in macrophages. Cell Cycle.

[24] Remijsen Q (2011). Neutrophil extracellular trap cell death requires both autophagy and superoxide generation. Cell Res.

[25] Branzk N, Papayannopoulos V (2013). Molecular mechanisms regulating NETosis in infection and disease. Semin Immunopathol.

[26] Greco MN (2002). Nonpeptide inhibitors of cathepsin G: optimization of a novel beta-ketophosphonic acid lead by structure-based drug design. J Am Chem Soc.

[27] El Rayes T (2015). Lung inflammation promotes metastasis through neutrophil protease-mediated degradation of Tsp-1. Proc Natl Acad Sci U S A.

[28] Yang L (2020). DNA of neutrophil extracellular traps promotes cancer metastasis via CCDC25. Nature.

[29] Sionov RV (2019). Neutrophil cathepsin G and tumor cell RAGE facilitate neutrophil anti-tumor cytotoxicity. Oncoimmunology.

[30] Nomura M (2003). 14-3-3 Interacts directly with and negatively regulates pro-apoptotic Bax. J Biol Chem.

[31] Zhao Y (2019). Colorectal cancers utilize glutamine as an anaplerotic substrate of the TCA cycle in vivo. Sci Rep.

[32] Singer M, Sansonetti PJ (2004). IL-8 is a key chemokine regulating neutrophil recruitment in a new mouse model of Shigella-induced colitis. J Immunol.

[33] Rajarathnam K (2019). How do chemokines navigate neutrophils to the target site: Dissecting the structural mechanisms and signaling pathways. Cell Signal.

[34] Jia X (2021). CXCL5/NF-κB pathway as a therapeutic target in hepatocellular carcinoma treatment. J Oncol.

[35] Hutti JE (2012). Oncogenic PI3K mutations lead to NF-κB-dependent cytokine expression following growth factor deprivation. Cancer Res.

[36] Park J (2016). Cancer cells induce metastasis-supporting neutrophil extracellular DNA traps. Sci Transl Med.

[37] Gupta S, Kaplan MJ (2016). The role of neutrophils and NETosis in autoimmune and renal diseases. Nat Rev Nephrol.

[38] Carminita E (2021). DNAse-dependent, NET-independent pathway of thrombus formation in vivo. Proc Natl Acad Sci U S A.

[39] Huang H (2015). Damage-associated molecular pattern-activated neutrophil extracellular trap exacerbates sterile inflammatory liver injury. Hepatology.

[40] Kinoshita M (2021). Neutrophils initiate and exacerbate Stevens-Johnson syndrome and toxic epidermal necrolysis. Sci Transl Med.

[41] Shinde-Jadhav S (2021). Role of neutrophil extracellular traps in radiation resistance of invasive bladder cancer. Nat Commun.

[42] Mousset A (2023). Neutrophil extracellular traps formed during chemotherapy confer treatment resistance via TGF-β activation. Cancer Cell.

[43] Mehta HM, Corey SJ (2021). G-CSF, the guardian of granulopoiesis. Semin Immunol.

[44] Zhu CL (2022). PD-L1 maintains neutrophil extracellular traps release by inhibiting neutrophil autophagy in endotoxin-induced lung injury. Front Immunol.

[45] Zhao Y (2010). Identification and functional characterization of paxillin as a target of protein tyrosine phosphatase receptor T. Proc Natl Acad Sci U S A.

