# Supplementary material for: Neutrophil extracellular traps induced by chemotherapy inhibit tumor growth in murine models of colorectal cancer
Source: J Clin Invest. 2024 Jan 9;134(5):e175031. doi: 10.1172/JCI175031 (PMC10904055; doi:10.1172/JCI175031)
Supplement: Supplemental data [file jci-134-175031-s095.pdf]

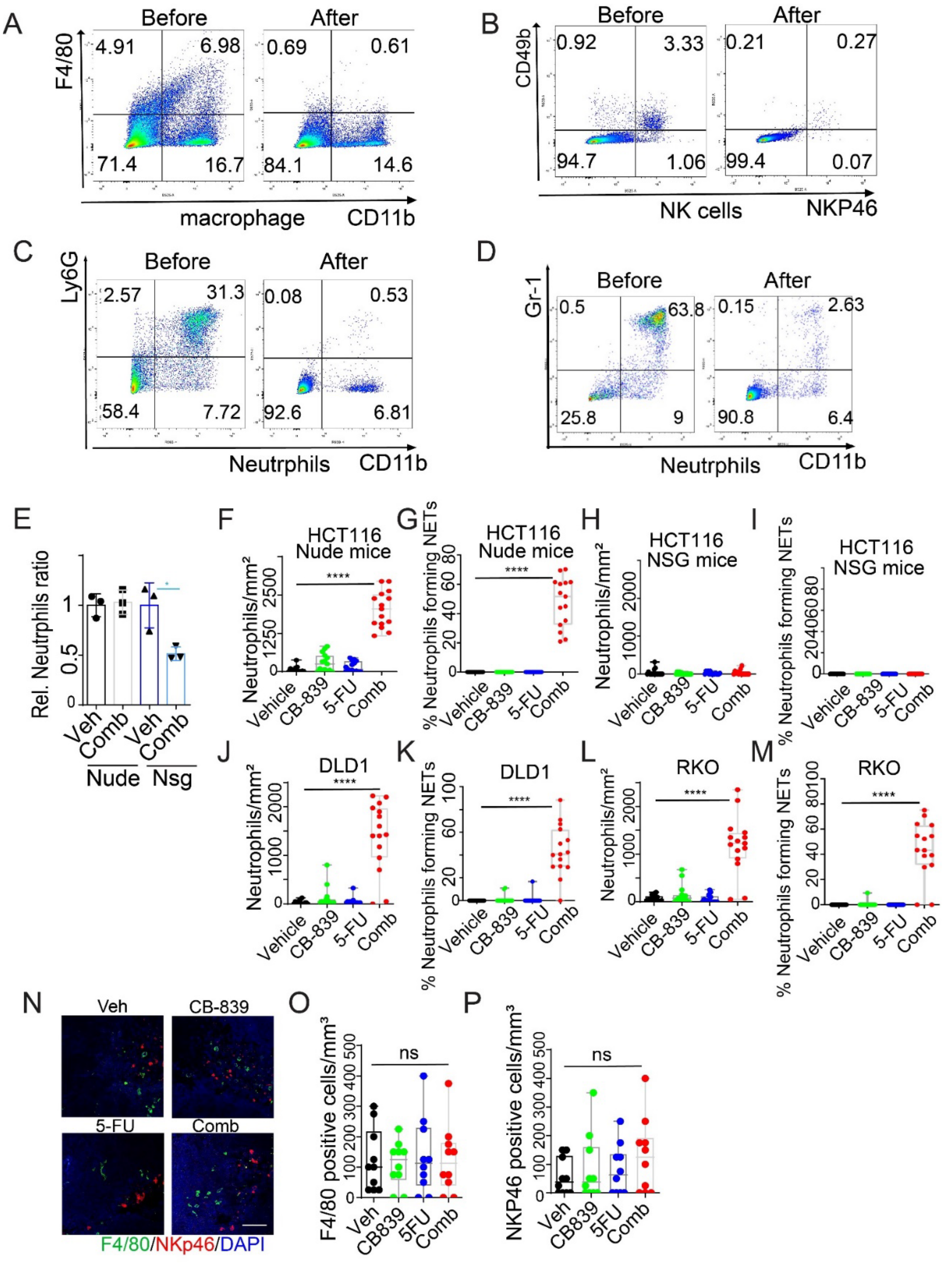

Figure S1. The combination of CB-839 and 5-FU induces NETs. (**A**) FACS analyses of macrophage depletion in nude mice for the experiment results shown in Fig. 1C. (**B**) FACS analyses of NK cell depletion in nude mice for the experiment results shown in Fig. 1D. (**C & D**) FACS analyses of neutrophil depletion in nude mice for the experiment results shown in Fig. 1E. (**E**) Neutrophil count in peripheral blood after indicated treatment for 7 days in nude mice and NSG mice. The ratios of neutrophils vs. leukocytes are plotted (n=3/group). (**F to M**) Quantifications of NETs using a formula: number of NET-forming neutrophils/number of neutrophils. NETs are marked by H3-Cit and MPO, and neutrophils are marked by MPO only. (**N to P**) tumors from Fig.1A stained with anti-F4/80 and NKP46 antibodies. Representative images are shown in (N). Quantification data are shown in O and P (n=10/group). Scale bar: 50 µm. One-way ANOVA (E to M, O & P) was used for statistical analysis. *p<0.05; **p<0.01; ***p<0.001; ****p<0.0001.

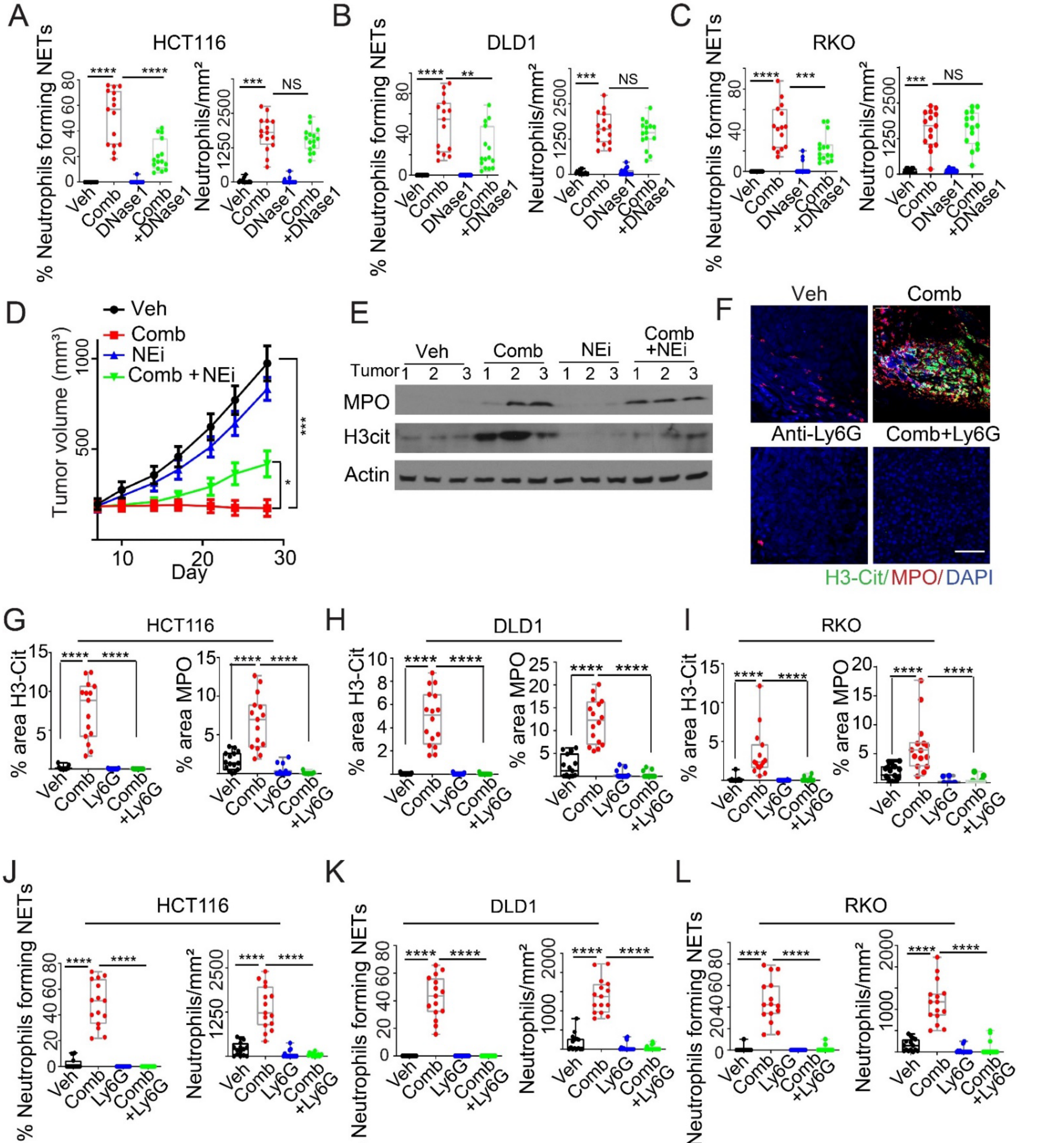

Figure S2. Disruption of NETs in xenograft tumors attenuates the tumor-inhibitory effect of the combination of CB-839 and 5-FU. (**A** to **C**) Tumors from Figures 2A, 2D, and 2E were stained with anti-MPO and anti-H3cit antibodies. Quantifications of the percentage of neutrophil forming NETs and neutrophils number per field are shown in (A) for HCT116, (B) for DLD1, and (C) for RKO (n=15/group). (**D & E**) HCT116 xenograft tumors were treated with vehicle or drug combination with or without sivelestat (10 mg/kg). Tumor growth curves are shown in (D). Western blots of tumors from (D) with indicated antibodies (E). (**F** to **L**) Tumors from Fig.1E to G were stained with antibodies against MPO and H3cit. Representative images are shown in (F) for HCT116 tumors. Quantifications are shown in G and J for HCT116, H and K for DLD1, and I and L for RKO (n=15/group). Scale bar: 50 µm. ANOVA was used for statistical analysis, as shown in (D). Two-way ANOVA (D) or one-way ANOVA (A to C, G to L) was used for statistical analysis. *$p<0.05$; **$p<0.01$; ***$p<0.001$; ****$p<0.0001$.

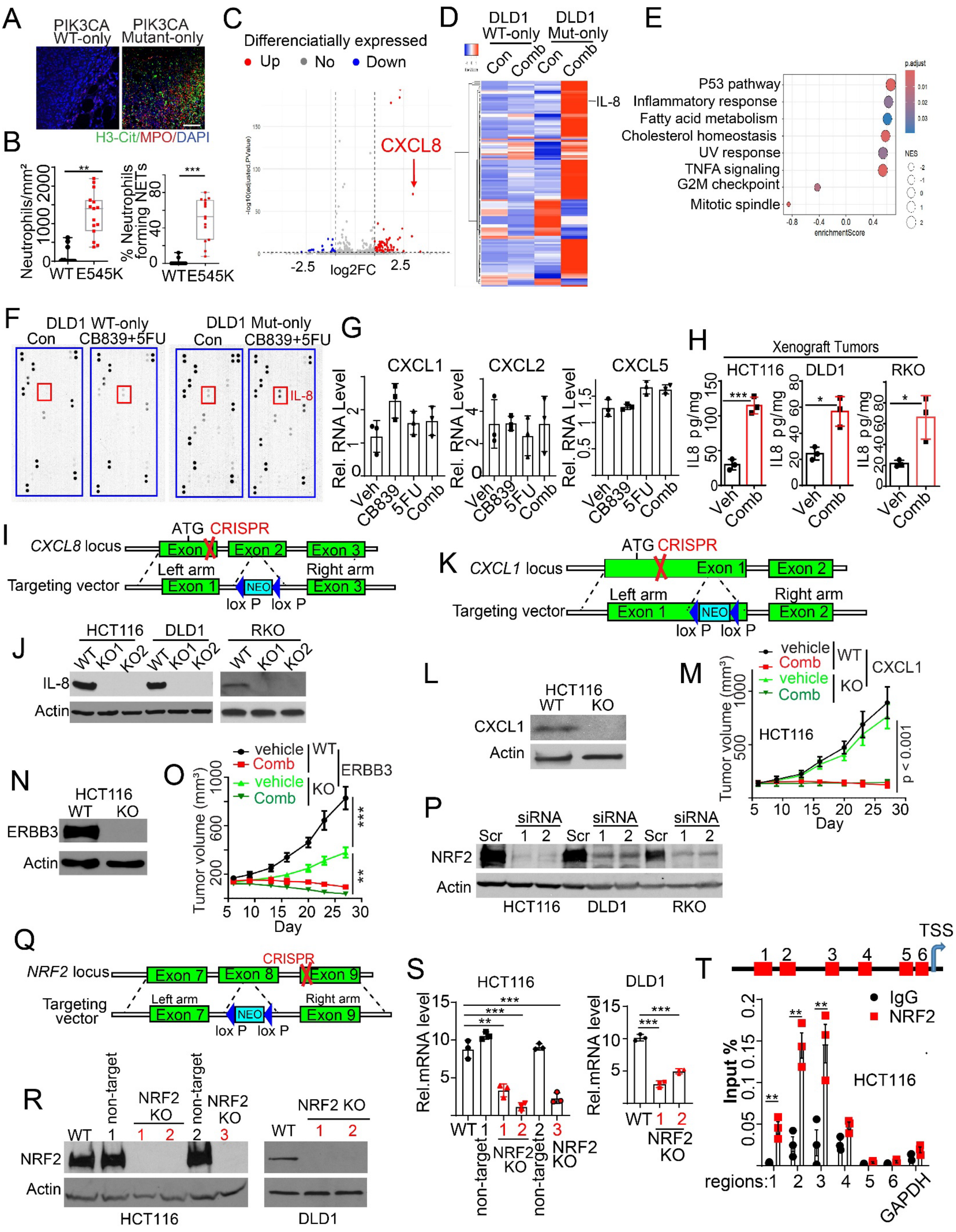
A
PIK3CA WT-only
PIK3CA Mutant-only
H3-Cit/MPO/DAPI
B
Neutrophils/mm²
WT E545K
% Neutrophils forming NETs
C
Differenciatially expressed
Up No Down
CXCL8
log2FC
D
DLD1 WT-only
DLD1 Mut-only
Con Comb Con Comb
IL-8
E
P53 pathway
Inflammatory response
Fatty acid metabolism
Cholesterol homeostasis
UV response
TNFA signaling
G2M checkpoint
Mitotic spindle
enrichmentScore
p.adjust
NES
F
DLD1 WT-only
DLD1 Mut-only
Con CB839+5FU
IL-8
G
CXCL1 CXCL2 CXCL5
Rel. RNA Level
Veh CB839 5FU Comb
H
Xenograft Tumors
HCT116 DLD1 RKO
IL8 pg/mg
Veh Comb
I
CXCL8 locus
ATG CRISPR
Exon 1 Exon 2 Exon 3
Left arm Right arm
Targeting vector
NEO
lox P lox P
J
HCT116 DLD1 RKO
WT KO1 KO2
IL-8
Actin
K
CXCL1 locus
ATG CRISPR
Exon 1 Exon 2
Left arm Right arm
Targeting vector
NEO
lox P lox P
L
HCT116
WT KO
CXCL1
Actin
M
vehicle Comb WT
vehicle Comb KO
CXCL1
HCT116
Tumor volume (mm³)
p < 0.001
Day
N
HCT116
WT KO
ERBB3
Actin
O
vehicle Comb WT
vehicle Comb KO
ERBB3
Tumor volume (mm³)
Day
P
Scr siRNA 1 2
NRF2
Actin
HCT116 DLD1 RKO
Q
NRF2 locus
CRISPR
Exon 7 Exon 8 Exon 9
Left arm Right arm
Targeting vector
NEO
lox P lox P
R
WT non-target 1 NRF2 KO 1 2 non-target 2 NRF2 KO 3
NRF2 KO WT 1 2
NRF2
Actin
HCT116 DLD1
S
HCT116 DLD1
Rel.mRNA level
WT 1 non-target 1 2 NRF2 KO 2 non-target 3 NRF2 KO
WT 1 2 NRF2 KO
T
TSS
1 2 3 4 5 6
IgG NRF2
Input %
HCT116
regions: 1 2 3 4 5 6 GAPDH

Figure S3. The combination of CB-389 and 5-FU induces IL-8 expression in human CRC cells by activating the transcriptional activity of NRF2. (**A & B**) Isogenic PIK3CA mutant-only and WT-only xenograft tumors in nude mice were treated with the combination of CB-839 and 5-FU, as described in (1). Tumors were stained with antibodies against MPO and H3cit. Representative images are shown in (A), and quantifications are shown in (B) (n=15/group). (**C&D**) Isogeneic PIK3CA WT-only and mutant-only cells were DMSO or the combination of CB-839 and 5-FU. RNAs were extracted for RNA-seq analyses. Deferentially expressed genes in PIK3CA mutant-only cells vs WT-only are plotted as volcano plot (C) and heatmap (D) (n=3/group). (**E**) Top ten enriched pathways in the RNA-seq data. The inflammatory response pathway includes IL-8. (**F**) Cytokine array analyses of isogenic PIK3CA WT-only and mutant-only cells. (**G**) qRT-PCR of CXCL1, CXCL2, and CXCL5 in HCT116 treated with the indicated drug (n=3/group). (**H**) ELISA assays were used to measure IL-8 levels in the lysates of the indicated tumors. (**I**) Schematics of IL-8 knockout in human colon cancer cells using CRISPR genome editing. (**J**) Western blots analyses of parental and IL-8 knockout cells. The indicated cells were treated with Brefeldin A for 5 hours. Cells and media were harvested for Western blot analyses. (**K**) Schematics of CXCL1 knockout in HCT116 cells using CRISPR genome editing. (**L)** Western blots analyses of parental and CXCL1 knockout cells. (**M**) HCT116 CXCL1WT and CXCL1 knockout tumors treated with vehicle or drug combination. The growth curves are shown (5 mice/group). (**N** & **O**) Western blots analyses of ERBB3 protein in WT and knockout cells (N). HCT116 ERBB3 WT and ERBB3 knockout tumors were treated with vehicle or drug combination. The growth curves are shown in (O), 5 mice/group. (**P**) Western blot analyses of NRF2 in cells transfected with scramble (Scr) or two independent siRNAs against NRF2. (**Q** to **S**) Schematics of NRF2 knockout in HCT116 cells using CRISPR genome editing (Q). Western blots analyses of NRF2 protein in WT and knockout cells (R), and qRT-PCR of IL-8 levels in NRF2 WT and knockout cells (S) (n=3/group). (**T**) NRF2 binds to promoter regions of IL-8. HCT116 cells were treated with a combination of CB-839 and 5-FU. ChIP-PCRs were performed. Scale bar: 50 µm. ANOVA was used for statistical analysis, as shown in (O) and (M). A two-tailed t-test was used for statistical analyses for (B, H & T), and one-way ANOVA (S) or two-way ANOVA (M & O) was used for statistical analysis. *$p<0.05$; **$p<0.01$; ***$p<0.001$; ****$p<0.0001$.

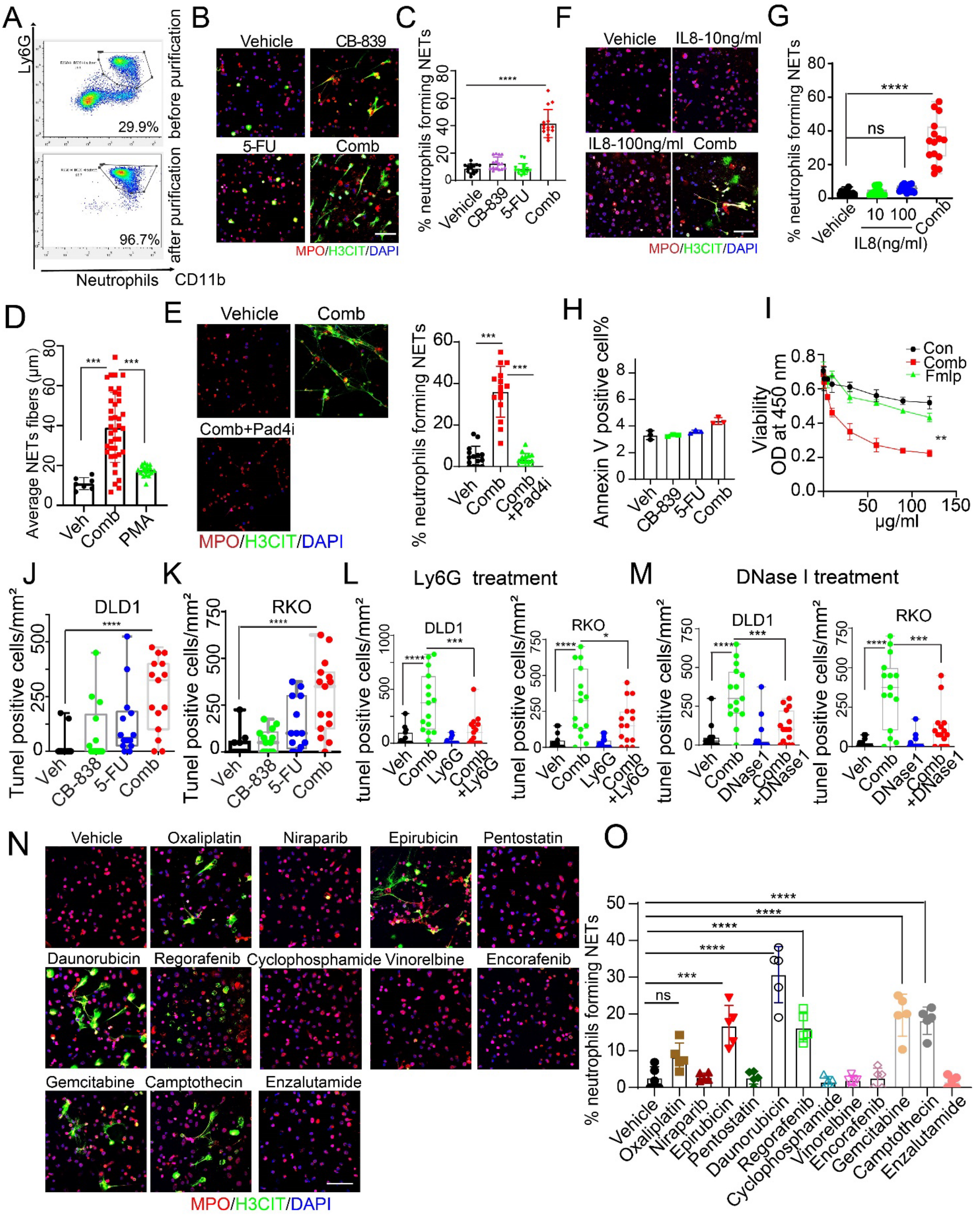

Figure S4. **Chemotherapies induce NETs**. (**A**) FACS analysis to assess the purity of neutrophils. (**B** & **C**) Purified neutrophils were treated with the indicated drugs (1 μM each) and stained with antibodies against MPO and H3cit. Representative images are shown in (B), and quantifications of the H3cit signal are shown in (C)

(n=15/group). (**D)** The lengths of NETs fibers shown in Figure 4A were quantified. **(E**) Purified neutrophils were treated with a drug combination (1 μM each) with or without PAD4 inhibitor GSK484 (2 μM) and stained with antibodies against MPO and H3cit. Representative images are shown in the left panel, and quantifications of the H3cit signal are shown in the right panel. (**F & G)** Purified neutrophils were treated with the drug combination (1 μM each) or IL-8 and stained with antibodies against MPO and H3cit. Representative images are shown in (F), and quantifications of the H3cit signal are shown in (G). (**H**) Annexin V analyses of HCT116 cells after indicated drugs (1 μM each) for 16 hours. (**I)** Equal numbers of HCT116 cells treated with various dilutions of NET conditioned medium [Neutrophils treated with the combination of CB-839 and 5-FU (1 μM each)], control conditioned medium (neutrophils without drug treatment) or conditioned medium of activated neutrophils (treated with 1 μM fMLP, a neutrophil activator). Cell numbers were counted by CCK8 kit. (**J & K**) Tunel staining was performed on DLD1 and RKO tumors shown in Fig. 1 M & O. Quantifications are shown in (J) for DLD1 and (K) for RKO. (**L & M**) Tunel staining was performed on DLD1 and RKO tumors shown in Fig. 1 F & G, Fig. 2D & E. Quantifications are shown in (L) and (M) (n=15/group). (**N&O**) Purified neutrophils were treated with the indicated drugs (1 μM each, except 5 μM for oxaliplatin) and stained with antibodies against MPO and H3cit. Representative images are shown in (N), and quantifications of the H3cit signal are shown in (O) (n=5/group). Scale bar: 50 μm. One-way ANOVA (C & D, E & G, J to M, O) or two-way ANOVA (I) was used for statistical analysis. *p<0.05; **p<0.01; ***p<0.001; ****p<0.0001.

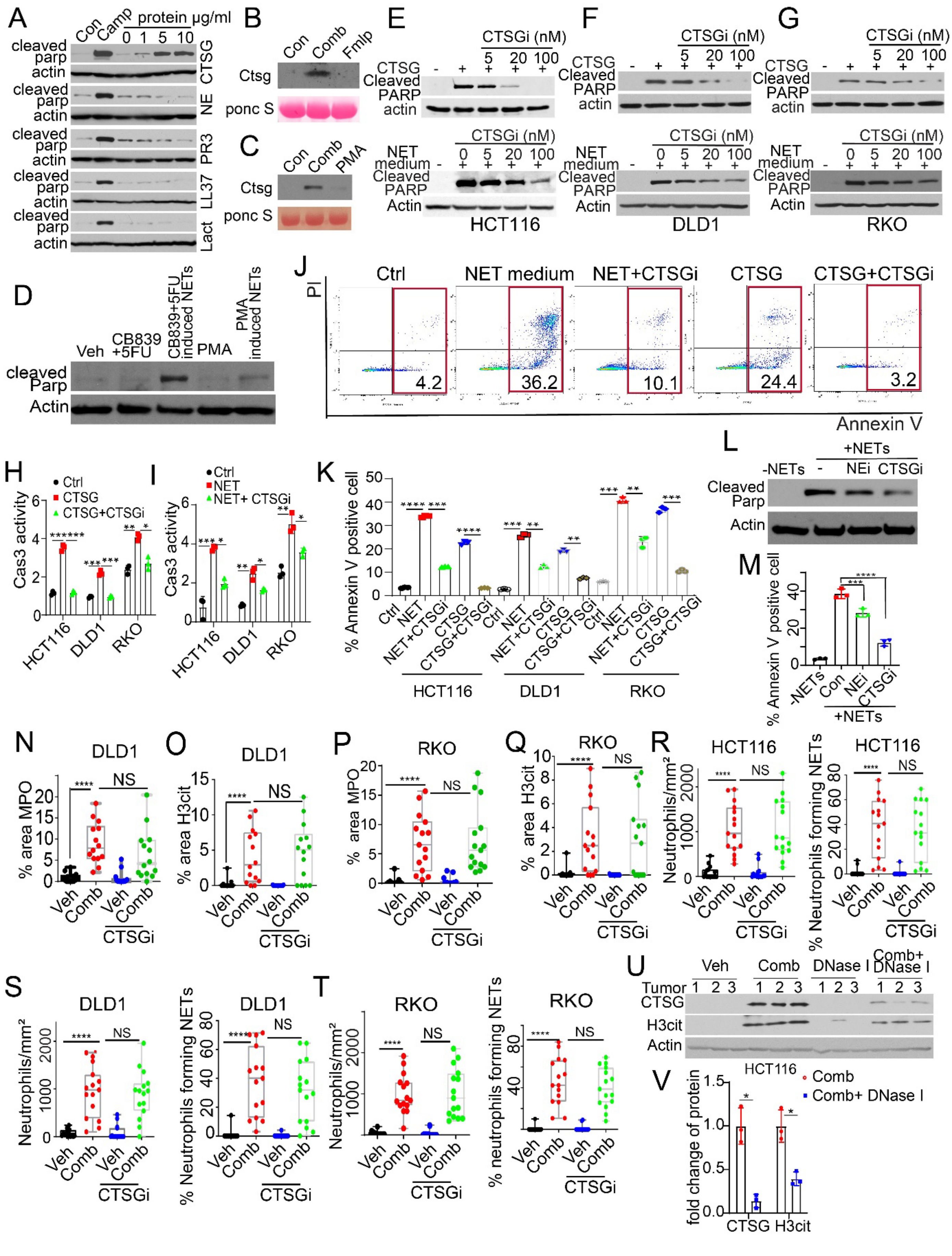

Figure S5. Cathepsin G (CTSG) in NETs induces apoptosis in tumors. (**A**) HCT116 CRC cells were treated with the indicated recombinant proteins. HCT116 cells were treated with camptothecin (3 µM) as a positive

control. Cell lysates were blotted with the indicated antibodies. (**B & C**) Purified neutrophils were treated with the combination of CB-839 and 5-FU (1 μM each), fMLP (1 μM) or PMA (1 μM) overnight. Tissue culture media were blotted with an anti-CTSG antibody. (D) HCT116 cells were treated with the indicated conditional medium for 16 hours, and cell lysates were blotted with indicated antibodies. (**E to K**) The indicated CRC cells were treated with recombinant CTSG or NET medium, with or without CTSGi. Cell lysates were blotted with the indicated antibodies (E to G). Cells were treated with either 5 μg/ml CTSG or 30 μg/ml NET-conditioned medium for 16 hours. Caspase 3 activities were measured (H and I) (n=3/group). Annexin V-positive cells were countered by flow cytometry (J and K) (n=3/group). (**L & M**) HCT116 CRC cells were pre-incubated with or without 0.1 μM indicated protease inhibitors before being treated with 30 μg/ml NET-conditioned medium overnight. Cell lysates were blotted with indicated antibodies in (L). Cells were harvested for Annexin V analyses (M). (**N to T**). Tumors shown in Figures 5H, 5I, and 5J were stained with anti-MPO and anti-H3cit and quantified. (**U & V**) Lysates of tumors shown in Figure 2A were blotted with the indicated antibody (U), and quantifications of CTSG and H3cit are shown in (V) (n=15/group). One-way ANOVA analyses in (H to K, M to T), two-tailed t-test statistical analyses in (V) *p<0.05; **p<0.01; ***p<0.001; ****p<0.0001.

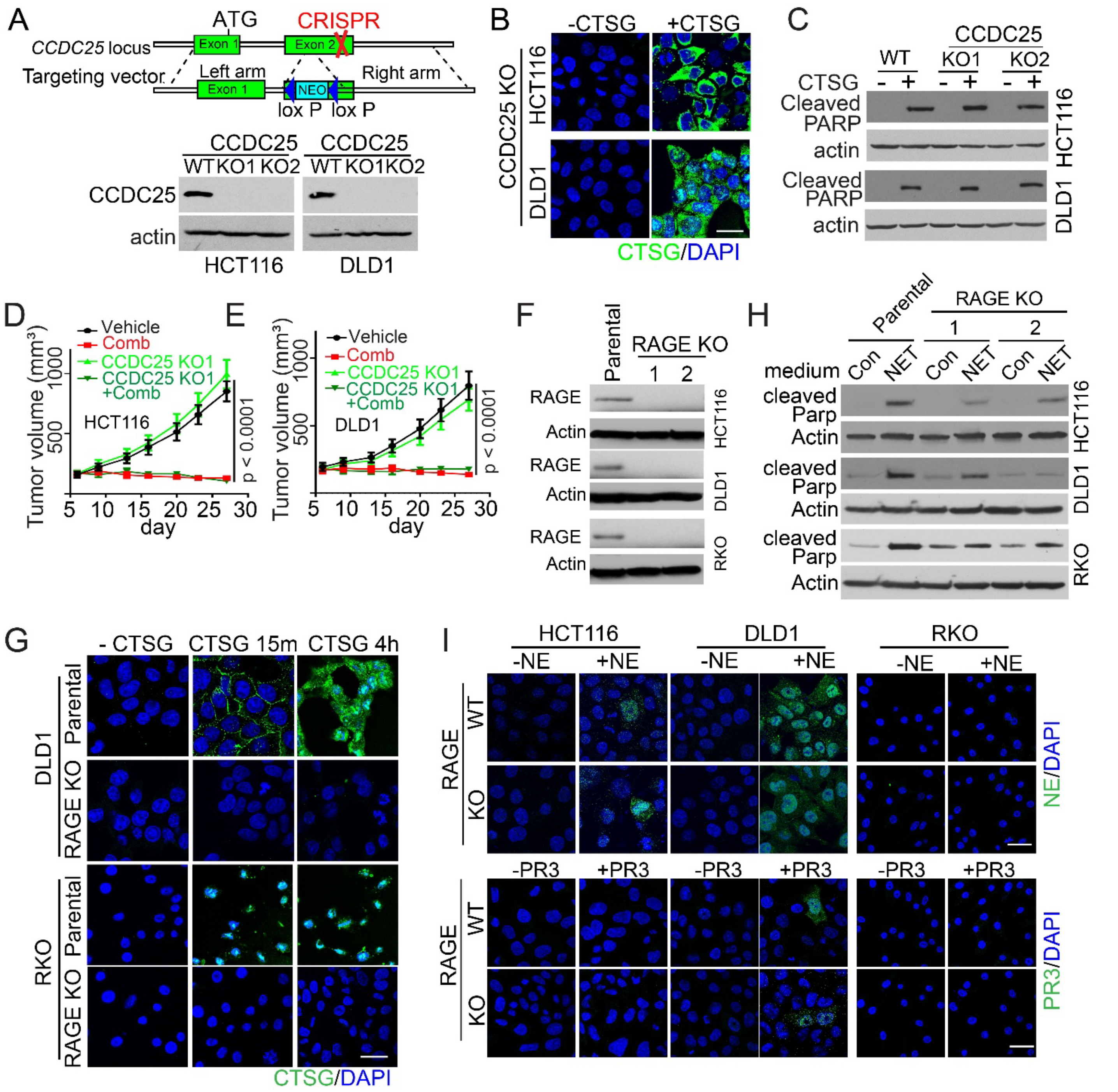

Figure S6. CTSG enters cancer cells through RAGE to cleave 14-3-3ε. (**A** to **E**) CCDC25 knockout clones were generated in HCT116 and DLD1 CRC cells using CRISPR/cas9 mediated genome editing (A). (B) The indicated cells were treated with or without CTSG for 4 hr and stained with an anti-CTSG antibody. (C) CCDC25 WT and knockout CRC cells were treated with or without CTSG (5 µg/ml) for 16 hours. Cell lysates were blotted with indicated antibodies. (D) CCDC25 WT and knockout xenograft tumors were treated with vehicle or drug combination for 3 weeks, with growth curves shown in D (HCT116) and E (DLD1) (5 mice/group). (**F**) RAGE knockout clones were generated in HCT116, DLD1, and RKO CRC cells using CRISPR/cas9 mediated genome editing. (**G**) The indicated cells were treated with or without CTSG for 15 min or 4 hr and stained with an anti-CTSG antibody. (**H**) RAGE WT and knockout CRC cells treated with or without conditional medium for 16 hours. Cell lysates were blotted with the indicated antibodies. (**I**) HCT116, DLD1, and RKO cells were incubated with 3 µg/ml NE or PR3 for 4 hours, followed by staining with indicated antibodies. Scale bar: 50 µm. Two-way ANOVA was used for statistical analysis, as shown in (D), and (E). ***p<0.001.

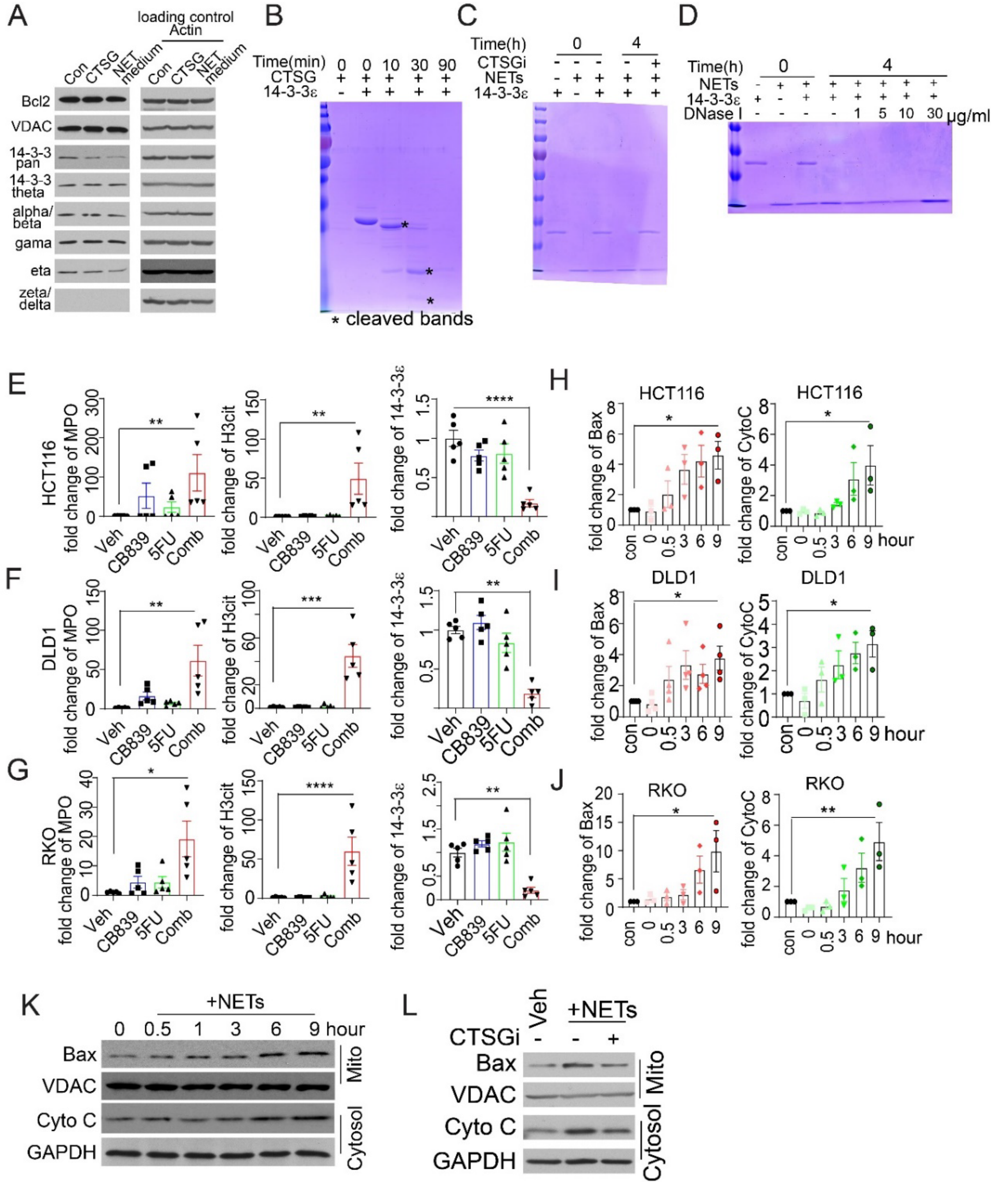

Figure S7. CTSG cleaved 14-3-3 ε to trigger apoptosis in CRC cells. (**A**). HCT116 cells were incubated with recombinant CTSG or NET medium for 16 hours. Cell lysates were blotted with the indicated antibodies. (**B**) Recombinant 14-3-3ε (1 μg) and CTSG proteins (100 ng) were incubated for the indicated time, and the reaction mixtures were run on an SDS-PAGE gel. (**C & D**) Recombinant 14-3-3ε and NET medium incubated with or without CTSGi or DNAse 1 for 4 hours. Reaction mixtures were run on an SDS-PAGE gel. (**E to G**) quantifications of MPO, H3cit, and 14-3-3ε in the tumors shown in Figure 6 G to 6 I (n=5 each group). (**H** to **J**) Quantifications of Bax and Cyto C in human CRCs after CTSG treatment (n=3). (**K & L**) HCT116 cells were treated with NET medium for indicated hours. Mitochondrial and cytosolic parts were extracted by a cell fractionation kit and blotted with indicated antibodies. One-way ANOVA was used for statistical analyses for (E to J). *p<0.05; **p<0,01; ***p<0.001.

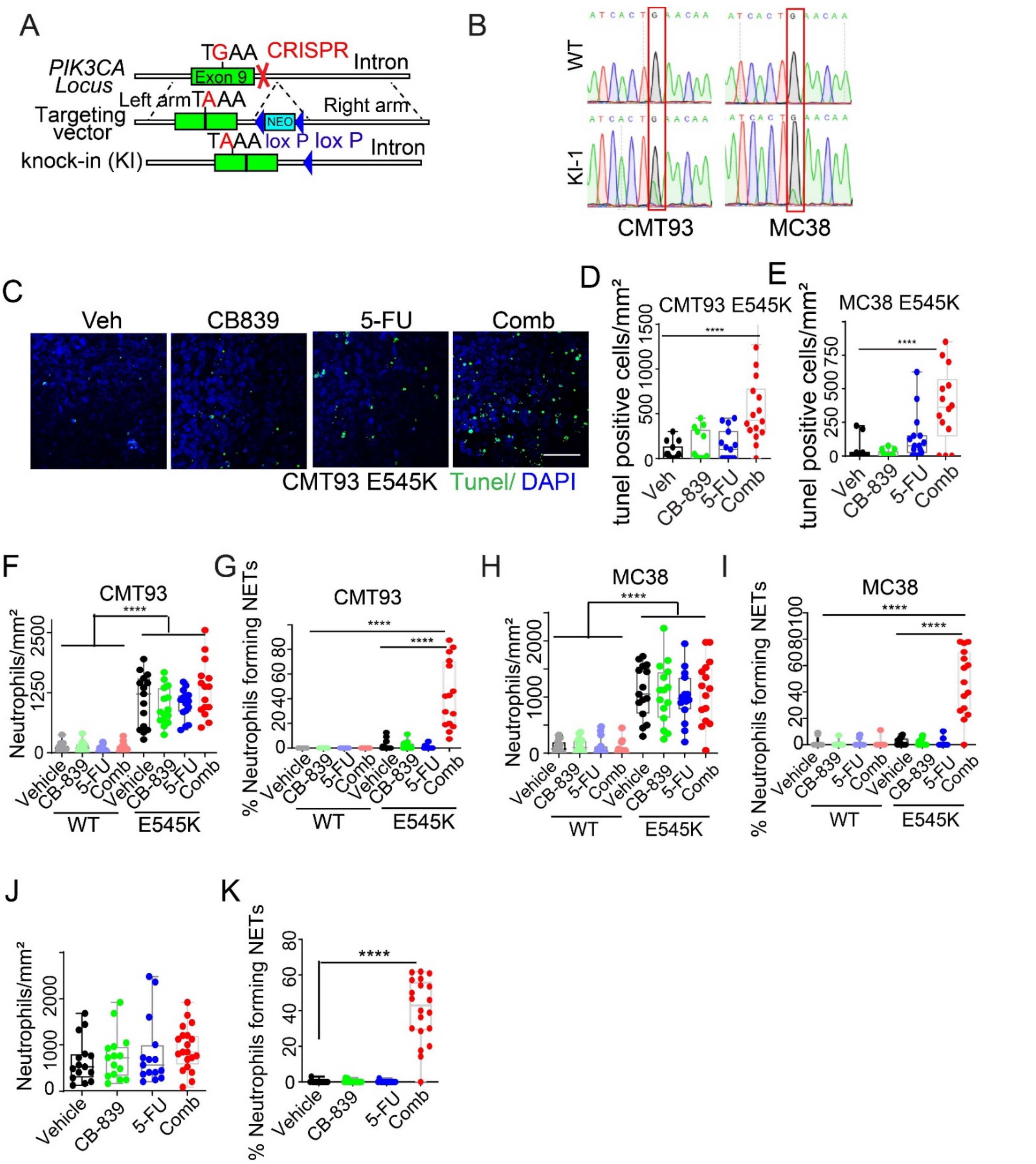

Figure S8. The combination of CB-839 and 5-FU induces NETs in syngeneic and genetically engineered mouse (GEM) PIK3CA mutant tumor models. (**A**) Schematics of knockin (KI) of PIK3CA E545K oncogenic mutation in CMT93 and MC38 mouse colon cancer cells using CRISPR genome editing. (**B**) Genomic sequencing of parental and PIK3CA E545K mutant CMT93 and MC38 mouse colon cancer cells. Red rectangular indicates the mutation site. (**C** to **E**) Tumors shown in Figures 7A & B were performed with Tunel staining. Representative images are shown in (C). Quantifications shown in D & E (n=15/group). (F to K) Quantifications using the formula: area of MPO/total area*100 (F), area of H3-cit/total area*100 (G), numbers of MPO positive cells per mm^2(H), or (number of NET-forming neutrophils/number of neutrophils)*100. One-way ANOVA (D, E & K) or two-way ANOVA (F to I) was used for statistical analysis. *p<0.05; **p<0.01; ***p<0.001; ****p<0.0001.

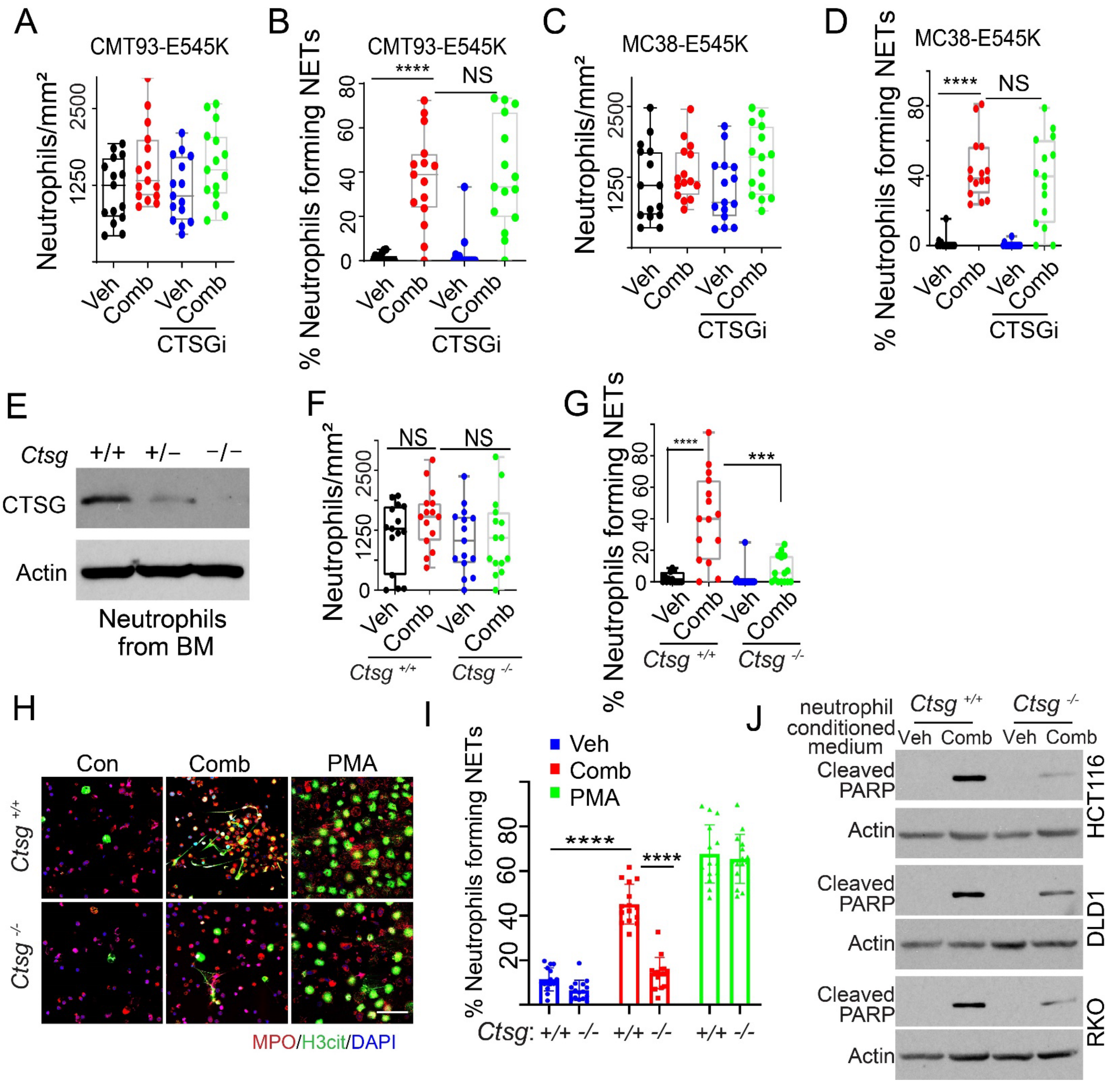

Figure S9. (A-D) Quantifications using the formula: area of MPO/total area*100 (A), area of H3-cit/total area*100 (B), numbers of MPO positive cells per mm^2 (C), or (number of NET-forming neutrophils/number of neutrophils)*100 (D) (n=15/group). (**E**) Cell lysates of purified neutrophils from the indicated genotypes were blotted with an anti-CTSG antibody. (**F & G**) Quantifications using the formula: numbers of MPO positive cells per mm^2 (F), or (number of NET-forming neutrophils/number of neutrophils)*100 (G) (**H & I**) Neutrophils from CTSG WT and KO mice were treated with the indicated drugs overnight and were stained with anti-MPO and anti-H3cit antibodies. Representative images are shown in (H). Quantifications shown in I (n=15/group). (**J**) The indicated CRC cells were treated with conditioned media from (H), and cell lysates were blotted with the indicated antibodies. Scale bar: 50 µm. One-way ANOVA (A to D) or Two-way ANOVA (F, G & I) was used for statistical analysis. *p<0.05; **p<0.01; ***p<0.001; ****p<0.0001.

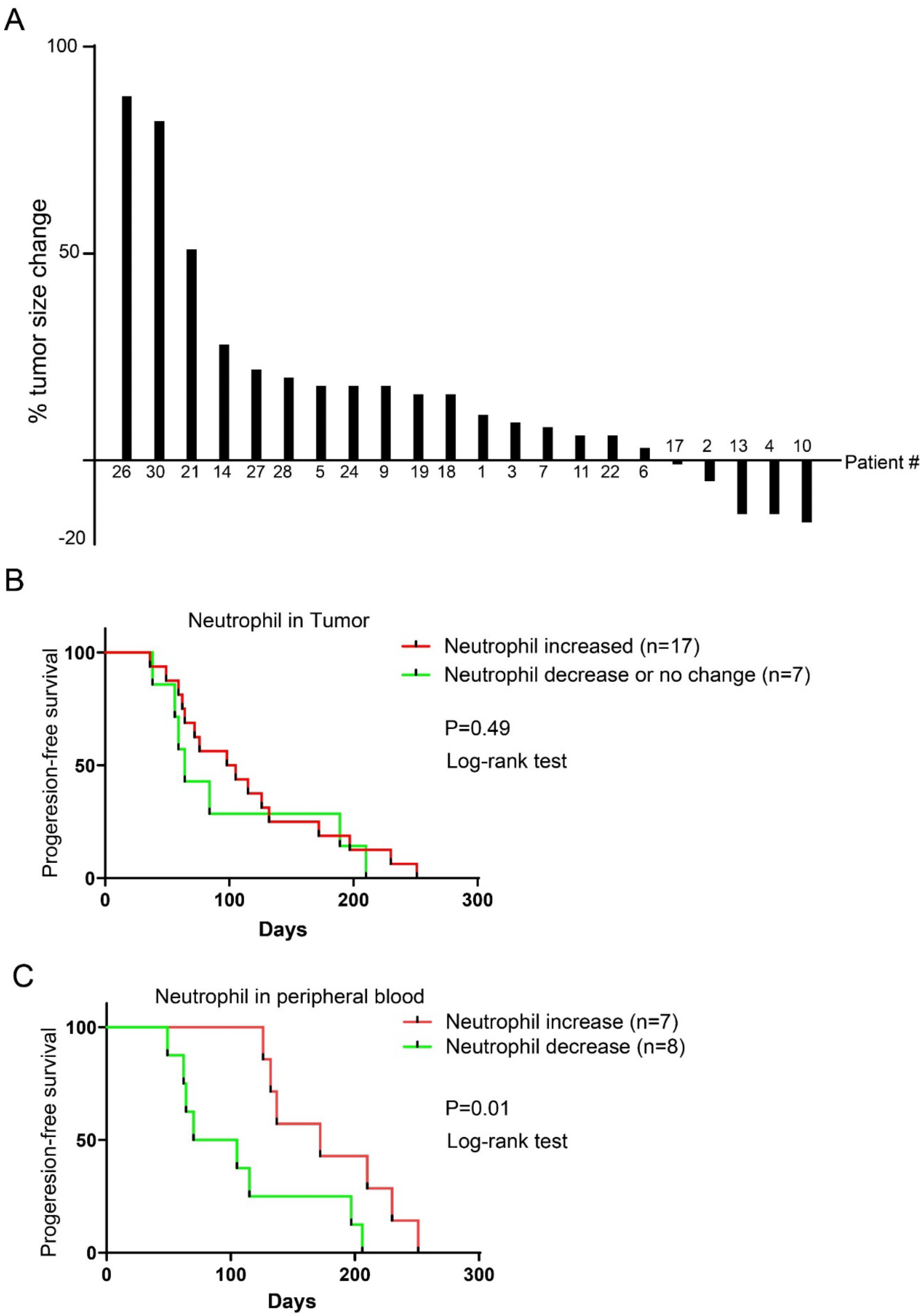

Figure S10. Clinical data of the phase II clinical trial patients. (A) Waterfall plot of tumor size changes. **(B)** Kaplan-Meier curves of PFS patients with increased numbers of tumor-infiltrating neutrophils in posttreatment biopsies compared to pretreatment biopsies vs those with decreased or no change numbers of tumor-infiltrating neutrophils in posttreatment biopsies. **(C)** Kaplan-Meier plot of progression-free survival of patients with increased longitudinal peripheral neutrophil counters during the drug treatment vs those with decreased longitudinal peripheral neutrophil counters.

Supplementary Methods

Somatic Gene Targeting

Targeting vectors were constructed with the USER system, and targeted cells were generated as described previously (2). The strategy of knock in PIK3CA E545K and knockout of IL-8, NRF2 CXCL1, and CCDC25 are shown in diagrams (Figs. S3 K, Q, & I, Fig. S6A). Briefly, homologous arms were PCR-amplified from human genomic DNA using HiFi Taq (Invitrogen) and inserted into the ZV37 vector. The E545K mutation was introduced by site-directed mutagenesis and validated by sequencing. The gRNA constructs were made by inserting the DNA sequences encoding gRNA into the px330 vector (Addgene). The px330-gRNA construct (1µg) and targeting vector (2 µg) were co-transfected into the target cells in a well of a 6-well plate using Lipofectamine 3000. After 24 hours, cells were replated in ten 96-well plates in media containing geneticin (Invitrogen) at a final concentration of 0.5-1 mg/ml. Drug-resistant colonies were grown for 10-14 days for PCR screening. Targeted clones were sequenced and validated by western blots. For PIK3CA E545K knock-in cells, to remove the drug resistance marker from correctly targeted clones, cells were infected with an adenovirus that expresses the Cre recombinase, as described (2). Cells were plated using limiting dilution in media without geneticin. Single clones were plated in duplicate in media with or without 0.5 mg/ml geneticin. After one week of growth, clones that were geneticin-sensitive were expanded for further analysis. Primers for targeting vector construction and PCR screening are listed in Table S5.

Intra-tumor injection

For DNase I treatment, once the tumors reached 100-200 mm^3, tumors were injected daily with either 30µl of vehicle control or 3 mg/ml DNase1, which was dissolved in PBS with 1 mM Ca^{2+}/Mg^{2+}, for 3 weeks. For CTSG inhibitor treatment, tumors were injected daily with either vehicle control or 30 µl 1 mM CTSG inhibitor (prepared in saline with 1% DMSO) for 3 weeks.

NK cell depletion

Mice were treated with control IgG or an anti-GM1 antibody (63 µg antibody in 100 µl) before inoculating tumor cells. And continue treatment for 4 weeks twice a week. Once the tumors reached 100-200 mm^3, mice were randomly grouped into two groups with vehicle control and drug combination. Tumor volume was measured with calipers, and volumes were calculated as length × width^2/2.

Macrophage depletion

Liposomal clodronate and control liposomes were purchased from Encapsula Nanosciences LLC (Brentwood, TN, USA) and administered to mice according to the manufacturer's instructions. Briefly, 100 µl of 18.4 mM Clodronate disodium salt in 35 mM liposome or an equivalent volume of control liposome was injected i.p. into mice twice a week. Mice were inoculated with two million tumor cells one day after the first liposome injection.

Neutrophil depletion

Once the xenograft tumor reached an average size of 150mm^3, mice were treated i.p. every other day with 100 µg of a rat anti-mouse Ly6G (clone 1A8, Bio X cells) or rat IgG2b (isotype control, Bio X cells) for 3 weeks. The neutrophil depletion antibody was injected 6 hours before the drug treatment.

Neutrophil isolation

Neutrophils were isolated from the bone marrow of 8 to 12-week-old C57/BL6 mice using the Neutrophil Isolation Kit (Biolegend) according to the manufacturer's instructions. The purity of neutrophils was assessed by flow cytometry with antibodies against CD11b$^+$ and Ly6G$^+$. NETs media were prepared by treating purified neutrophils with 1 µm CB839 and 1µm 5-FU for 4 hours, followed by centrifugation at 500 g for 5 min. The cleared media were transferred to new tubes. The control media were collected by treating HCT116, DLD1, or RKO cells similarly.

Immunofluorescent staining

Slides were immersed with xylene for 10 min twice, followed by rehydrating with gradient ethanol: 100%, 95%, 75%, and 50% each for 2 min. After being washed with water twice, the slides were performed with an antigen-retrieve step as follows: boiled the slides in 250 ml 1X Dako antigen-retrieve buffer for 2 min at 100% power 1200 w microwave, followed by 18 min continuous 30% power heating, cooling down the liquid for at least 30 min. The slides were then blocked with 0.1% Triton-X100 in 5% BSA for 30 min at room temperature, followed by incubating with a primary antibody overnight. After the slides were washed 3 times by PBS, a secondary antibody prepared in blocking buffer was added and incubated for 1 hour at room temperature. After washing 4 times with PBS, slides were mounted with a prolonged anti-fade solution with DAPI and stored at -20 ºC before visualization.

Quantification of immunostaining

The NETs were determined as the percentage of the positive H3cit signal in each field of view in the overall tissues using the Celleste Image Analysis Software. For neutrophil quantification, the percentage of neutrophils was calculated using the formula: (area of MPO/total area)*100. For NET quantification, the percentage of NETs was calculated using the formula: (area of H3cit/total area)*100, as described (3).

Alternatively, neutrophils were counted as MPO-positive cells, and NET-forming neutrophils were counted as MPO and extracellular citrullinated H3-positive cells from at least 15 randomly selected representative immunofluorescence images from three different mice. The percentage of NET-forming neutrophils was calculated using the formula: (number of NET-forming neutrophils/number of neutrophils)*100, as described (4).

In vitro netosis induction

Purified neutrophils ($2.5x10^5$) were seeded on 0.1% poly-L-lysine coated coverslips and incubated at 37 ºC for 30 min. Cells were treated with drugs or a conditioned medium for 16 hours and then fixed by 4% PFA for 10 min at room temperature. After permeabilization with 0.1% Triton-X100 in 5% BSA for 30 min, cells were stained with fluorescent-conjugated antibodies against citrullinated histone H3 or MPO, as described in the above section. The photographs were taken by a confocal microscope (Leica SP8), and quantification was performed using the Celleste software (Invitrogen), as described in the following section.

RNA-seq

One million DLD1 WT-only and Mutant-only cells were treated with vehicle or 5 μM CB-839 plus 5 μM 5-FU overnight. RNAs were extracted using a RNeasy kit (QIAGEN). Illumina high-throughput sequencing libraries were constructed and sequenced according to the manufacturer's instructions.

First, quality control of sequencing reads was performed using FastQC (v0.11.9). Reads were then aligned to the GRCh38/hg38 Homo sapiens reference genome using HISAT2 (v2.2.1). Transcript assembly and abundance quantification were conducted via StringTie (v2.1.5). After FPKM Normalization with Ballgown (v2.32.0), different gene expression analysis was conducted with edgeR's exact test (edgeR v3.42.4). Pairwise comparisons were performed with edgeR's decideTestsDGE function and Benjamini-Hochberg (BH)-adjusted p-value cutoff of 0.05 was used to obtain DEGs (absolute log2[Fold-Change] greater than or equal to 1). Gene set enrichment analysis (GSEA) was performed using the human hallmark gene set from MSigDB (v7.5.1). Significantly enriched pathways had BH-adjusted p-value < 0.05 and minimum gene set size of 10. Data visualizations were conducted through R (v4.3.1) packages.

Cell death assays

Cancer cells were plated in serum-free DMEM/F12 medium with 70-80% confluence in 96-well, 24-well, or 6-well plates. Cells were treated with either conditional medium or recombinant proteins for 16-24 hours. For cell viability assay, five μL CCK8 solution (Dojindo, Rockville, MD) was added into each well of a 96-well plate and incubated for 2-4 hours. The absorbance of 450 nm was measured using a microplate reader (Promega). The caspase 3 activity assay was measured 24 hours after treatment using Caspase-Glo3/7 Assay Systems according

to the manufacturer's instructions (Promega). For Annexin V staining, cells from 6-well plates were stained with FITC Annexin V Apoptosis Detection Kit (BD PharMingen) according to the manufacturer's instructions. Samples were analyzed using a BD LSR II flowcytometer (BD PharMingen). For cleaved PARP detection, cells from 24 wells were lysed with RIPA buffer for Western blot, as described below.

Western blotting

Cultured cells or tumors were lysed with lysis buffer [50 mM Tris pH 7.4, 150 mM NaCl, 5 mM EDTA, 0.5% NP40, 0.1% SDS, 1 mM PMSF, complete Protease Inhibitor Cocktail (Roche), supplemented with phosphatase inhibitors]. Then, lysates were cleared by centrifugation (14,000 rpm, 10 min), and protein concentration was determined by the BCA protein assay kit (Pierce). Tumor tissues were homogenized in lysis buffer on ice for 10 min. Equal amounts of total protein were used for immunoblotting as described (5).

Chromatin Immunoprecipitation (ChIP)

ChIP was performed as previously described (6). Briefly, 10 million HCT116 or CMT93 *PIK3CA* E545K cells were treated with 10 μM CB839 and 10 μM 5-FU overnight and then cross-linked with 1% formaldehyde for 10 minutes at room temperature, followed by glycine termination. Cells were lysed in 0.5 mL nuclear lysis buffer (50 mM Tris-HCl, pH8.0, 10 mM EDTA, 0.5%SDS) and subjected to sonication. Two hundred μl of the sonicated nuclear lysate was diluted in 800 μl dilution buffer (20 mM Tris-HCl, 150 mM NaCl, 2 mM EDTA, 1% TritonX-100) and incubated with normal IgG or an anti-NRF2 antibody for HCT116 cells, or an anti-p65 antibody for CMT93 *PIK3CA* E545K cells overnight followed by incubating with Protein A-conjugated magnetic beads for 2 hours at 4°C. The samples were washed with RIPA buffer (20 mM Tris-HCl, pH8.0, 150 mM NaCl, 2mM EDTA, 1% Triton-X100, 0.1% SDS) 8 times, washing buffer (20 mM Tris-HCl, pH8.0, 500 mM NaCl, 2 mM EDTA, 1%Triton-X100, 0.1% SDS) twice, and TE buffer (10mM Tris-HCl, pH8.0, 1 mM EDTA) twice. The immunoprecipitation products were eluted with 200 μL elution buffer (1% SDS, 0.1 M NaHCO3) followed by reverse cross-linked chromatin at 65°C overnight. After being treated with proteinase K (0.3 μg/μl) at 37°C for 2 hours, DNAs were purified using the PCR purification kit (Qiagen). Purified DNA was used for quantitative PCR analyses and was normalized to 1% input chromatin.

Reactive oxygen species (ROS) measurement

Reactive oxygen species (ROS) was measured by labeling cells with H2-carboxy-DCFDA (Invitrogen) according to the manufacturer's instructions. Briefly, neutrophils were cultured and treated with the indicated agents for 2 hr at 37 ºC. The tissue culture media were removed, and 100 μl of diluted DCFDA solution was added into each well and incubated for 30-45min at 37 ºC. The DCFDA solutions were removed and replaced with dilution buffer. Fluorescent signals were measured with a plate reader at an excitation wavelength of 485 nm and an emission wavelength of 535nm, respectively.

ELISA assay

Human IL8 was measured by the ELISA MAX^{TM} IL8 kit (Biolegend) according to the manufacturer's instructions. Briefly, anti-IL8 or CXCL5 antibody was coated in a 96-well plate overnight at 4 ºC. After being washed three times with 1xPBST, the plates were blocked with a blocking buffer for 30 min at room temperature. Tissue media or tissue lysates were added into the antibody-coated well and incubated for 2 hr at room temperature. The plates were washed three times with 1x PBST, and incubated with biotin-conjugated detection antibody for 1 hr at room temperature. The plates were three times with PBST, incubated with HRP-streptavidin for 1 hr, and then TMB solution for 15 – 30 min. OD450nm was measured with Promega Glomax. Mouse CXCL5 was measured using the mouse LIX ELISA Kit (Abcam) according to the manufacturer's instructions.

Cytokine array

The culture media from DLD1 *PIK3CA* WT or isogenic *PIK3CA* E545K mutant cells were collected, and the protein concentration was measured using a BCA kit. Membranes of cytokine array (company) were blocked with

blocking buffer according to the manufacturer's instructions, then incubated overnight at 4 ºC with an equal amount of DLD1 *PIK3CA* WT or E545K mutant culture medium with protease inhibitor cocktail being added. The membranes were washed, incubated with a biotin-conjugated antibody and then HRP-conjugated streptavidin, and proceeded with chemiluminescence.

Real-time PCR

Total RNAs were extracted using RNeasy Mini Kit (Qiagen) following the manufacturer's instructions. cDNAs were synthesized using the Transcriptor First Strand cDNA Synthesis Kit (Roche). Quantitative PCRs were performed using iTaq™ Universal SYBR® Green Supermix (Bio-rad) on a CFX Connect™ Real-Time PCR Detection System (Bio-Rad). Gene expression levels were determined using the $2^{-\Delta\Delta Ct}$ method, normalizing to housekeeping genes GAPDH or ACTIN. The primers used are listed in Table S5.

RNA interference

Interfering RNAs (siRNA) targeting NRF2, p65, or non-silencing controls were synthesized by IDT. For cell transfection, RNAi liposomes were generated using Lipofectamine 3000 with 10 nM siRNA in Opti-MEM. Cells were harvested 48 to 72 hr post-transfection for the indicated analyses.

In vitro protease cleavage assay

Recombinant 14-3-3 epsilon (1 µg) was incubated with 100 ng CTSG at 37 for 0 min,10 min, 30min, and 90 min in 1x reaction buffer (50 mM Tris-HCl pH 7.6, 100 mM NaCl), respectively. The reactions were stopped by adding 2x SDS sample buffer and boiling for 5 min. For NETs cleavage assay, 5 µL conditioned media (about 300 ng protein) were mixed with 1 µg recombinant 14-3-3 protein in 1x reaction buffer with or without 1 µM CTSGi or DNAse 1 (1-30 mg/ml) and incubated for 4 hours at 37 ºC.

Table S1. Differential expressed genes by RNA-seq analysis

gene_id	gene_name	log2FC	logCPM	PValue	adjusted.PValue
MSTRG.847	ARTN	3.343532661	3.788761735	2.75E-04	1.11E-02
MSTRG.20682	CXCL8	2.970947615	7.676924674	8.97E-74	9.05E-71
MSTRG.20685	CXCL2	2.919269512	6.275318697	9.76E-24	4.28E-21
MSTRG.26186	CCNE2	2.489077419	5.106202908	2.24E-05	1.22E-03
MSTRG.2505	ATF3	2.4127281	6.827599176	1.07E-32	6.37E-30
MSTRG.14102	GDF15	2.307890706	10.7057096	0.00E+00	0.00E+00
MSTRG.6189	NR4A1	2.294738379	6.333483544	7.28E-22	2.82E-19
MSTRG.23236	CDKN1A	2.277744928	9.582460862	8.88E-189	4.48E-185
MSTRG.21554	HMGCS1	2.197597058	7.312219017	6.92E-20	2.25E-17
MSTRG.26496	ARC	2.166195594	5.114048058	7.51E-07	5.42E-05
MSTRG.2833	IDI1	1.978169573	6.041544519	6.68E-10	6.88E-08
MSTRG.1266	CYR61	1.941469129	5.926353172	1.17E-06	8.19E-05
MSTRG.22314	HBEGF	1.889547297	5.084774054	7.67E-06	4.55E-04
MSTRG.1842	SPRR2D	1.859919219	5.103290844	3.59E-07	2.85E-05
MSTRG.565	SESN2	1.85230723	5.913296412	3.37E-10	3.58E-08
MSTRG.12509	PPM1D	1.848108082	6.189905602	5.84E-12	6.93E-10
MSTRG.514	SFN	1.844497614	10.29690103	5.31E-182	1.79E-178
MSTRG.12055	KRTAP2-3	1.832607698	6.150142326	7.72E-13	1.04E-10
MSTRG.21210	MSMO1	1.770307837	7.491358619	1.17E-21	4.38E-19
MSTRG.17558	CH507-42P11.8	1.76835011	4.895177737	8.17E-05	3.89E-03
MSTRG.28444	TSC22D3	1.746573527	5.279272005	2.40E-06	1.59E-04
MSTRG.20684	CXCL3	1.740405617	5.742619875	2.47E-04	1.02E-02
MSTRG.3323	UNC5B-AS1	1.718252991	4.652620394	1.66E-03	4.83E-02
MSTRG.2387	BTG2	1.712858704	7.023878345	3.13E-19	9.02E-17
MSTRG.13408	SERPINB5	1.684300468	6.085734352	2.21E-09	2.10E-07
MSTRG.17825	SIK1	1.668188721	4.861263702	1.75E-04	7.49E-03
MSTRG.8314	FOS	1.66640561	5.219460053	5.27E-06	3.24E-04
MSTRG.11035	RP11-505K9.4	1.652868427	5.665393581	5.99E-07	4.38E-05
MSTRG.11035	MLYCD	1.652868427	5.665393581	5.99E-07	4.38E-05
MSTRG.11035	OSGIN1	1.652868427	5.665393581	5.99E-07	4.38E-05
MSTRG.14666	PPP1R15A	1.635850204	7.023301032	7.35E-16	1.61E-13
MSTRG.22560	DUSP1	1.634734803	6.762033835	1.80E-17	4.53E-15
MSTRG.2334	PHLDA3	1.627822157	6.423479477	4.97E-12	5.96E-10
MSTRG.2334	RP11-134G8.10	1.627822157	6.423479477	4.97E-12	5.96E-10
MSTRG.25501	INSIG1	1.61427098	7.335923699	1.09E-19	3.22E-17
MSTRG.9592	AEN	1.605996591	6.785951074	1.61E-13	2.29E-11
MSTRG.16128	NR4A2	1.53890187	5.793907367	1.91E-07	1.57E-05
MSTRG.876	PLK3	1.517489419	6.729368293	8.66E-13	1.15E-10
MSTRG.1060	JUN	1.505798267	6.93600554	8.28E-15	1.39E-12
MSTRG.8177	ARG2	1.484502506	5.067272479	1.64E-03	4.81E-02
MSTRG.1159	GADD45A	1.464063086	8.106811343	5.90E-32	3.31E-29
MSTRG.6807	MVK	1.450265705	5.673863006	3.37E-04	1.31E-02
MSTRG.4870	FGF19	1.431569667	5.688779209	1.61E-05	9.00E-04

MSTRG.13386	PMAIP1	1.427578781	7.335446826	2.75E-16	6.31E-14
MSTRG.19018	CDC25A	1.41808382	5.681116372	8.02E-04	2.62E-02
MSTRG.24962	SERPINE1	1.413574547	6.044243538	4.08E-05	2.07E-03
MSTRG.26105	CA2	1.405720218	6.523412306	9.31E-10	9.49E-08
MSTRG.18889	CSRNP1	1.390827596	5.8491317	4.32E-06	2.77E-04
MSTRG.25615	FDFT1	1.367860591	7.949119129	5.07E-17	1.22E-14
MSTRG.24102	ACAT2	1.36298333	6.226221219	1.21E-04	5.41E-03
MSTRG.26162	GEM	1.359955711	6.610013361	2.66E-11	2.98E-09
MSTRG.17190	ACSS2	1.327056529	5.657337501	7.30E-04	2.49E-02
MSTRG.3624	AVPI1	1.322931716	6.912940666	9.94E-13	1.30E-10
MSTRG.23987	ULBP2	1.322248632	5.582882903	3.71E-04	1.41E-02
MSTRG.11117	MVD	1.318710899	6.172644109	1.14E-06	8.05E-05
MSTRG.21637	PLK2	1.313050413	7.605825864	1.07E-14	1.78E-12
MSTRG.2065	HSD17B7	1.299096561	5.920521336	7.56E-04	2.52E-02
MSTRG.27413	LHX2	1.295162081	5.18961122	1.09E-03	3.38E-02
MSTRG.16885	TRIB3	1.279285999	7.291475247	1.11E-09	1.12E-07
MSTRG.6365	DDIT3	1.268485507	5.715648189	1.94E-05	1.07E-03
MSTRG.4897	DHCR7	1.266485307	8.065991105	6.80E-17	1.60E-14
MSTRG.11720	TRAF4	1.262473253	7.477102851	2.24E-12	2.75E-10
MSTRG.11964	DUSP14	1.259381691	6.677582588	3.73E-07	2.94E-05
MSTRG.24831	LRRD1	1.246386823	7.597493598	1.85E-13	2.56E-11
MSTRG.24831	CYP51A1	1.246386823	7.597493598	1.85E-13	2.56E-11
MSTRG.6964	TRIAP1	1.245082008	7.07221742	5.58E-11	6.18E-09
MSTRG.16389	INPP1	1.23625863	5.858294465	4.52E-04	1.63E-02
MSTRG.441	E2F2	1.1982052	5.444849953	1.86E-03	5.34E-02
MSTRG.14614	BBC3	1.192930892	7.2534033	7.39E-13	1.01E-10
MSTRG.13854	LDLR	1.190877794	7.111717215	1.20E-09	1.19E-07
MSTRG.13854	MIR6886	1.190877794	7.111717215	1.20E-09	1.19E-07
MSTRG.43	ISG15	1.164776288	9.19207575	9.44E-39	6.35E-36
MSTRG.26393	SQLE	1.148014078	7.039681079	2.05E-06	1.37E-04
MSTRG.4501	DDB2	1.130482382	5.875513712	2.13E-04	8.93E-03
MSTRG.6707	DRAM1	1.130013871	5.722228686	8.78E-04	2.79E-02
MSTRG.654	RNF19B	1.126237415	5.679797527	7.32E-04	2.49E-02
MSTRG.27196	ZNF367	1.122215247	5.922872482	4.25E-04	1.55E-02
MSTRG.17988	RTN4R	1.113340672	5.941008451	1.47E-03	4.38E-02
MSTRG.3394	PLAU	1.111361606	6.753133028	3.15E-08	2.76E-06
MSTRG.3625	RP11-548K23.11	1.105604553	6.191745659	1.01E-04	4.55E-03
MSTRG.3625	PI4K2A	1.105604553	6.191745659	1.01E-04	4.55E-03
MSTRG.3625	HOGA1	1.105604553	6.191745659	1.01E-04	4.55E-03
MSTRG.13727	TNFSF9	1.102733803	7.183001461	1.28E-09	1.25E-07
MSTRG.15846	FHL2	1.099012566	7.232083587	6.40E-10	6.66E-08
MSTRG.13174	NPC1	1.096055716	5.944968813	2.13E-04	8.93E-03
MSTRG.2239	IER5	1.084565637	6.874519649	1.08E-06	7.73E-05
MSTRG.1049	PCSK9	1.079505053	6.468722397	9.90E-06	5.74E-04
MSTRG.21782	HMGCR	1.067691266	7.330486415	5.35E-09	4.91E-07
MSTRG.14196	CCNE1	1.067530993	6.108452349	8.10E-04	2.63E-02
MSTRG.19143	DUSP7	1.044156571	5.714937121	1.40E-03	4.21E-02

MSTRG.18286	HMOX1	1.03545246	6.10336606	2.16E-04	8.98E-03
MSTRG.12704	FDXR	1.029025366	7.431809357	1.53E-09	1.48E-07
MSTRG.2008	AL121987.1	1.023372305	6.55392568	1.80E-04	7.63E-03
MSTRG.2008	PEA15	1.023372305	6.55392568	1.80E-04	7.63E-03
MSTRG.21295	PDLIM3	1.006788932	5.56858592	5.34E-04	1.88E-02
MSTRG.17384	CEBPB	1.001603908	8.297538385	1.92E-17	4.72E-15
MSTRG.9223	PIF1	-2.75286413	4.25161114	1.31E-03	3.96E-02
MSTRG.27829	MT-TR	-2.37262802	4.943781707	8.96E-06	5.26E-04
MSTRG.27831	MT-TH	-2.30710648	5.2578924	3.31E-06	2.15E-04
MSTRG.27832	MT-TS2	-2.21006785	5.412581856	4.52E-07	3.45E-05
MSTRG.27827	MT-TG	-2.139369	5.383781026	4.59E-07	3.48E-05
MSTRG.27833	MT-TL2	-2.1354388	5.312000351	2.06E-06	1.37E-04
MSTRG.1436	PSRC1	-1.90617303	4.896899373	8.78E-04	2.79E-02
MSTRG.22269	KIF20A	-1.67927166	6.642967407	1.80E-11	2.04E-09
MSTRG.15683	MAT2A	-1.67536043	6.607529617	7.25E-06	4.33E-04
MSTRG.22532	HMMR	-1.62971719	6.277583648	4.22E-07	3.25E-05
MSTRG.21694	CCNB1	-1.38663647	8.113860939	3.95E-20	1.33E-17
MSTRG.10304	PLK1	-1.25498506	5.751855492	7.67E-04	2.52E-02
MSTRG.10304	DCTN5	-1.25498506	5.751855492	7.67E-04	2.52E-02
MSTRG.10304	CTD-2196E14.9	-1.25498506	5.751855492	7.67E-04	2.52E-02
MSTRG.8214	SRSF5	-1.23926973	6.488153888	4.50E-04	1.63E-02
MSTRG.19951	RPL22L1	-1.19223396	7.784619154	1.45E-12	1.85E-10
MSTRG.17422	AURKA	-1.182594	6.795462523	5.34E-07	3.99E-05
MSTRG.15897	BUB1	-1.15281757	7.097276687	2.90E-08	2.57E-06
MSTRG.838	CDC20	-1.14979934	8.454415797	7.80E-20	2.38E-17
MSTRG.838	MPL	-1.14979934	8.454415797	7.80E-20	2.38E-17
MSTRG.9142	CCNB2	-1.10972782	6.73542428	7.96E-06	4.69E-04
MSTRG.2481	NEK2	-1.0905505	6.590802795	5.27E-06	3.24E-04
MSTRG.25195	RP11-155G14.6	-1.08330375	5.57885656	3.41E-04	1.31E-02
MSTRG.25195	HILPDA	-1.08330375	5.57885656	3.41E-04	1.31E-02
MSTRG.25195	RP11-212P7.3	-1.08330375	5.57885656	3.41E-04	1.31E-02
MSTRG.25195	METTL2B	-1.08330375	5.57885656	3.41E-04	1.31E-02
MSTRG.25195	RP11-155G14.5	-1.08330375	5.57885656	3.41E-04	1.31E-02
MSTRG.2521	CENPF	-1.04635224	6.050163311	6.75E-04	2.33E-02
MSTRG.8030	DLGAP5	-1.04029827	6.330141006	5.63E-05	2.81E-03

Table S2. Patient Baseline Characteristics.

	Number of Patients (%), n =32
**Gender**	
Male	14 (44)
Female	18 (56)
**Race**	
White	28 (87.5)
African American	3 (9.4)
Asian	1 (3.1)

Table S3. Adverse Events (all grade 3/4 AE and grad 1/2 AE seen in > 10% of patients)

Adverse Event	Toxicity Grade: Number (%), n=32 Any Grade	Grade 3	Grade 4	Grade 5
**Hematalogic/Laboratory**				
Alanine aminotransferase increased	9 (28)	1 (3)		
Alkaline phosphatase increased	8 (25)	1 (3)		
Anemia	12 (38)	2 (6)		
Aspartate aminotransferase increased	14 (44)	3 (9)		
Blood bilirubin increased	11 (34)	3 (9)	2 (6)	
Creatinine increased	5 (16)			
Hyperglycemia	8 (25)	1 (3)		
Hypoalbuminemia	3 (9)			
Hypokalemia	8 (25)		1 (3)	
Hypomagnesemia	7 (22)	1 (3)		
Hyponatremia	9 (28)	4 (13)		
Hypophosphatemia	9 (28)	3 (9)		
Lactic acidosis	1 (3)	1 (3)		
Lymphocyte count decreased	9 (28)	2 (6)	2 (6)	
Neutrophil count decreased	1 (3)	1 (3)		
Platelet count decreased	4 (13)			
WBC count decreased	5 (16)	1 (3)		
**Non-Hematologic/Laboratory**				
Abdominal pain	7 (22)			
Anorexia	8 (25)			
Back pain	2 (6)	1 (3)		
Biliary tract infection	1 (3)			1 (3)
Constipation	6 (19)			

Dehydration	4 (13)	2 (6)	
Diarrhea	12 (38)	1 (3)	
Dyspepsia	4 (13)		
Dyspnea	3 (9)	1 (3)	
Eye disorder	6 (19)		
Fatigue	15 (47)	1 (3)	
Generalized muscle weakness	4 (13)	1 (3)	
Hepatic Failure	1 (3)		1 (3)
Hypertension	4 (13)		
Mucositis oral	6 (19)		
Nausea	14 (44)	4 (13)	
Pain	6 (19)		
Pain in extremity	4 (13)	1 (3)	
Palmar-plantar erythrodysesthesia syndrome	11 (34)	1 (3)	
Sigmoid diverticulosis	1 (3)	1 (3)	
Small intestinal obstruction	1 (3)		1 (3)
Skin and subcutaneous tissue disorders	7 (22)		
Skin infection	1 (3)	1 (3)	
Vomiting	7 (22)	3 (9)	

Table S4 Reagents

REAGENT or RESOURCE	SOURCE	IDENTIFIER
Antibodies		
Goat anti-MPO	R&D systems	Cat# AF3667 RRID: AB_2250866
Rabbit anti-H3CIT	Abcam	Cat# ab5103 RRID : AB_304752
anti-Ly6G (flow cytometry)	Biolegend	Cat# 127601 RRID : AB_1089179
anti- CD11b (flow cytometry)	Biolegend	Cat#163702 RRID: AB_2892339
Anti-NKP46	Biolegend	Cat#331902 RRID: AB_1027637
Anti-CD49b	Biolegend	Cat#108901 RRID: AB_313409
Anti-F4/80	Biolegend	Cat#157305 RRID: AB_2832548

Anti-NKP46 (IF staining)	Novus	Cat#AF2225 RRID: RRID:AB_355192
Anti-F4/80 (IF staining)	Cell Signaling Technology	Cat#70076 RRID : AB_2799771
anti-Ly6G (in vivo)	Bioxcell	Cat# BE0075-1 RRID : AB_1107721
Rat anti-IgG (in vivo)	Bioxcell	Cat# BE0089 RRID:AB 1107769
anti- 14-3-3 pan	Cell Signaling Technology	Cat# 95422 RRID: NA
anti- 14-3-3 α/β	Cell Signaling Technology	Cat# 9636 RRID:AB_560823
anti- 14-3-3 γ	Cell Signaling Technology	Cat# 5522 RRID: AB_10827887
anti- 14-3-3 δ	Cell Signaling Technology	Cat# 7413 RRID: AB_10950820
anti- 14-3-3 epsilon	Cell Signaling Technology	Cat# 9635 RRID: AB_2217758
anti- 14-3-3 τ	Cell Signaling Technology	Cat# 9638 RRID: AB_2218251
anti- 14-3-3 η	Cell Signaling Technology	Cat# 9640 RRID:AB_221807 3
Anti-RAGE	SANTACRUZ	Cat#sc-80652 RRID: AB_1128924
Anti human Cathepsin G	Millipore	Cat#MABT1240 RRID:AB_208752 7
Anti-cleaved PARP	Cell Signaling Technology	Cat#5625 RRID: AB_10699459
Anti-bax	Cell Signaling Technology	Cat#5023 RRID: AB_10557411
Anti-cytochrome C	Cell Signaling Technology	Cat#4280 RRID: AB_10695410
Anti-VDAC	Cell Signaling Technology	Cat#4661 RRID: AB_10557420

Anti-BCL2	Cell Signaling Technology	Cat#15071 RRID: AB_2744528
Anti-IL8	R&D systems	Cat#MAB208 RRID: AB_2249110
anti-p65	Cell Signaling Technology	Cat#8242 RRID: AB_10859369
Anti-NRF2	Abcam	Cat#AB137550 RRID: AB_2687540
Anti-Actin	Sigma-Aldrich	Cat#A5441 RRID:AB_476744
Anti-CCDC25	Proteintech	Cat# 21209-1-AP RRID:AB_10732690
Anti-CXCL1	Proteintech	Cat# 12335-1-AP RRID:AB_2087568
Chemicals, Peptides, and Recombinant Proteins		
DMEM, high glucose medium	ThermoFisher Scientific	Cat#11995
McCoy's 5A medium	ThermoFisher Scientific	Cat#SH30200
DMEM/F12 1:1	ThermoFisher Scientific	Cat#11320
Penicillin-Streptomycin	ThermoFisher Scientific	Cat#15140122
PVDF membranes	Bio-rad	Cat#1620177
Trypsin digestion enzyme	ThermoFisher Scientific	Cat#25300054
Fetal Bovine Serum	VWR	Cat#1500
Recombinant human LL37 peptide	R＆D systems	Cat#5213
Recombinant human CTSG	Athens research technology	Cat #16-14-030107
Recombinant human NE	Athens research technology	Cat #16-14-051200
Recombinant human PR3	Athens research technology	Cat #16-14-161820
Recombinant human Lactoffrin	Athens research technology	Cat #16-14-120103
Recombinant human IL8	Peprotech	Cat#200-08
CTSG inhibitor	Millipore	Cat#219731
CB-839 for animal experiments	Calithra Biosciences	N/A
CB839 for in vitro experiments	Selleckchem	Cat#S7655
5-FU	Sigma	Cat#F6627
Sivelestat	Selleckchem	Cat#S8136
**Critical Commercial Assays**		
Mouse CXCL5 ELISA kit	Abcam	Cat#ab264611
Human IL8 elisa kit	biolegend	Cat#431570
Cellular ROS detect kit	Abcam	Cat#ab113851

Neutrophil isolation kit	Biolegend	Cat#480058
Caspase 3/7 detection kit	Promega	Cat#G8091
CCK8 KIT	Dojindo	Cat#CK04
Deposited Data		
Experimental Models: Cell Lines		
RKO	ATCC	CRL-2577
DLD1	ATCC	CCL-221
HCT116	ATCC	CCL-247
MC38	National Cancer Institute/NIH	N/A
CMT93	ATCC	CCL-223
Experimental Models: Organisms/Strains		
C57B6 mice	The Jackson Laboratory	
NSG mice	The Jackson Laboratory	
Nude mice	Case western reserve university	
CTSG knockout mice	Shanghai model organisms company	
Oligonucleotides		
NRF2-siRNA	Purchased from IDT	
P65-siRNA	Purchased from IDT	
Primers for PCR	This paper	Table S1
Recombinant DNA		
pX330	Addgene	Cat#42230
pTK-LoxP-NEO-AAV	Hao et al., 2013	N/A
Software and Algorithms		
GraphPad Prism	Graphpad	
Celleste image analysis software	Thermos fisher	

Table S5. Primers used in this study

sgRNA for RAGE KO F	CACC GTGGCTCGTGTCCTTCCCAA
sgRNA for RAGE KO R	AAAC TTGGGAAGGACACGAGCCAC
Left arm for RAGE KO F	GGGAAAG/ideoxyU/ ggcagttctctcctcacttg
Left arm for RAGE KO R	GGAGACA/ideoxyU/ GTTTTGAGCACCTACTACTGC
Right arm for RAGE KO F	GGTCCCA/ideoxyU/ ATCACAGCCCGGATTGGCGA
Right arm for RAGE KO R	GGCATAG/ideoxyU/ gttcacagggccgttttctac
sgRNA for IL8 KO F	CACC G CAAGAGAGCCACGGCCAGCT
sgRNA for IL8 KO R	AAAC AGCTGGCCGTGGCTCTCTTG C
Left arm for IL8 KO F	GGGAAAG/ideoxyU/ CAGAGACAGCAGAGCACACA
Left arm for IL8 KO R	GGAGACA/ideoxyU/ ACTCCTTGGCAAAACTGCAC

Right arm for IL8 KO F	GGTCCCA/ideoxyU/ GCTAAAGAACTTAGATGTCAG
Right arm for IL8 KO R	GGCATAG/ideoxyU/ CCCAGAATTTCCTGGGCAAAC
sgRNA for CXCL1 KO F	CACC GGAGCAGCAGTGCCACTCGC
sgRNA for CXCL1 KO R	AAAC GCGAGTGGCACTGCTGCTCC
Left arm for CXCL1 KO F	GGGAAAG/ideoxyU/ agggaagaaggaagataagc
Left arm for CXCL1 KO R	GGAGACA/ideoxyU/ TACCAGGAGCAGGAGCAGCA
Right arm for CXCL1 KO F	GGTCCCA/ideoxyU/ GCCGCTGGCCGGCGCGCAG
Right arm for CXCL1 KO R	GGCATAG/ideoxyU/ tcctctggcaccagagcagt
sgRNA for NRF2 KO F	CACC G CAAGCTGGTTGAGACTACCA
sgRNA for NRF2 KO R	AAAC TGGTAGTCTCAACCAGCTTGc
Left arm for NRF2 KO F	GGGAAAG/ideoxyU/ GACTGCATGCAGCTTTTGGC
Left arm for NRF2 KO R	GGAGACA/ideoxyU/ ACTTGCTCAATGTCCTGTTGC
Right arm for NRF2 KO F	GGTCCCA/ideoxyU/ TTGGGAGGAGCTATTATCCAT
Right arm for NRF2 KO R	GGCATAG/ideoxyU/ GATTCCACTGAGTGTTCTGG
sgRNA for E545K KI F	CACC G TTGTCTCTTCTGTCCGATGT
sgRNA for E545K KI R	AAAC ACATCGGACAGAAGAGACAA C
Left arm for E545K KI F	GGGAAAG/ideoxyU/ CACACACAAAAAAACACATAAG
Left arm for E545K KI R	GGAGACA/ideoxyU/ AGGCAGCAGCCCTGCAGTGC
Right arm for E545K KI F	GGTCCCA/ideoxyU/ GCATGTGGGGCAGGGATGC
Right arm for E545K KI R	GGCATAG/ideoxyU/ GCTTGCGTGTACAGTGTTTC
human IL8 CHIP 1-F1	TGCACTGTGTTCCGTATGCT
human IL8 CHIP 1-R1	ACCAATGGGACTGCTTTGCT
human IL8 CHIP 2-F1	GTGTTCACAGTGTGGGCAAAT
human IL8 CHIP 2-R1	AAAGAGCACCAAGGAAGGGT
human IL8 CHIP 3-F1	TCAGGACACTAGGACATAAAGCC
human IL8 CHIP 3-R1	TGGAAAGAGCATCTGGGTTCC
human IL8 CHIP 4-F1	TGCACCACTTTCTGGAGCAT
human IL8 CHIP 4-R1	CCAGTGGAGGCATAAGAGCA
human IL8 CHIP 5-F1	AGTATGCCCCCTAAGAGCAGT
human IL8 CHIP 5-R1	GGTGAAGATAAGCCAGCCAA
human IL8 CHIP 6-F1	TGAGGGTGCATAAGTTCTCTAGT
human IL8 CHIP 6-R1	ATGGTTCCTTCCGGTGGTTT
mouse CXCL1 RT PCT F2	TGGCTGGGATTCACCTCAAG
mouse CXCL1 RT PCT R2	CCGTTACTTGGGGACACCTT
mouse CXCL2 RT PCT F1	GCGCTGTCAATGCCTGAAGA
mouse CXCL2 RT PCT R1	TTTGACCGCCCTTGAGAGTG
MOUSE CXCL5 /6 GCP2 RT PCR F1	TAAAAGGGGTGCAGTGGGTT
MOUSE CXCL5 /6 GCP2 RT PCR R1	GAGCACCAGCTCGGGATATG
Human actin RT PCR F1	CTC TTC CAG CCT TCC TTC CT
Human actin RT PCR R1	AGC ACT GTG TTG GCG TAC AG
Mouse GAPDH RT PCR F1	TCG GTG TGA ACG GAT TTG
Mouse GAPDH RT PCR R1	GGT CTC GCT CCT GGA AGA
Human IL-8 RT-PCR F	CAGAGACAGCAGAGCACACA
Human IL-8 RT-PCR R	GGCAAAACTGCACCTTCACA

mouse CXCL5 CHIP 1-F1	GCACGCACGCACGCACGCA
mouse CXCL5 CHIP 1-R1	TTTCCTGCTCAGCAATGGAG
mouse CXCL5 CHIP 2-F1	TGTAGTAGGCCGGCAGAGGAA
mouse CXCL5 CHIP 2-R1	CCAAGGAAGCCAAAAGACGAC
mouse CXCL5 CHIP 3-F1	AAGAGAACTGGTTGCTGTGG
mouse CXCL5 CHIP 3-R1	AGGAAAAGAAGGGCATCCTC
mouse CXCL5 CHIP 4-F1	GAGGAGAATGCTTGTAGTCCT
mouse CXCL5 CHIP 4-R1	GCACGCACGCACGCACGCA
mouse gapdh genome PCR	GGG TTC CTA TAA ATA CGG ACT GC
mouse gapdh genome PCR	CTG GCA CTG CAC AAG AAG A
sgRNA for CCDC25 KO F	CACC GAAACTGACCTTGAATGCTAT
sgRNA for CCDC25 KO R	AAAC ATAGCATTCAAGGTCAGTTTC
Left arm for CCDC25 KO F	GGGAAAG/ideoxyU/ TACTGGTGGTAGTCCCAAG
Left arm for CCDC25 KO R	GGAGACA/ideoxyU/ ACAGTCCATCAGCACTTCCT
Right arm for CCDC25 KO F	GGTCCCA/ideoxyU/ GCCCACCTTGTGAAGGCCAA
Right arm for CCDC25 KO R	GGCATAG/ideoxyU/ ATTAGCAGAGCACCACCTCC

Supplement references:

1. Zhao Y, Feng X, Chen Y, Selfridge JE, Gorityala S, Du Z, et al. 5-Fluorouracil Enhances the Antitumor Activity of the Glutaminase Inhibitor CB-839 against PIK3CA-Mutant Colorectal Cancers. *Cancer Res.* 2020;80(21):4815-27.
2. Zhang X, Guo C, Chen Y, Shulha HP, Schnetz MP, LaFramboise T, et al. Epitope tagging of endogenous proteins for genome-wide ChIP-chip studies. *Nat Methods.* 2008;5(2):163-5.
3. Yang L, Liu Q, Zhang X, Liu X, Zhou B, Chen J, et al. DNA of neutrophil extracellular traps promotes cancer metastasis via CCDC25. *Nature.* 2020;583(7814):133-8.
4. Park J, Wysocki RW, Amoozgar Z, Maiorino L, Fein MR, Jorns J, et al. Cancer cells induce metastasis-supporting neutrophil extracellular DNA traps. *Sci Transl Med.* 2016;8(361):361ra138.
5. Chen Y, Zhao Y, Bajor DL, Wang Z, and Selfridge JE. A facile and sensitive method of quantifying glutaminase binding to its inhibitor CB-839 in tissues. *J Genet Genomics.* 2020;47(7):389-95.
6. Hao Y, He B, Wu L, Li Y, Wang C, Wang T, et al. Nuclear translocation of p85beta promotes tumorigenesis of PIK3CA helical domain mutant cancer. *Nat Commun.* 2022;13(1):1974.
