# Supplementary material for: Neutrophil extracellular traps induced by chemotherapy inhibit tumor growth in murine models of colorectal cancer
Source: J Clin Invest. 2024 Jan 9;134(5):e175031. doi: 10.1172/JCI175031 (PMC10904055; doi:10.1172/JCI175031)

Full unedited gel for figure 4

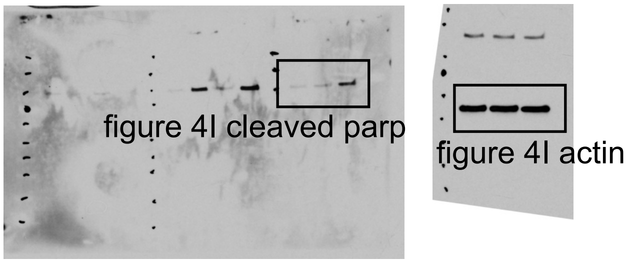

Full unedited gel for figure 5

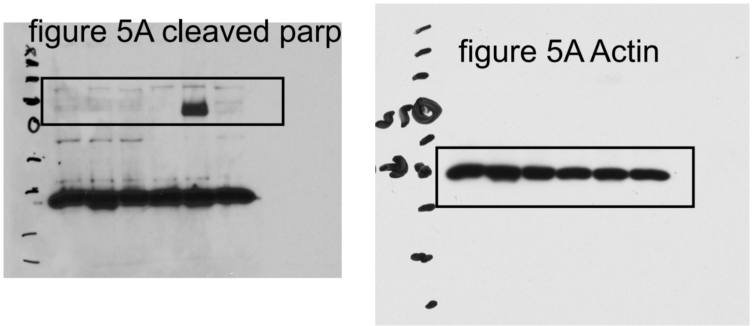

Full unedited gel for figure 6

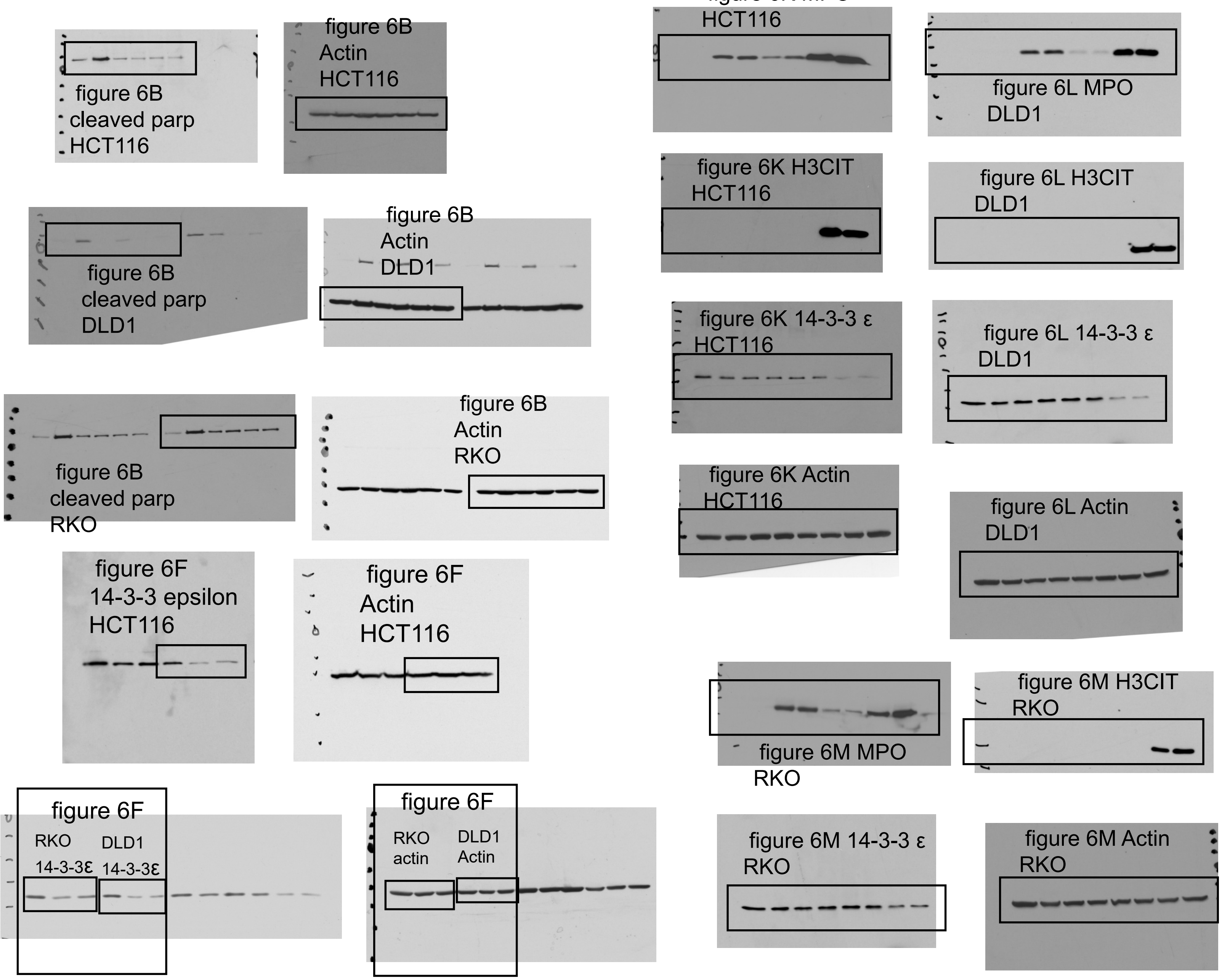

Full unedited gel for figure 6

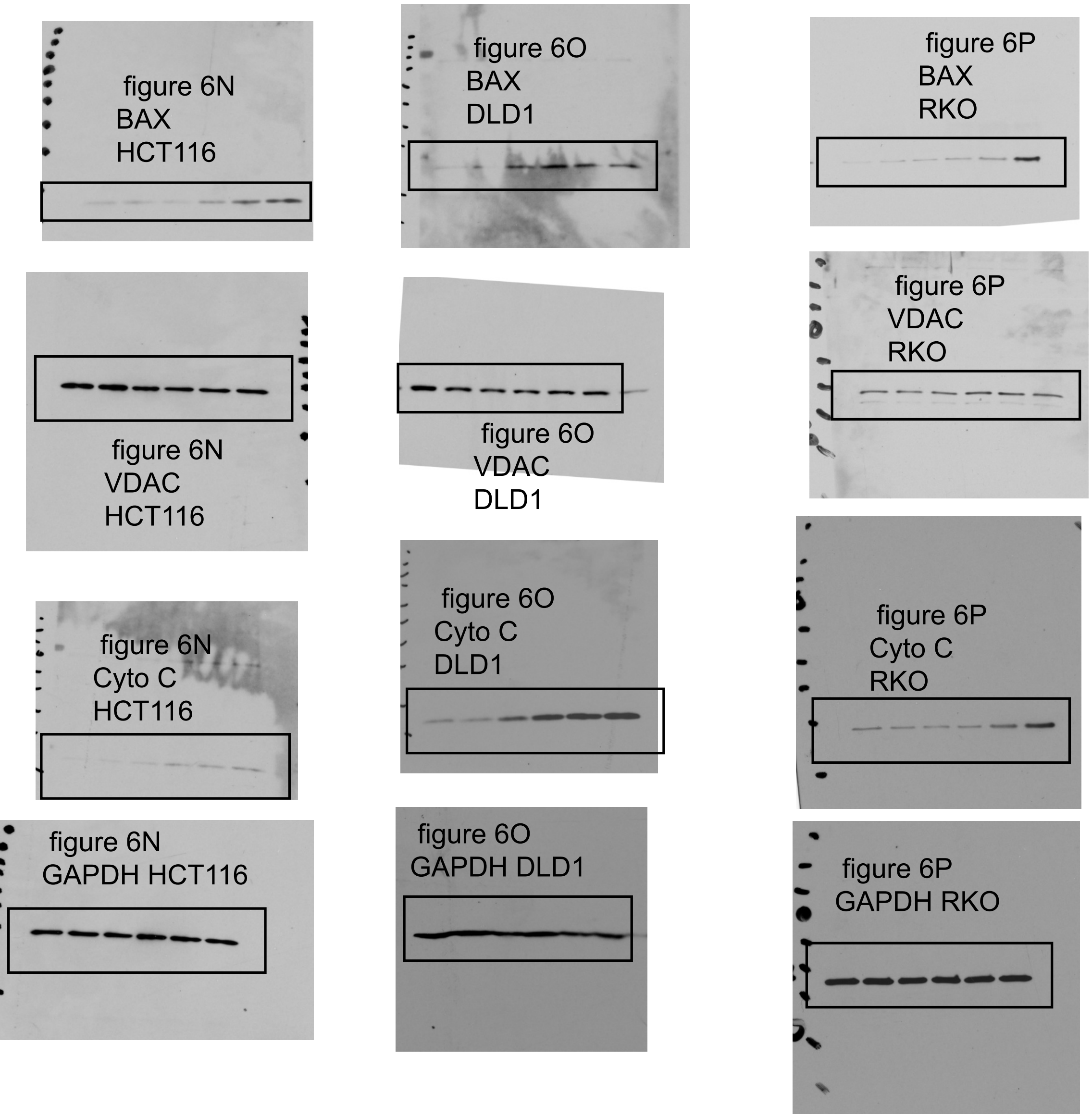

Full unedited gel for figure 9

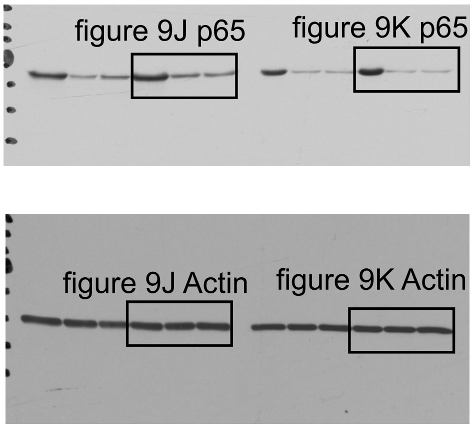

Full unedited gel for figure 10

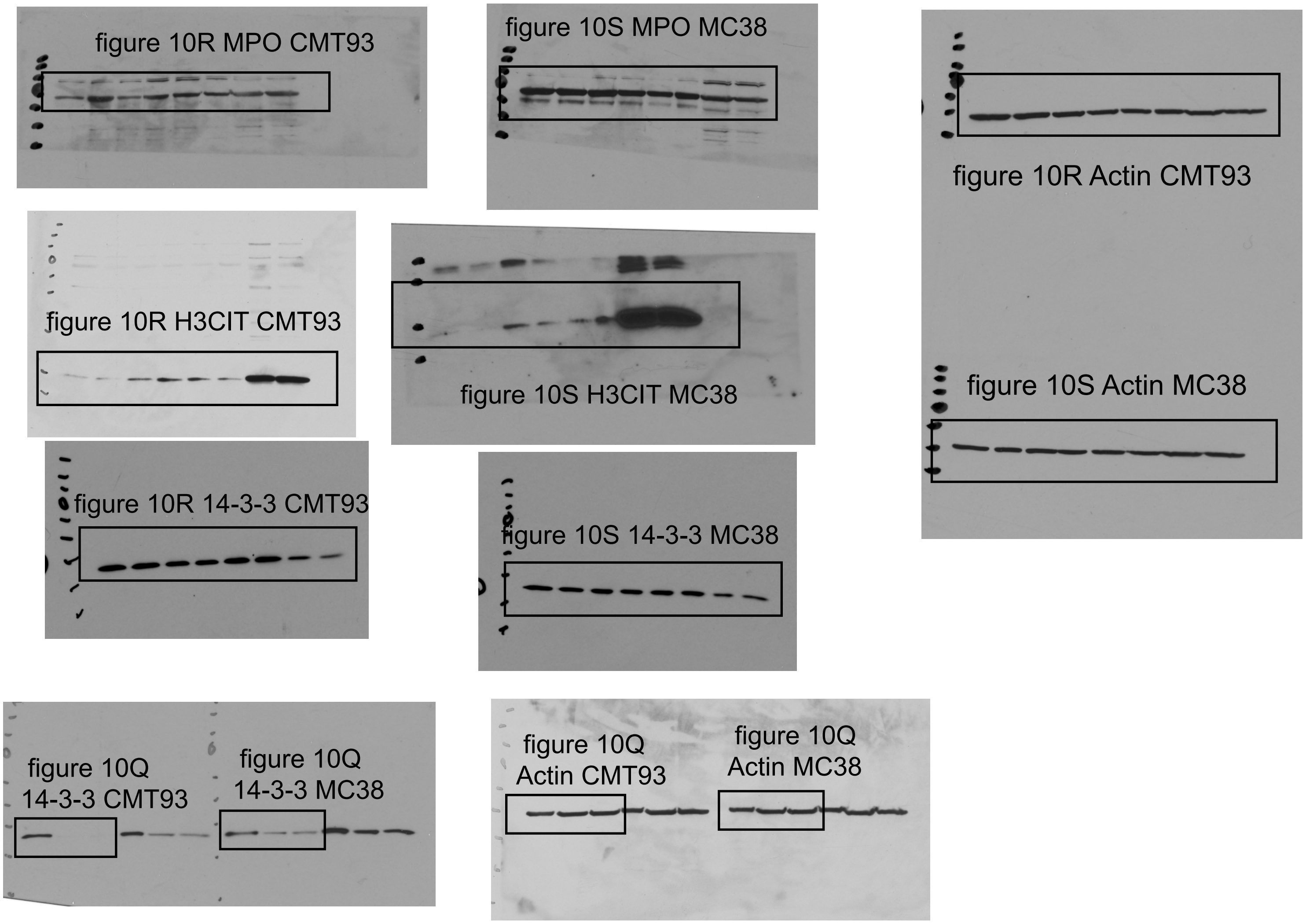

Full unedited gel for figure S2

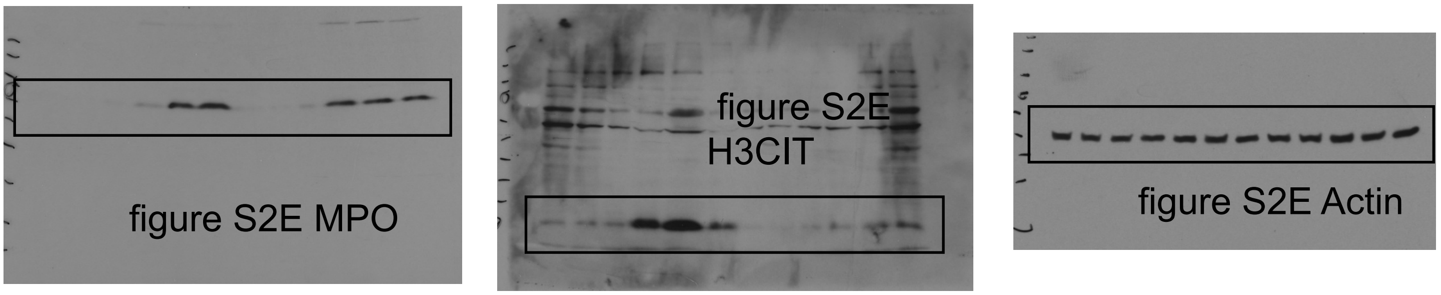

Full unedited gel for figure S3

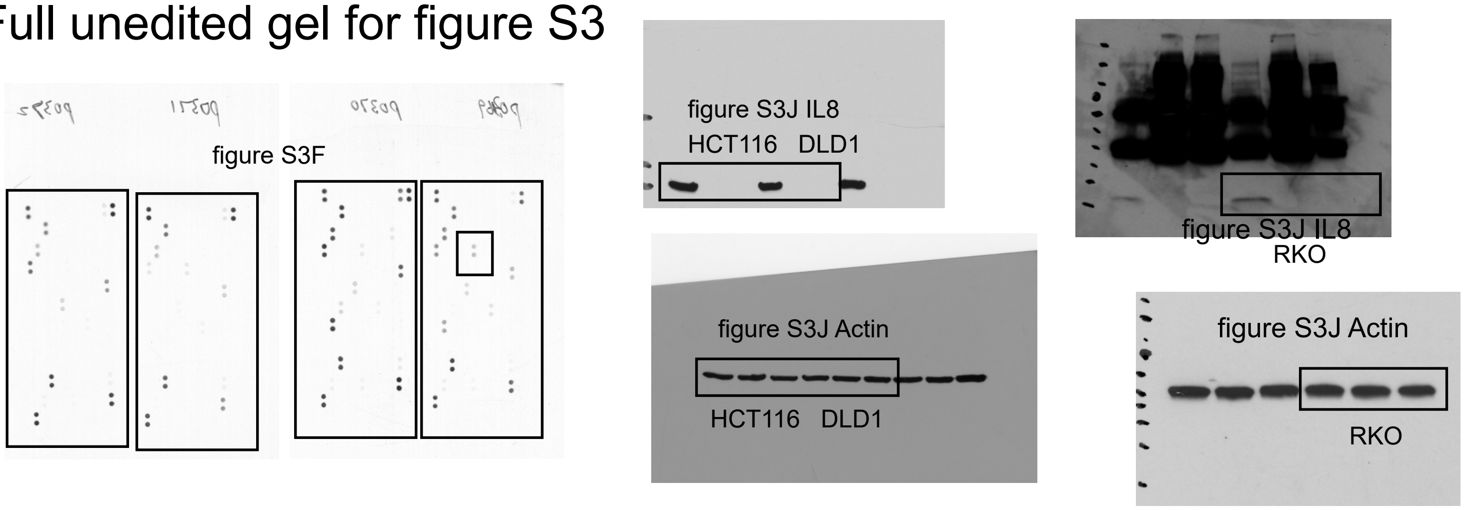

Full unedited gel for figure S3

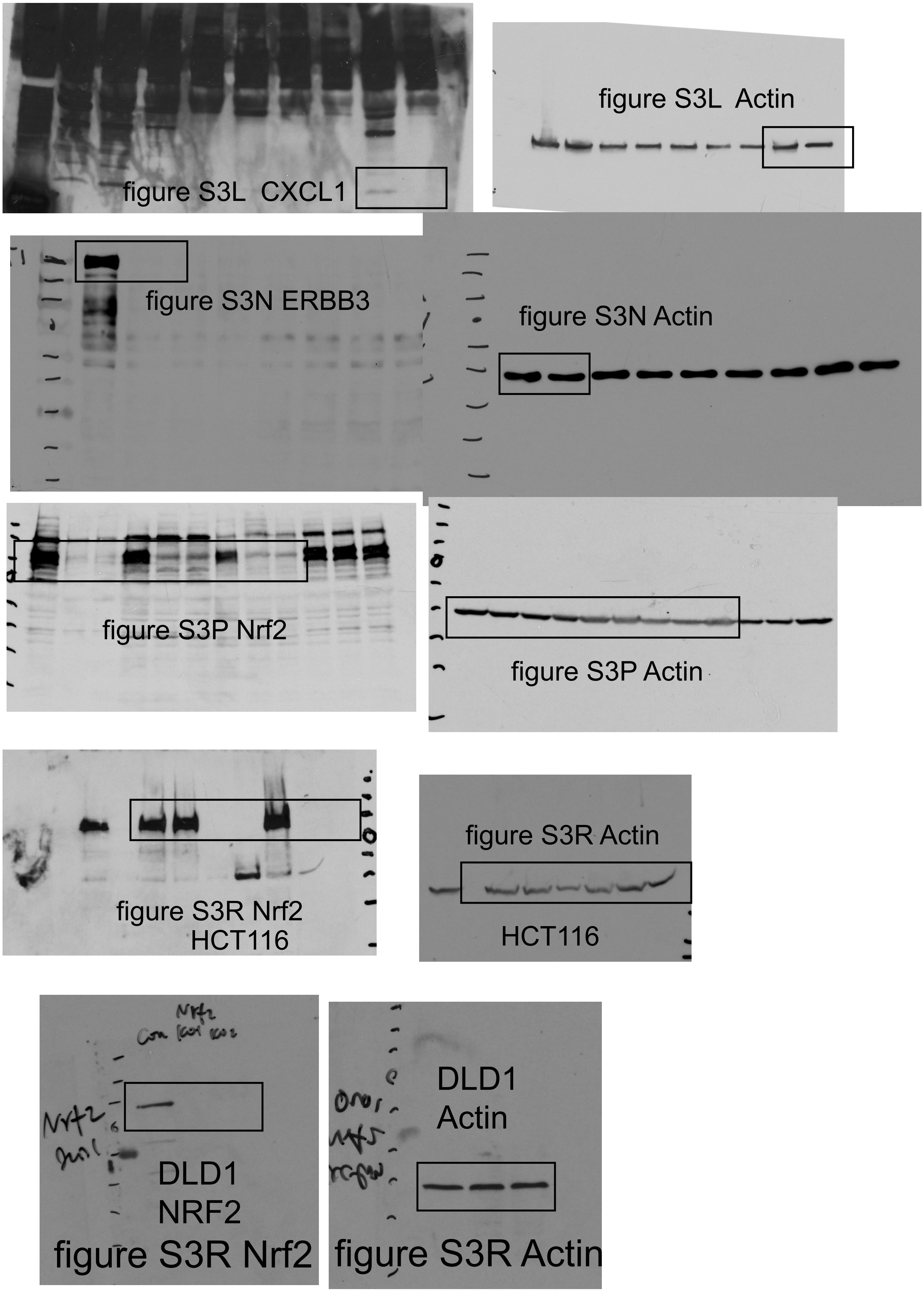

Full unedited gel for figure S5

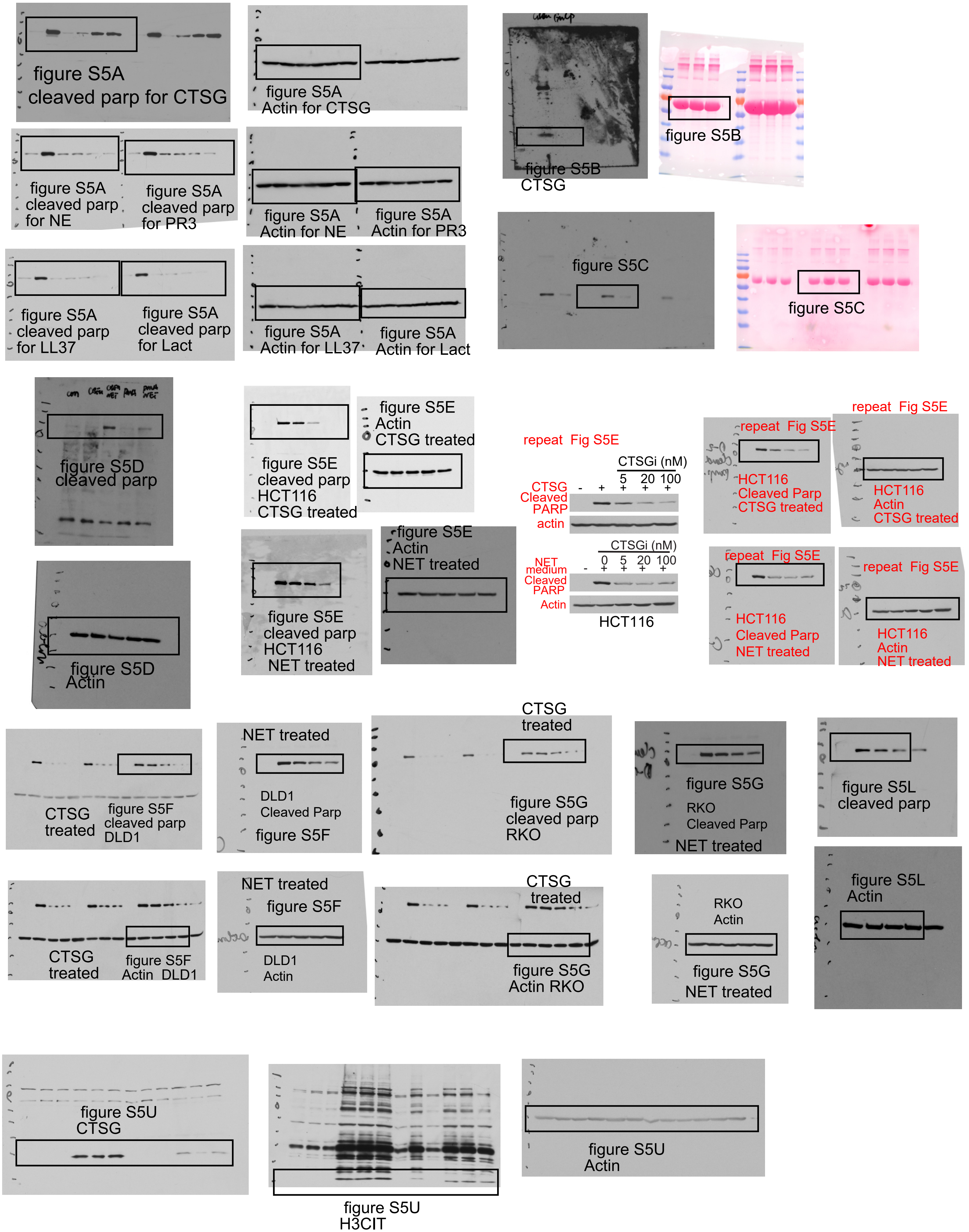

Full unedited gel for figure S6

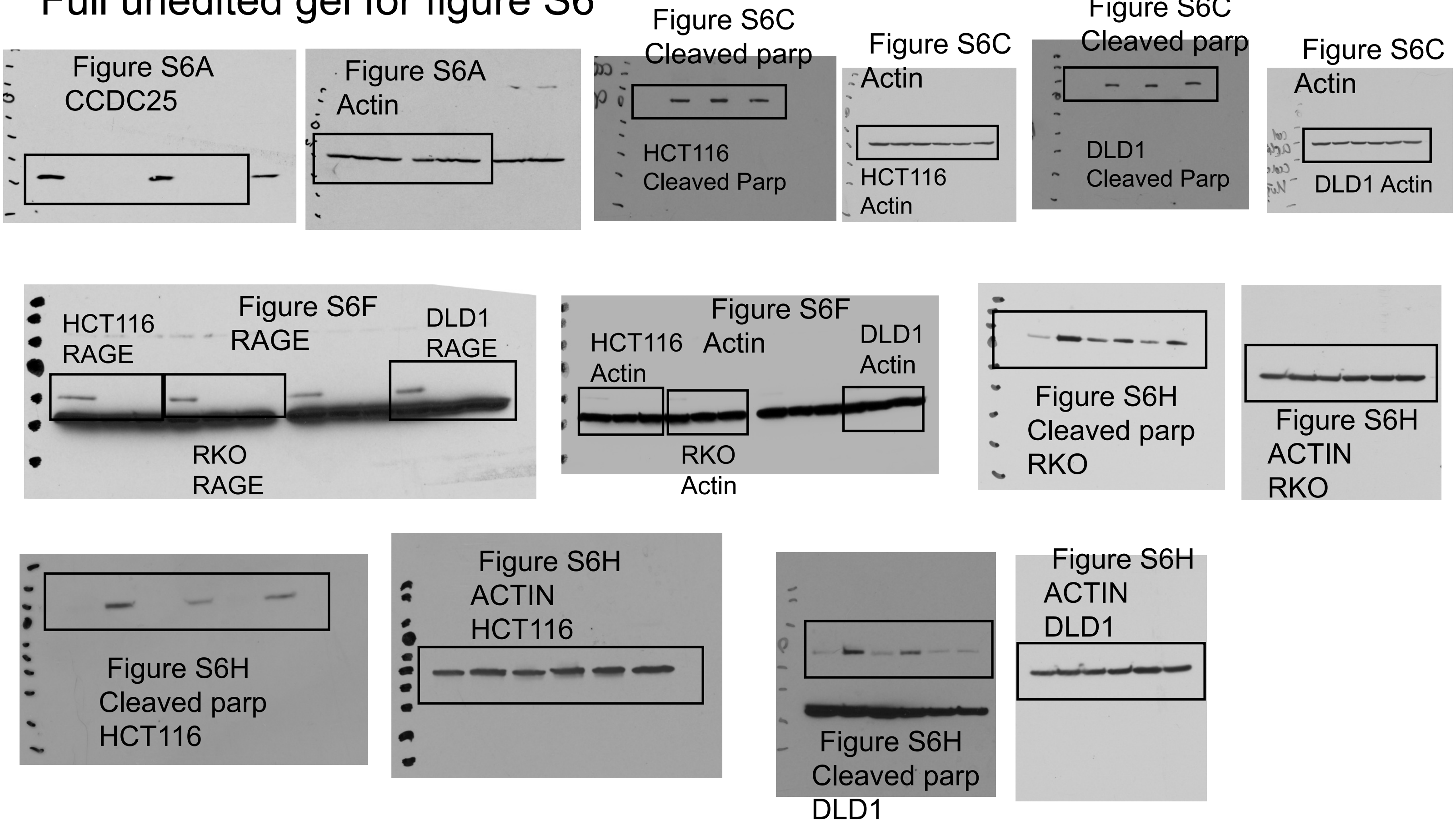

Full unedited gel for figure S7

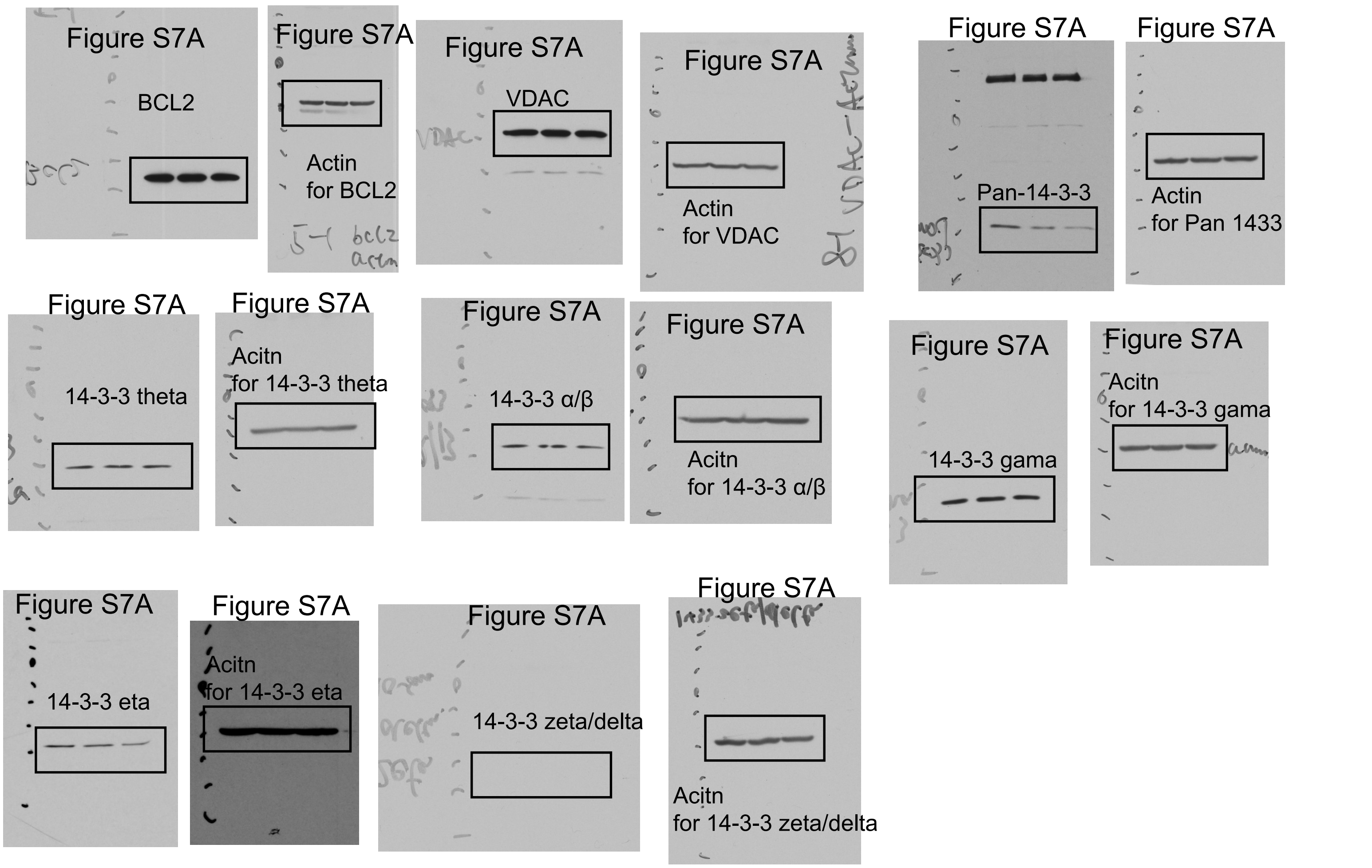

Full unedited gel for figure S7

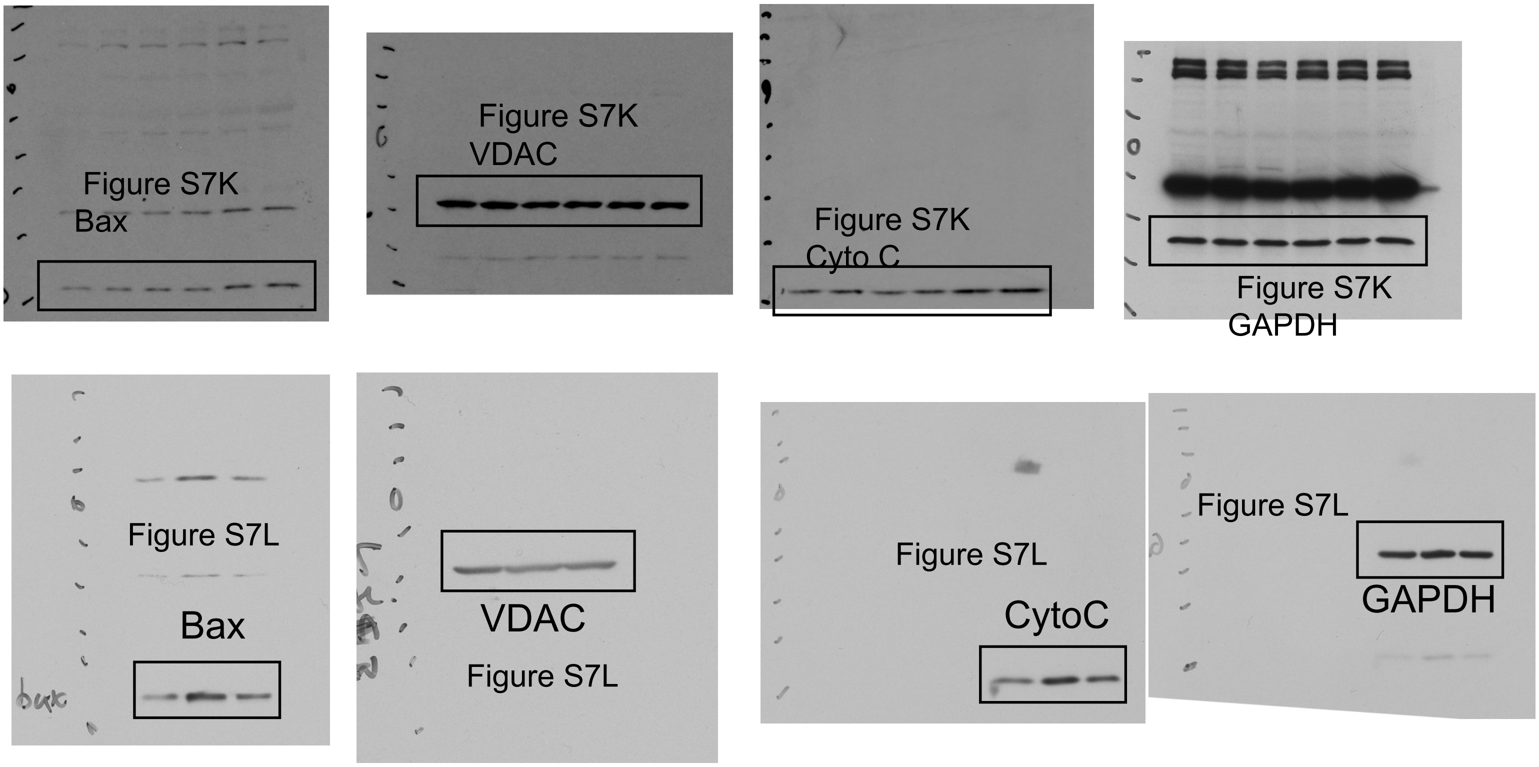

Full unedited gel for figure S9

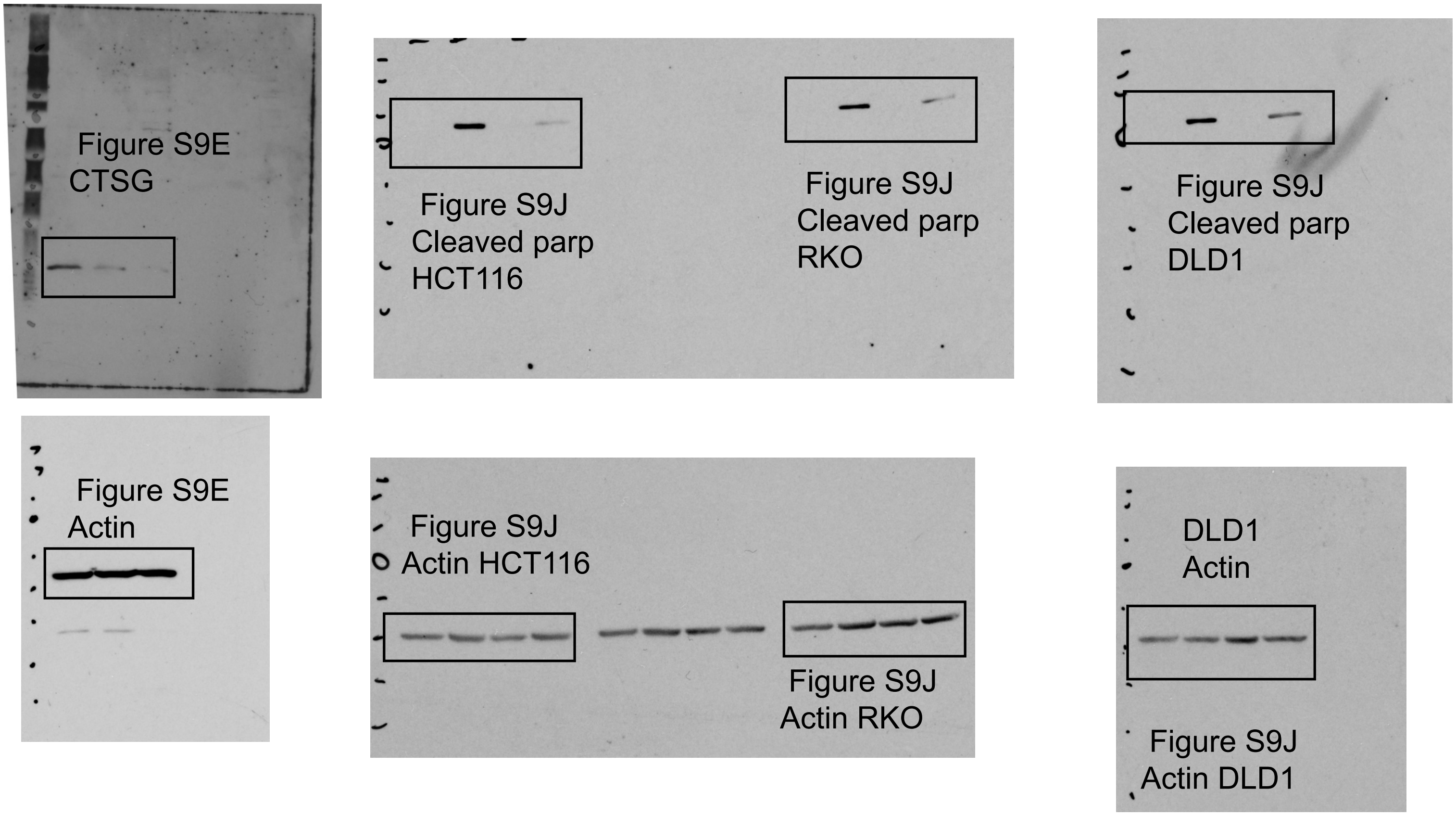

Supplement: Unedited blot and gel images [file jci-134-175031-s096.pdf]
